# Supplementary material for: Liver sinusoidal endothelial cells constitute a major route for hemoglobin clearance
Source: EMBO Rep. 2026 Jan 6;27(3):598–628. doi: 10.1038/s44319-025-00673-5 (PMC12895045; doi:10.1038/s44319-025-00673-5)

Appendix

This file contains Appendix Figures S1-S4 and Gating strategies for all figure panels.

Content	Page
Appendix Figure S1	2
Appendix Figure S2	2
Appendix Figure S3	3
Appendix Figure S4	3
Gating strategies:	4 - 14
Figures EV1A and 1B	4
Figures 1E and 2H	5
Figures 2A, 2B, 2G, 2J, EV1D EV2D-G	5
Figures 2I and 3	6
Figures 4C, 4D, 4I, 5N	7
Appendix Figure S1; Figure 4E, 4F and 5F (LSECs and KCs)	8
Figures 4E, 4F (spleen and heart ECs)	9
Figures 4G, 4J, 5L	10
Figure 5A	11
Figure 5B	12
Figure 5B and 5G	13
Figures 5J and EV4B, EV4E-F (spleen)	14
Figures 5M, 6D, 6G, 7B, 7E, EV4E-F (liver)	14

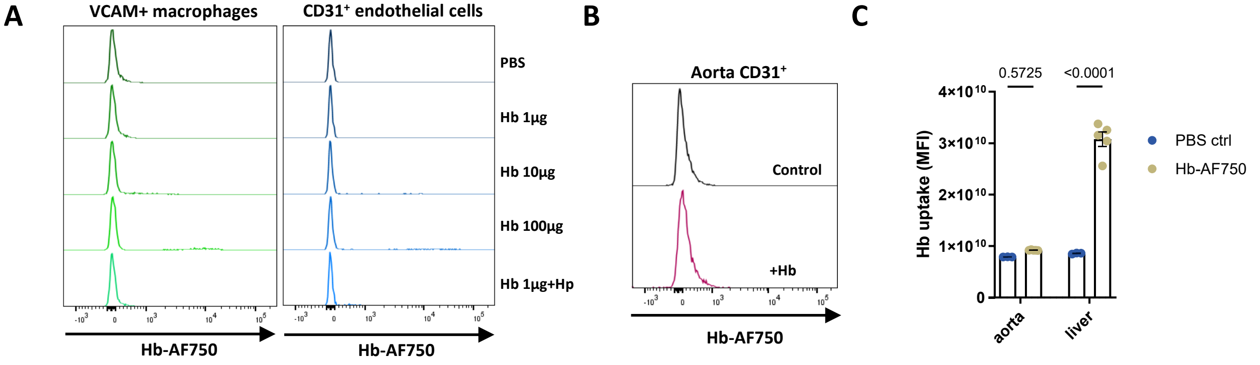

Appendix Figure S1. Bone marrow cells and aortic endothelium fail to sequester Hb.

Mice were injected i.v. with 1, 10, 100 µg of Hb-AF750 or Hb-AF750:Hp complex (1 µg: 32 µg) for 1 h. (A) Histograms of Hb-AF750 fluorescence in VCAM+ macrophages or CD31+ endothelial cells in the bone marrow. (B) Histograms of Hb-AF750 uptake by CD31+ endothelial cells from the aorta, 1 h after Hb-AF750 injection (10 µg/mouse). (C) Mice were injected with Hb-AF750 (10 µg/mouse) for 1 h, and livers and aortas were excised, and total fluorescence was measured by Bruker *in vivo* Imaging System. Quantification of the signal from Hb-AF750 in the organs is presented as total fluorescent counts. Data are expressed as mean ± SEM, and each data point represents one biological replicate. Two-way ANOVA with Tukey's Multiple Comparison tests was used to determine statistical significance in C; exact p-values are shown on the graph.

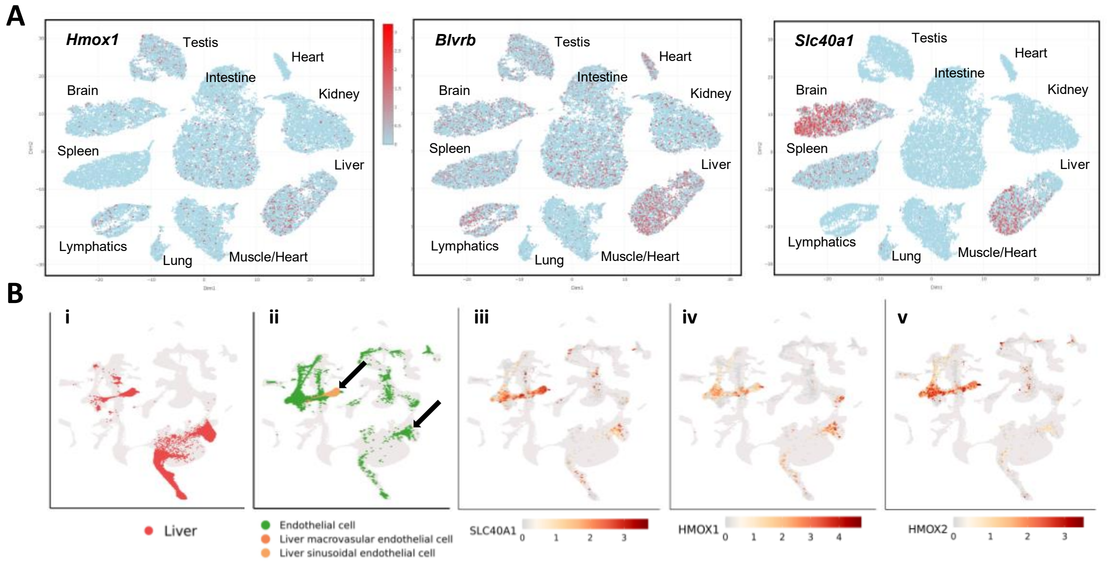

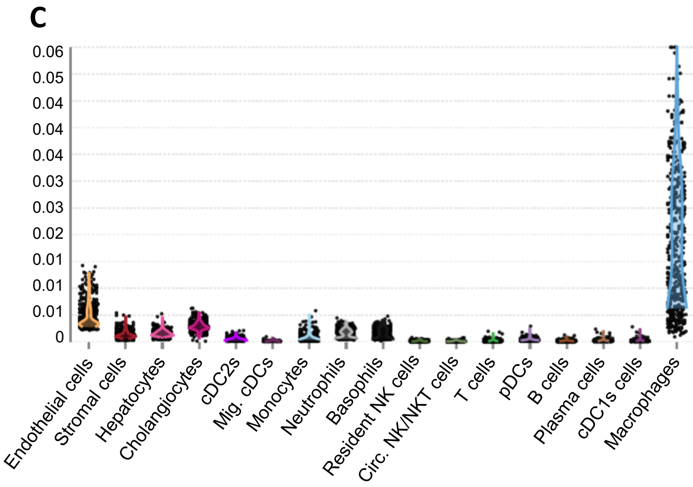

Appendix Figure S2. Liver endothelial cells are distinguished by high mRNA expression levels of genes involved in iron recycling.

(A) Visualization of the expression levels of *Hmox1*, *Blvrb*, and *Slc40a1* obtained with EC Atlas, which collects data from single-cell RNA sequencing of endothelial cells (ECs) from different mouse organs (Kalucka et al. 2020). (B) Analysis of single-cell transcriptome atlas of pig tissues: i) indicates all liver cells, ii) depicts ECs, including those in the liver – LSECs and macrovascular ECs. Arrows point to liver ECs. iii-v) visualization of the expression levels of *Slc40a1*, *Hmox1,* and *Hmox2*, respectively, in cells selected in ii) (The Pig Single Cell RNA Atlas; Wang et al. 2022). (C) Endothelial cells show second-highest expression of *Slc40a1* among all liver cells, as determined by single-cell RNA seq (Liver Cell Atlas; Guilliams et al. 2022).

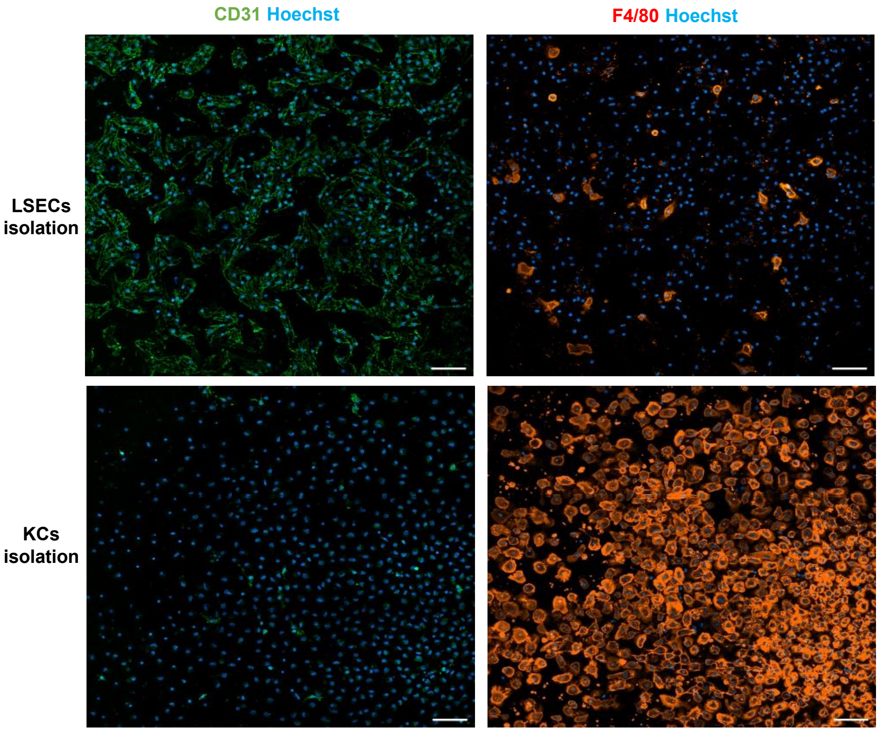

Appendix Figure S3. Purity of magnetically-sorted LSECs and KCs.

Freshly isolated murine NPCs were subjected to magnetic separation, aimed at LSECs or KCs separation. Cells were plated, fixed, and stained for CD31 (green), F4/80 (orange), and nuclei (Hoechst, blue), and evaluated for purity using confocal microscopy. Scale bars, 20 µm.

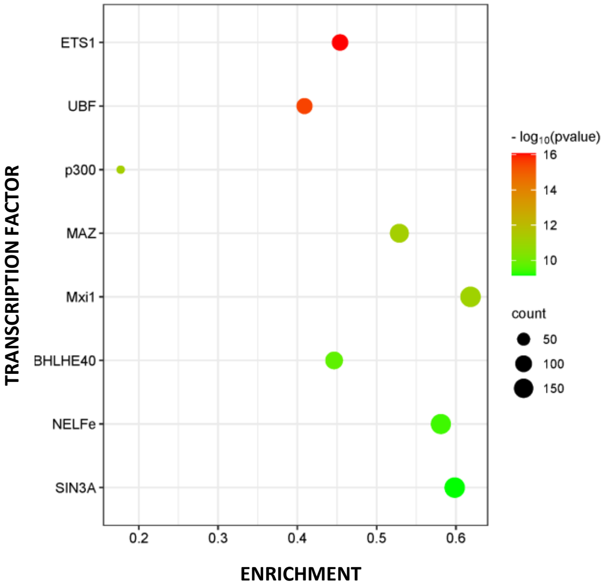

Appendix Figure S4. ETS1 emerged as a key transcription factor responsible for the LSEC transcriptome response upon Hb injection.

The set of genes induced in FACS-sorter LSECs upon injection of mice with 10 mg of Hb was compared with a genome-wide ChIP-seq dataset using the Cscan program. Potential common transcriptional regulators of input genes are shown.

Flow Cytometry Gating Strategies

This file gathers all gating strategies assigned to individual figure panels, as indicated.

Gating strategy for Figure EV1A

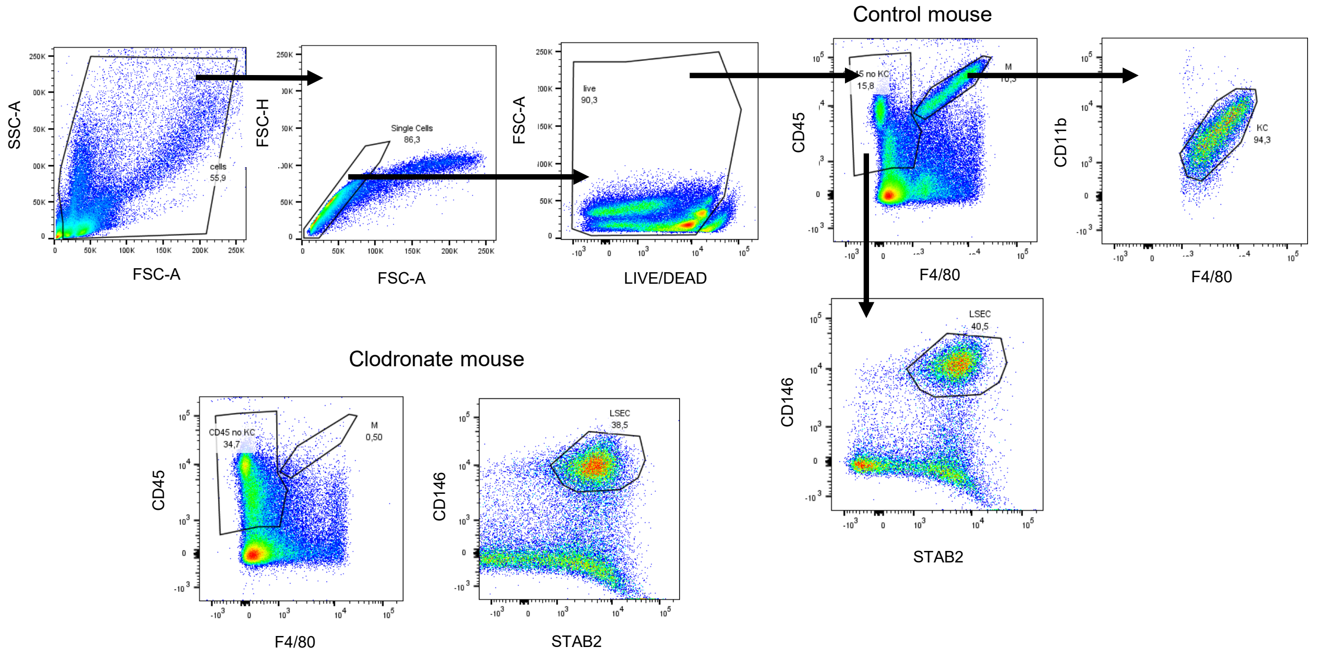

Gating strategy for Figure 1B

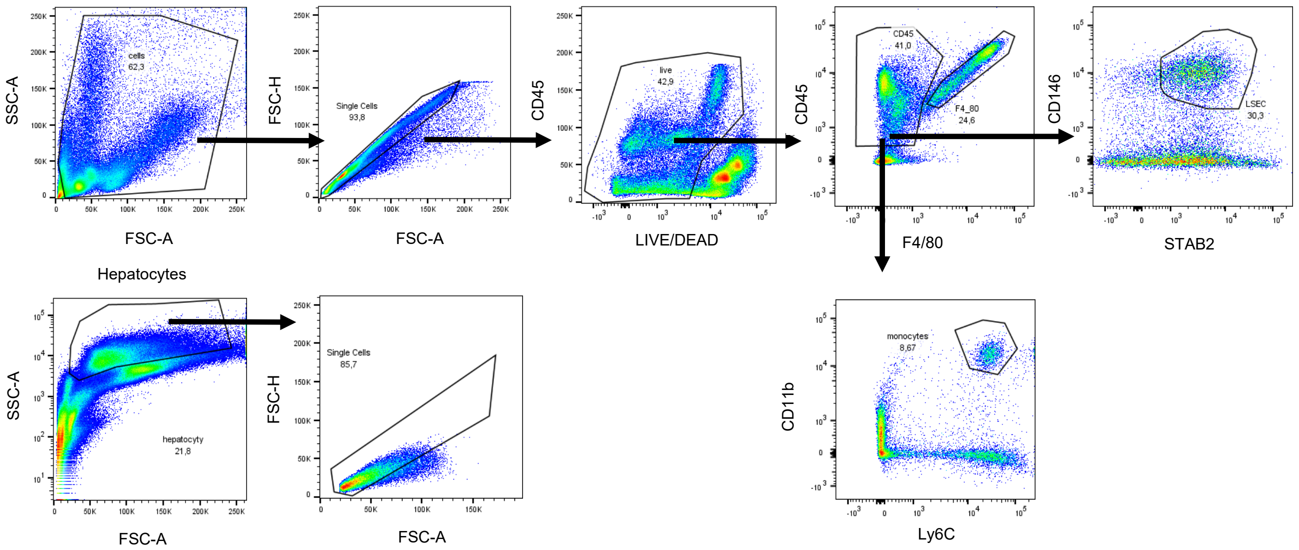

Gating strategy for Figures 1E and 2H (primary human liver NPCs cells)

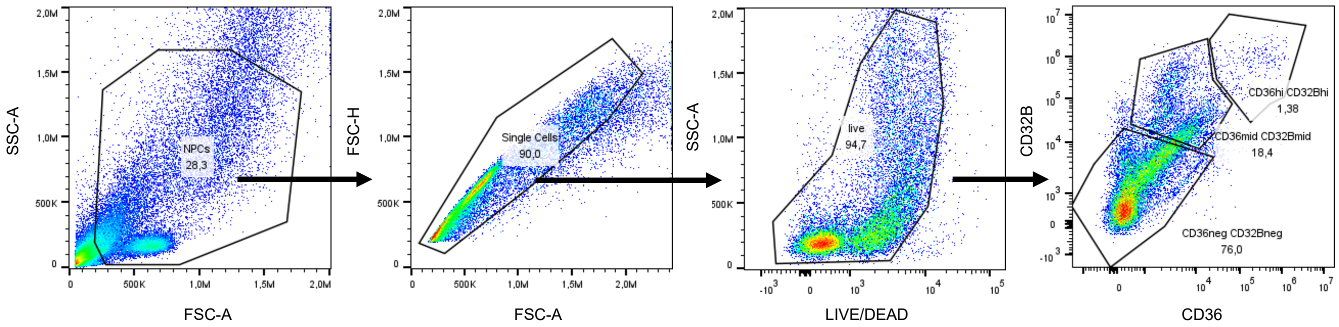

Gating strategy for Figures 2A, 2B, 2G, 2J, EV1D and EV2D-G (primary murine liver NPCs)

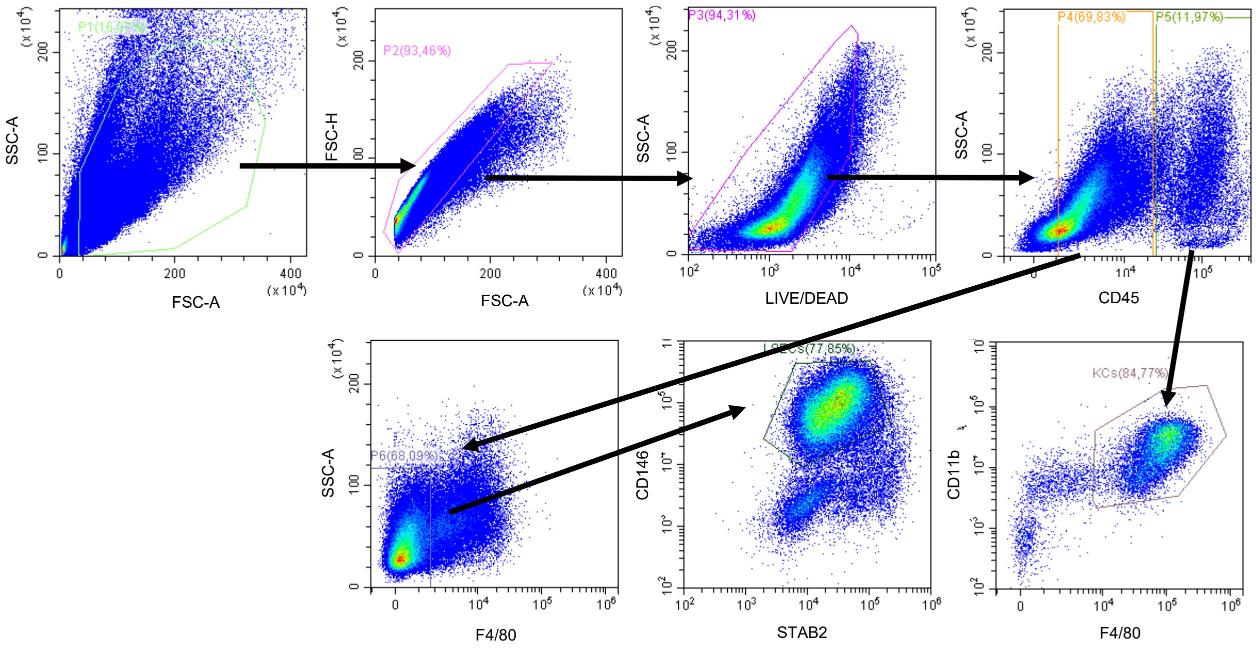

Gating strategy for Figure 2I

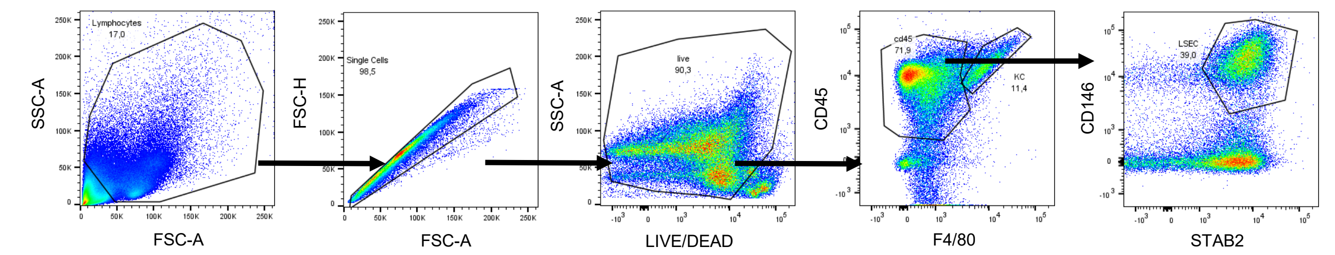

Gating strategy for Figures 3A-D

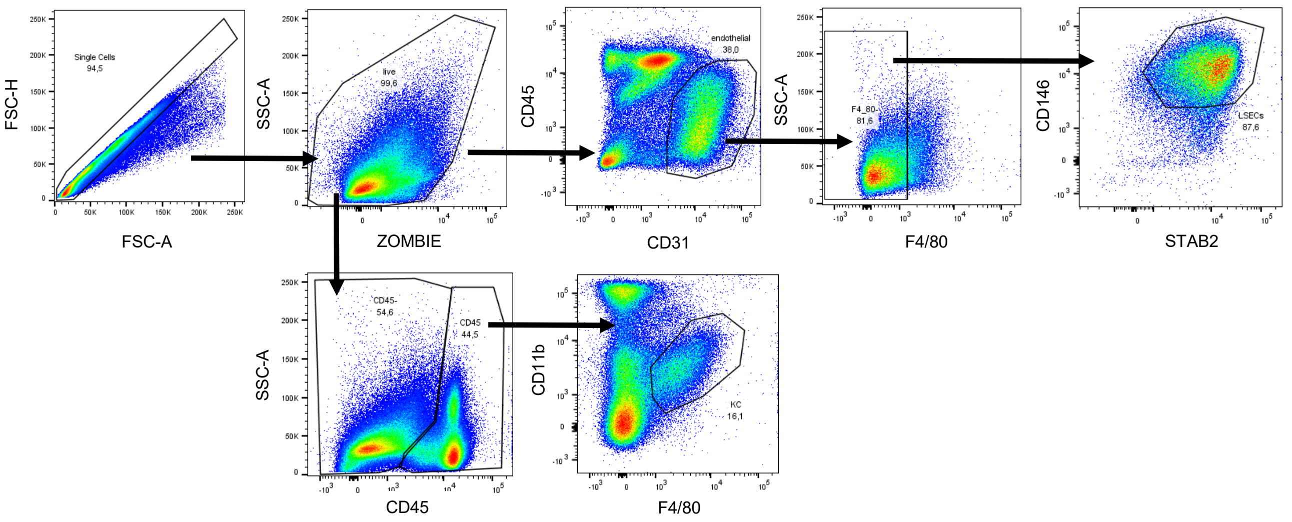

Gating strategy for Figures 3E-H

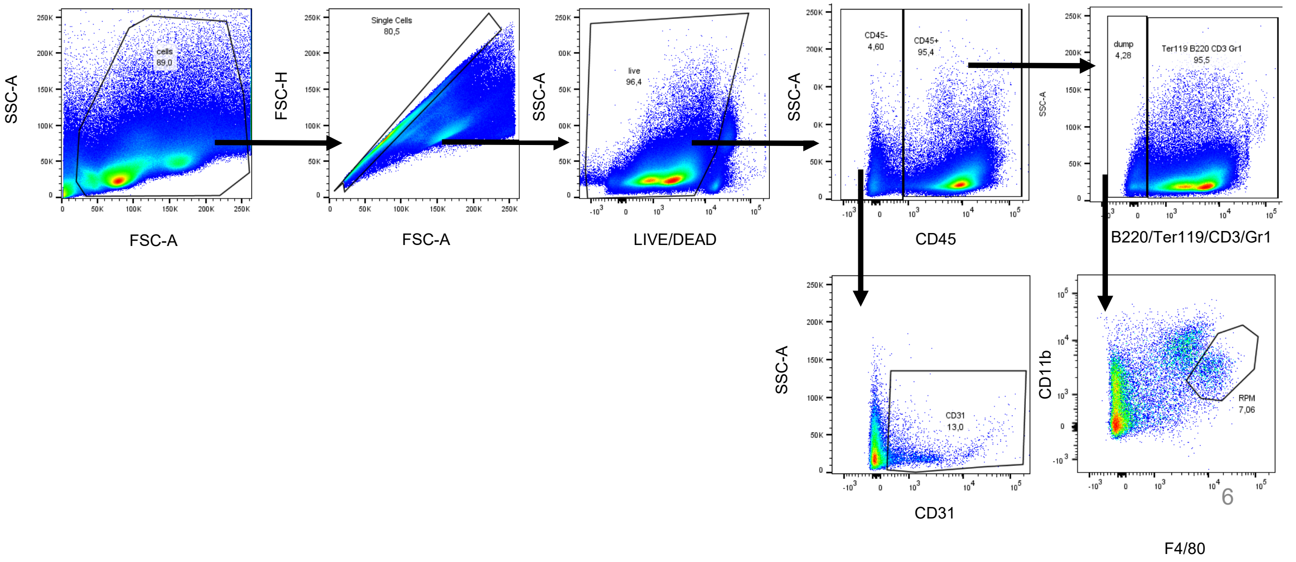

Gating strategy for Figures 4C and 4D

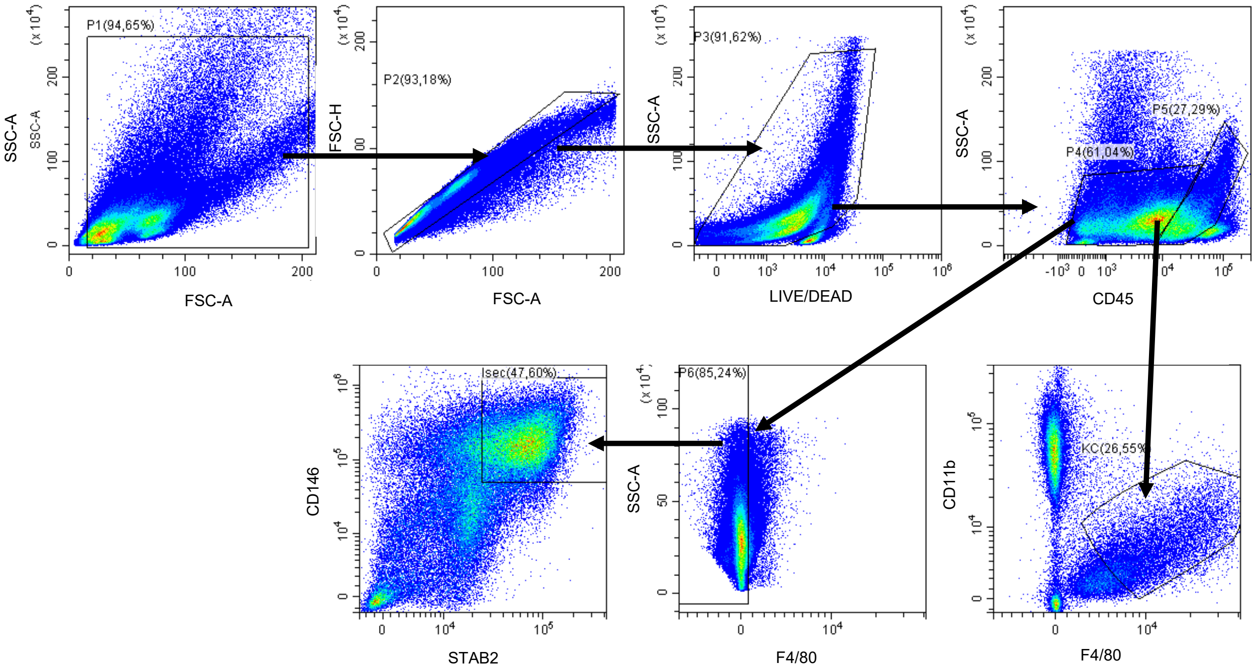

Gating strategy for Figures 4I and Figure 5N

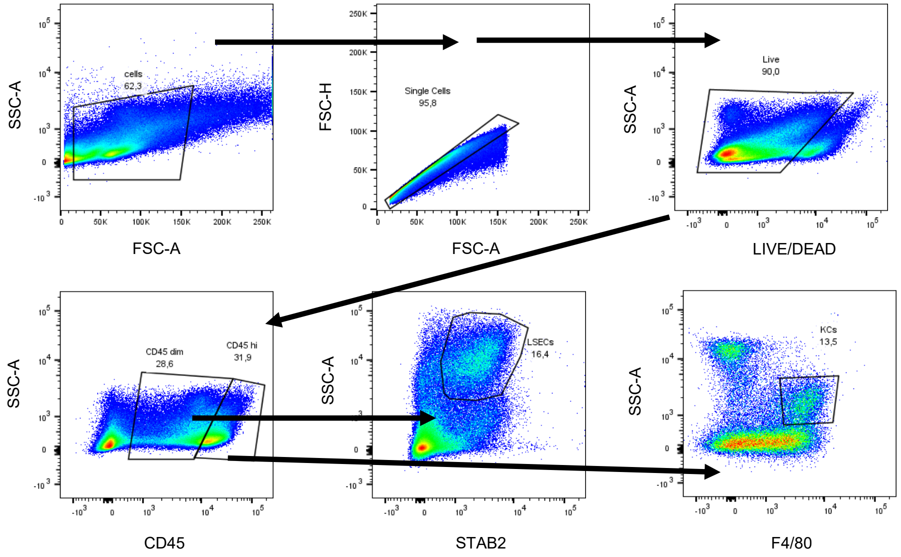

Gating strategy for Appendix Figure S1A (bone marrow)

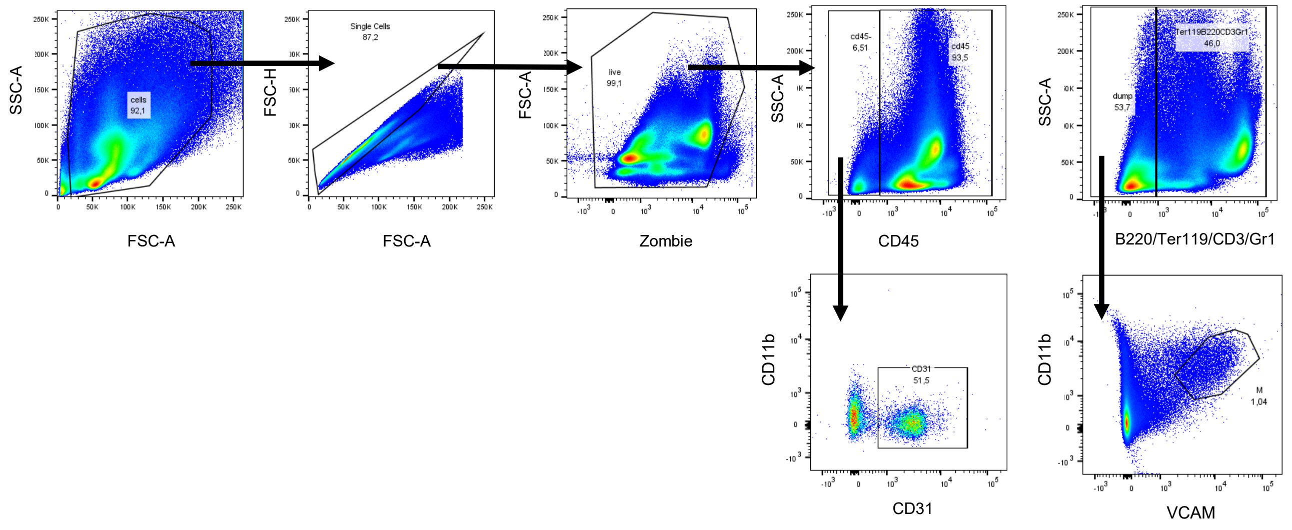

Gating strategy for Appendix Figure S1B (aorta)

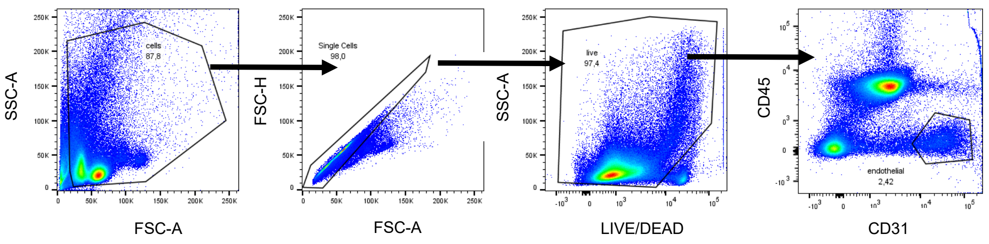

Gating strategy for Figure 4E, 4F and 5F – sorting LSECs and KCs

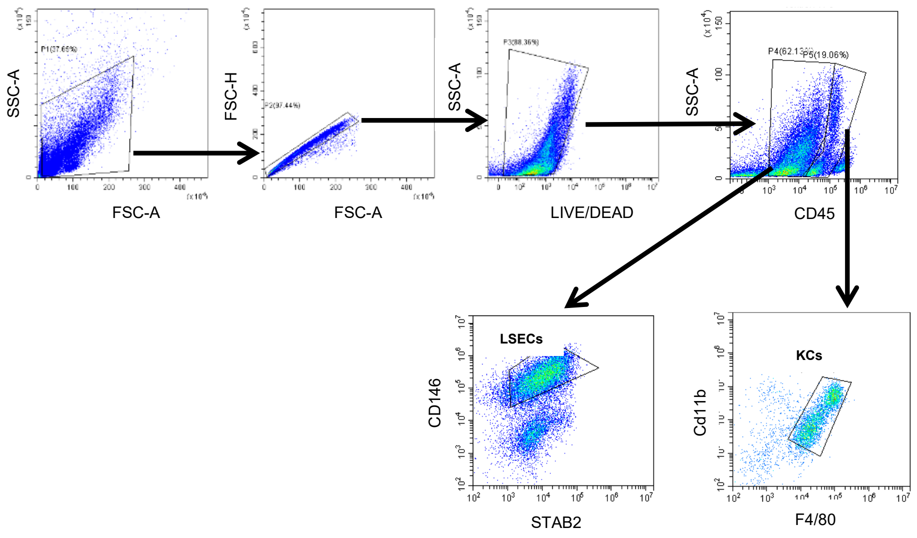

Gating strategy for Figures 4E and 4F (sorting spleen endothelial cells)

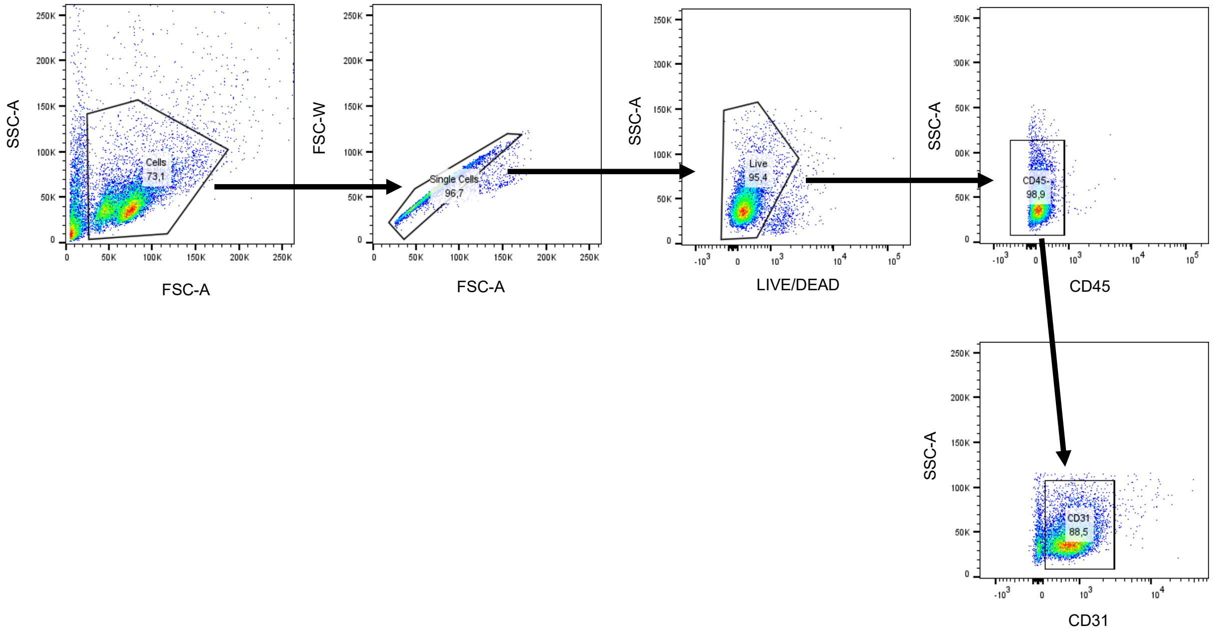

Gating strategy for Figures 4E and 4F (sorting heart endothelial cells)

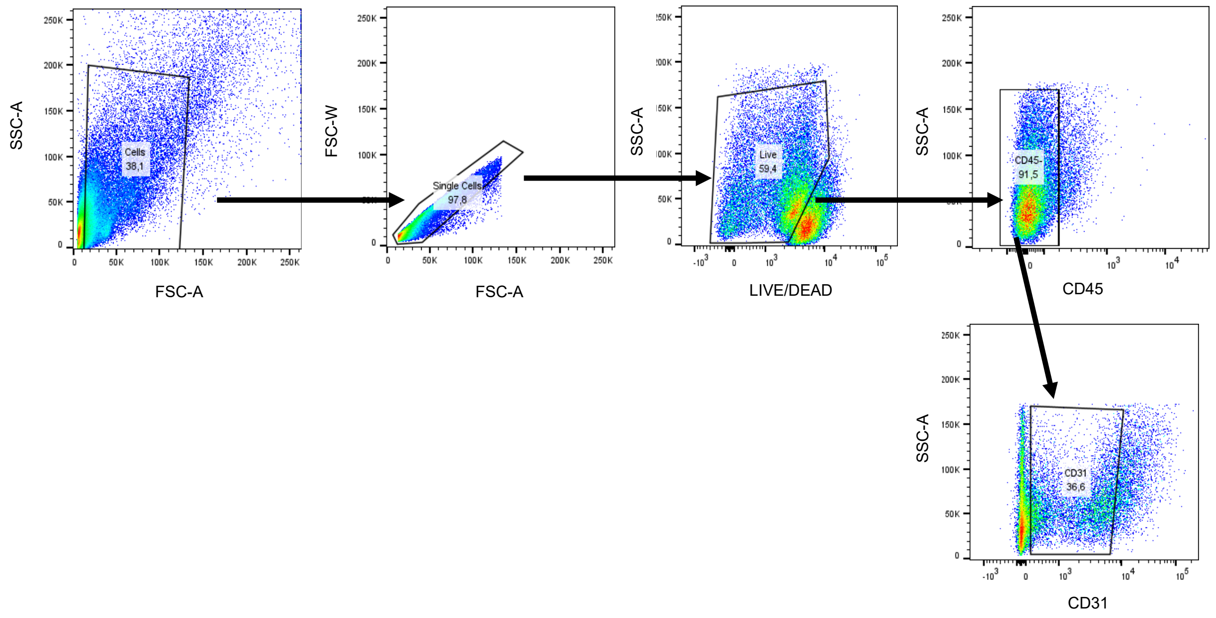

Gating strategy for Figure 4G (liver), 4J and 5L (Ferroorange)

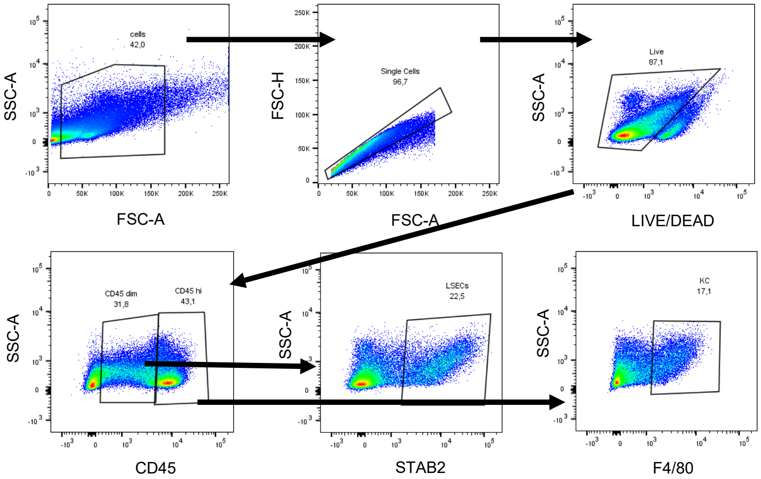

Gating strategy for Figure 4G (spleen)

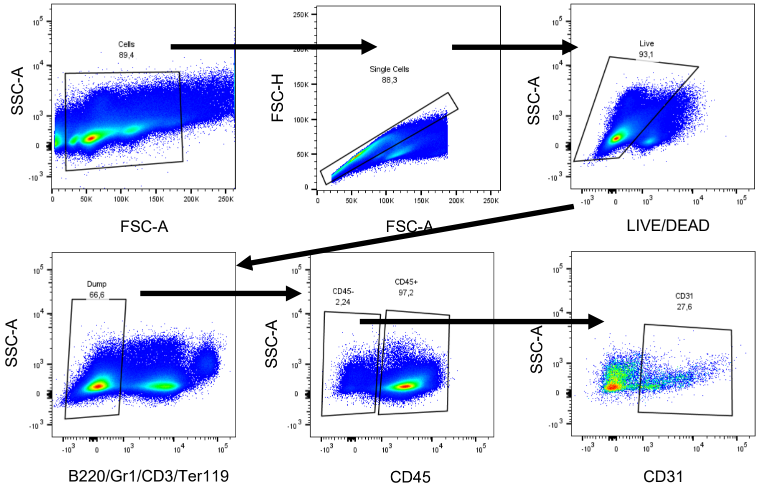

Gating strategy for Figure 4G (bone marrow)

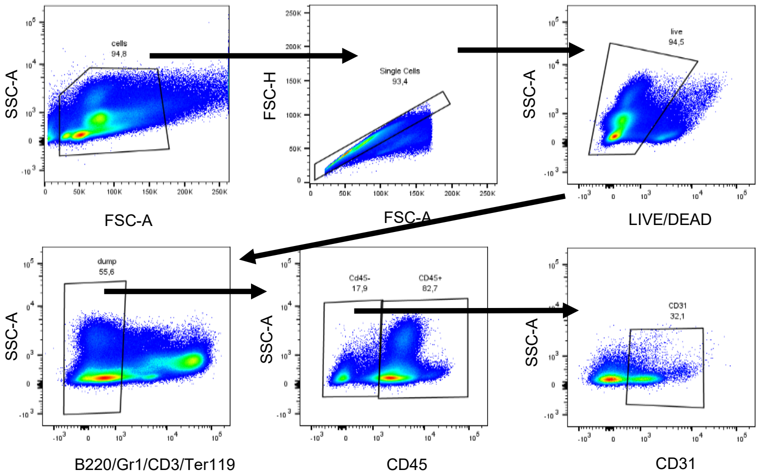

Gating strategy for Figure 5A (spleen control mouse)

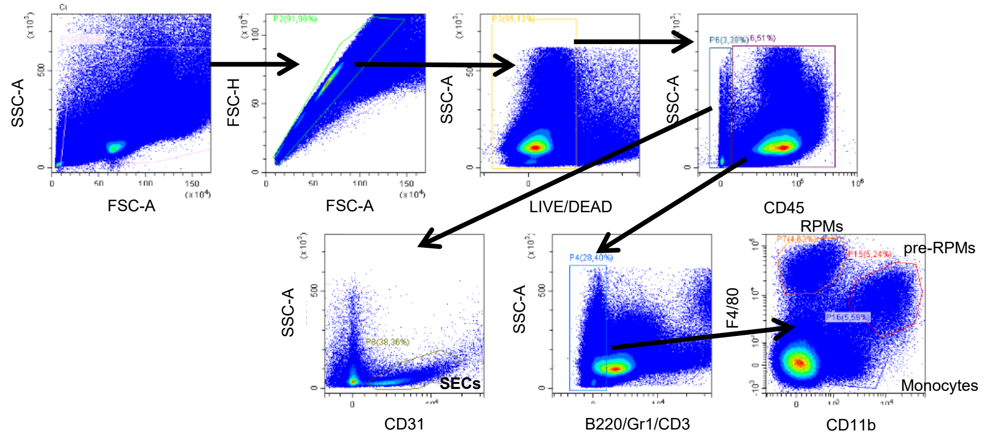

Gating strategy for Figure 5A (spleen + GFP RBCs)

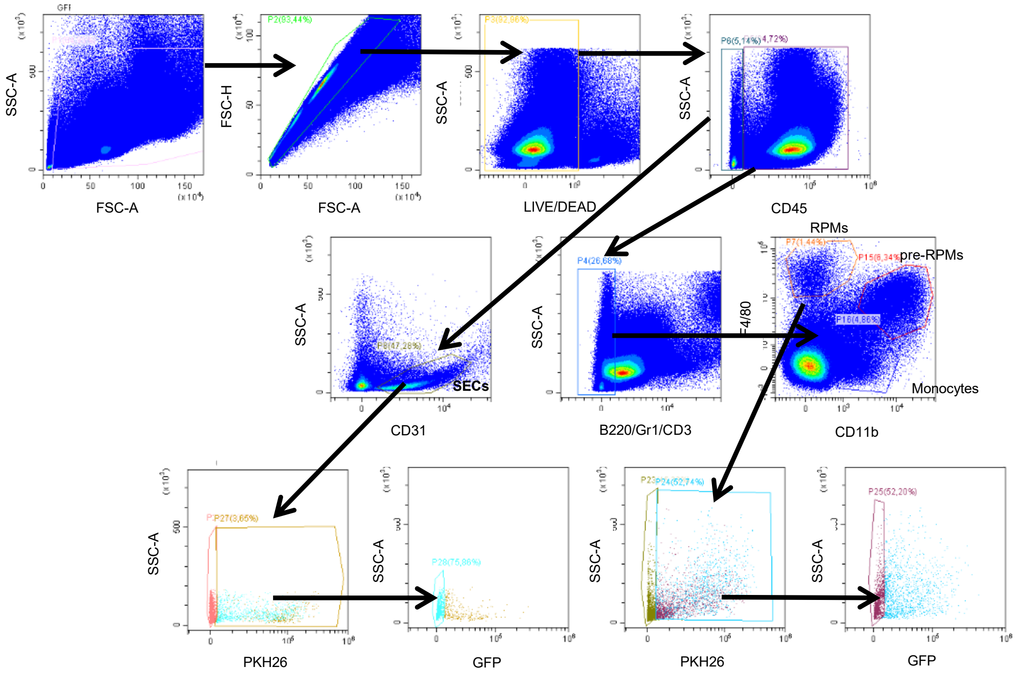

Gating strategy for Figure 5A (spleen dendritic cells)

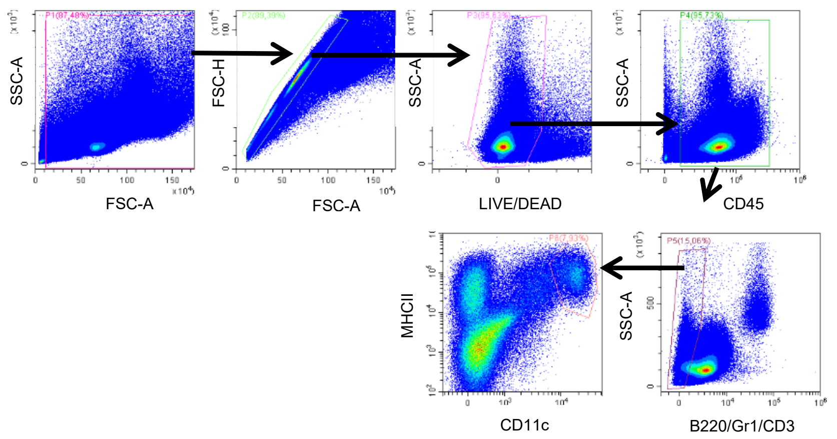

Gating strategy for Figure 5B (liver control mouse)

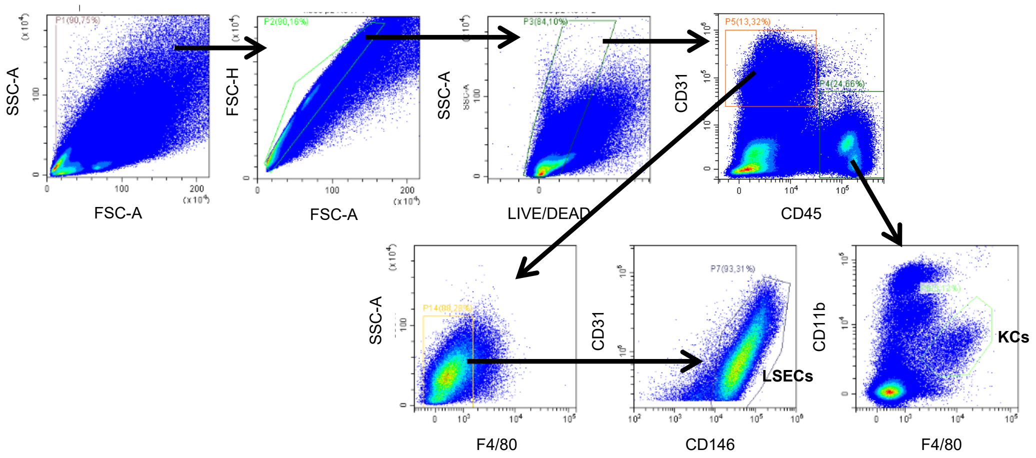

Gating strategy for Figure 5B (liver + GFP RBCs)

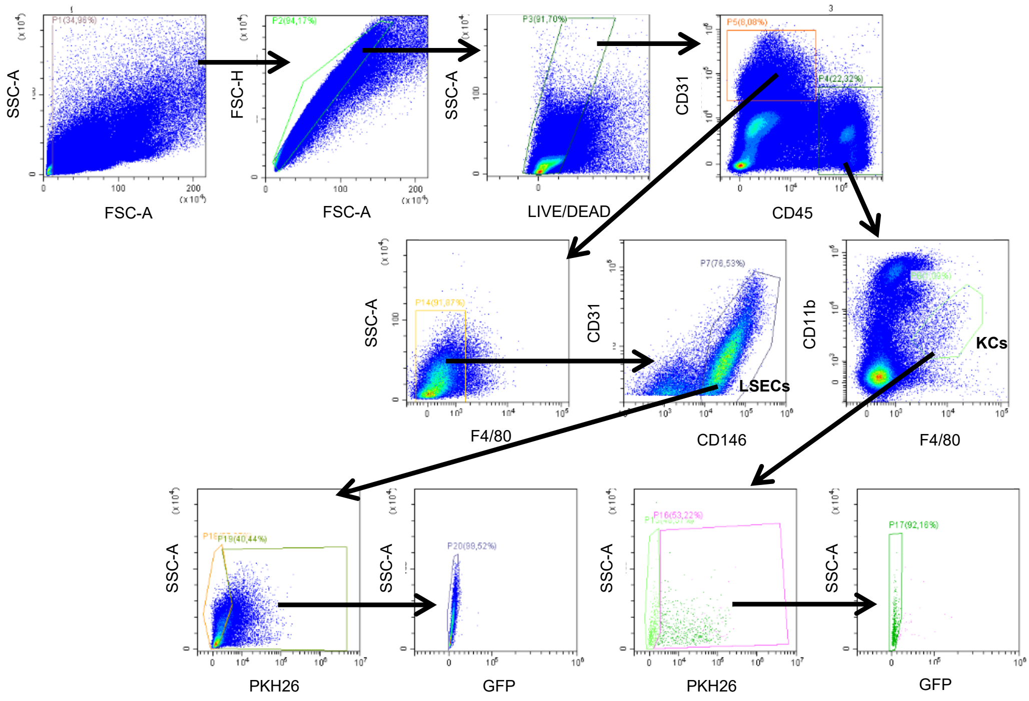

Gating strategy for Figure 5B (liver dendritic cells)

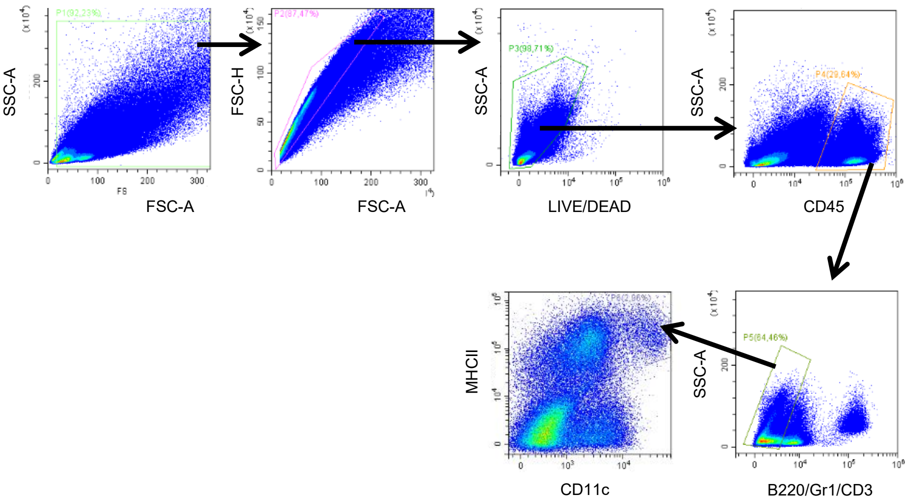

Gating strategy for Figure 5G

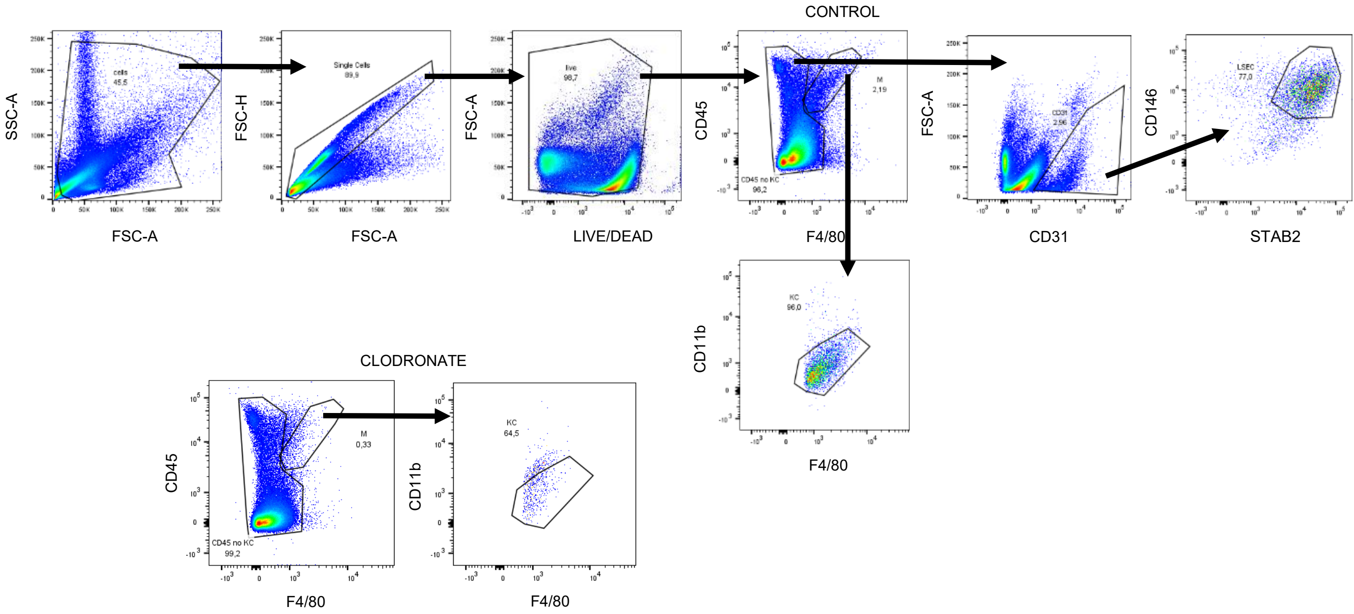

Gating strategy for Figures 5J and EV4B, E and F

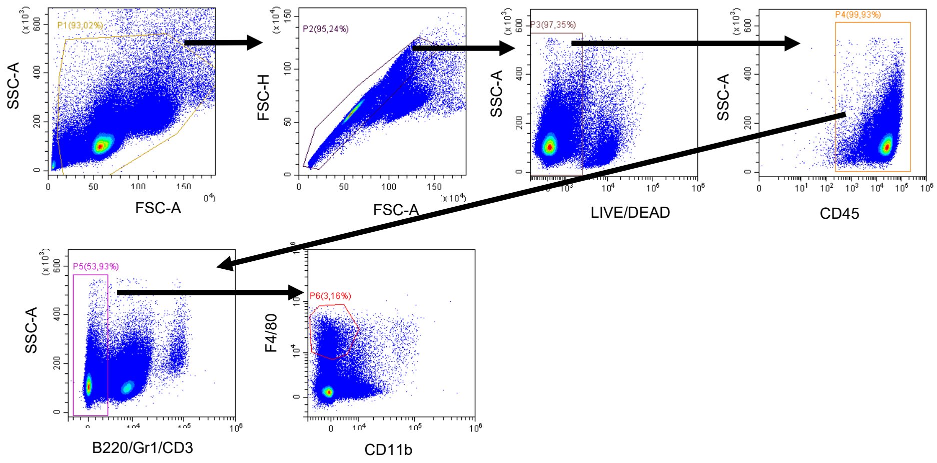

Gating strategy for Figures 5M, 6D, 6G, 7B, 7E, EV4E-F (liver cell flow analysis and FACS sorting for gene expression)

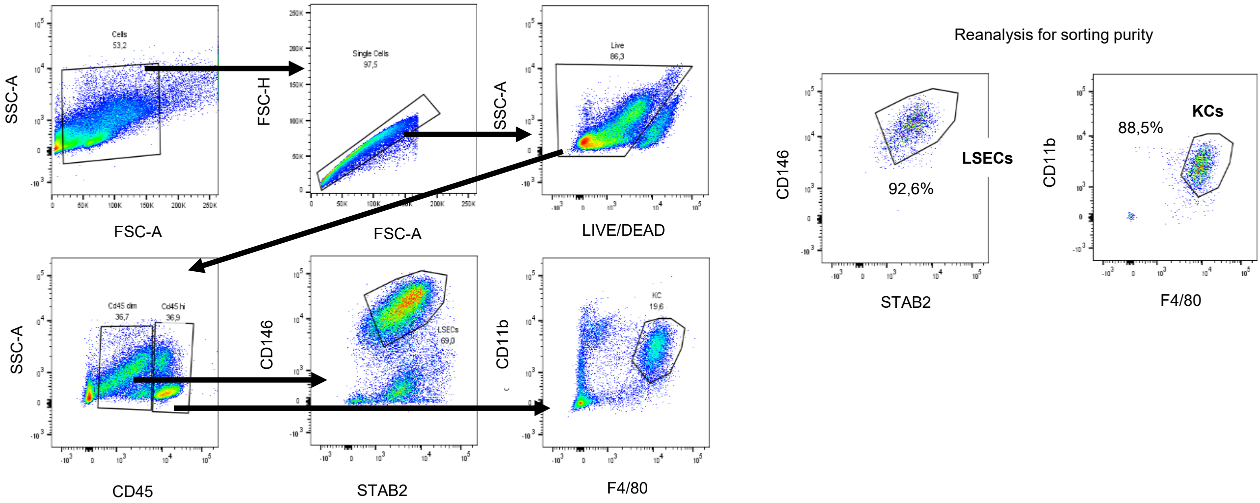

Supplement: Supplementary file 1 — Appendix [file 44319_2025_673_MOESM1_ESM.pdf]
