# Supplementary material for: Liver sinusoidal endothelial cells constitute a major route for hemoglobin clearance
Source: EMBO Rep. 2026 Jan 6;27(3):598–628. doi: 10.1038/s44319-025-00673-5 (PMC12895045; doi:10.1038/s44319-025-00673-5)
Supplement: Supplementary file 5 — Source data Fig. 1 [file 44319_2025_673_MOESM5_ESM.zip › Figure 1/1D/CTR/README.docx]

The images were processed using ImageJ software with linear adjustments of contrast and brightness, equally across the whole image area and in comparison to the blank samples. The yellow color corresponding to the AF555 (F4/80) has been changed to blue pseudocolor.
