# Supplementary figures and images for: Liver sinusoidal endothelial cells constitute a major route for hemoglobin clearance
Source: EMBO Rep. 2026 Jan 6;27(3):598–628. doi: 10.1038/s44319-025-00673-5 (PMC12895045; doi:10.1038/s44319-025-00673-5)

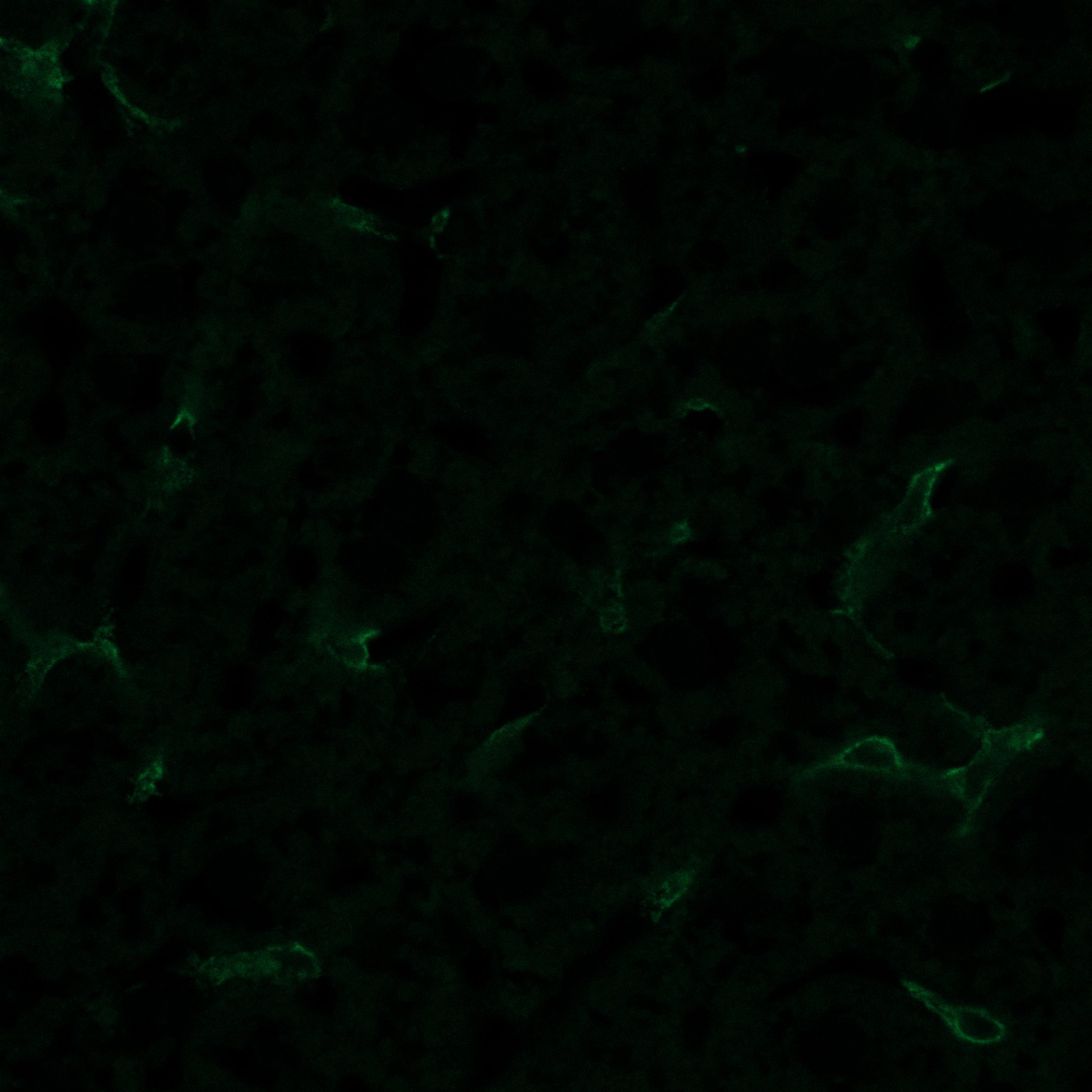

Supplement: Supplementary file 5 — Source data Fig. 1 [file 44319_2025_673_MOESM5_ESM.zip › Figure 1/1C/KCs/F480 staining_KCs.jpg]

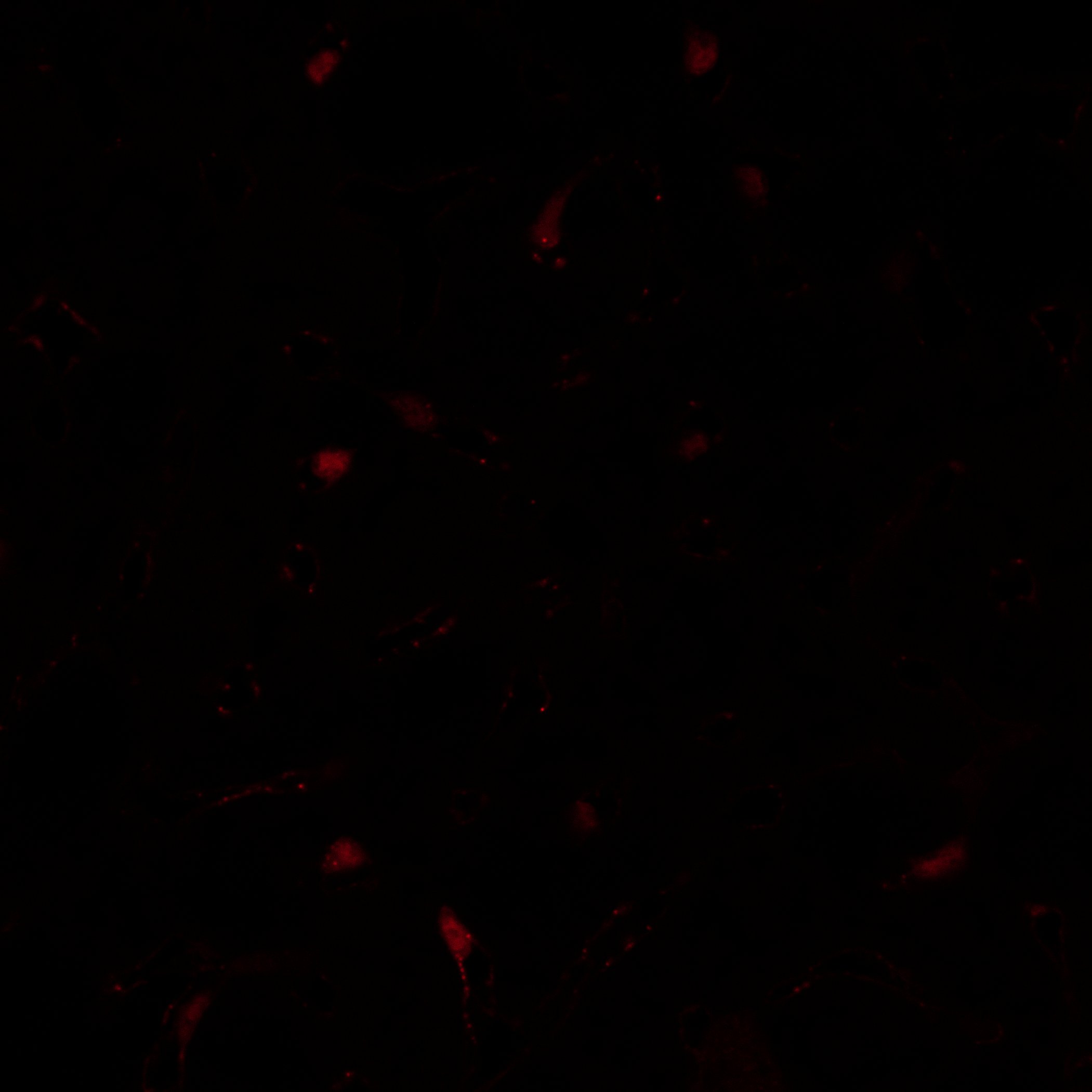

Supplement: Supplementary file 5 — Source data Fig. 1 [file 44319_2025_673_MOESM5_ESM.zip › Figure 1/1C/KCs/Hgb_KCs.jpg]

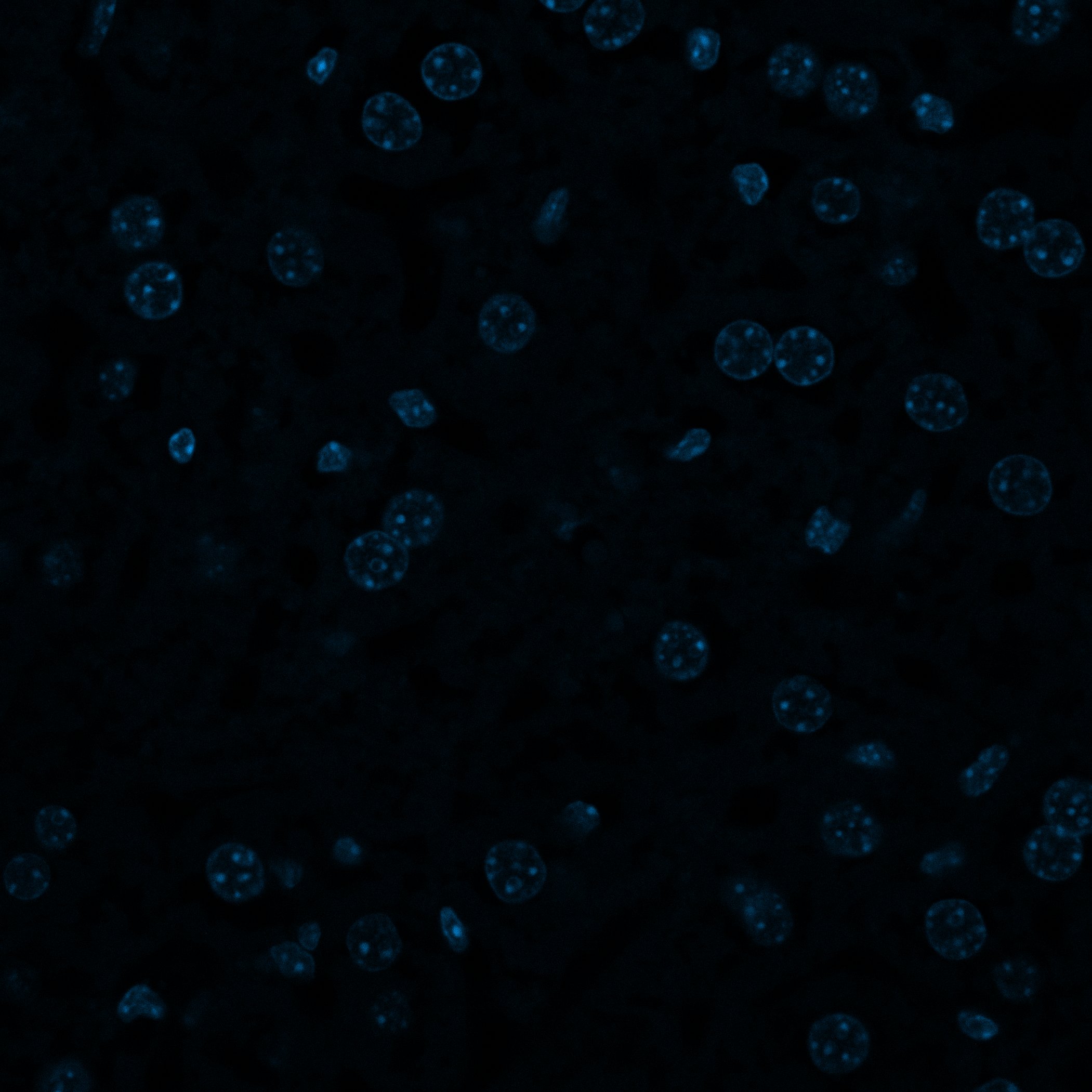

Supplement: Supplementary file 5 — Source data Fig. 1 [file 44319_2025_673_MOESM5_ESM.zip › Figure 1/1C/KCs/Hoechst staining_KCs.jpg]

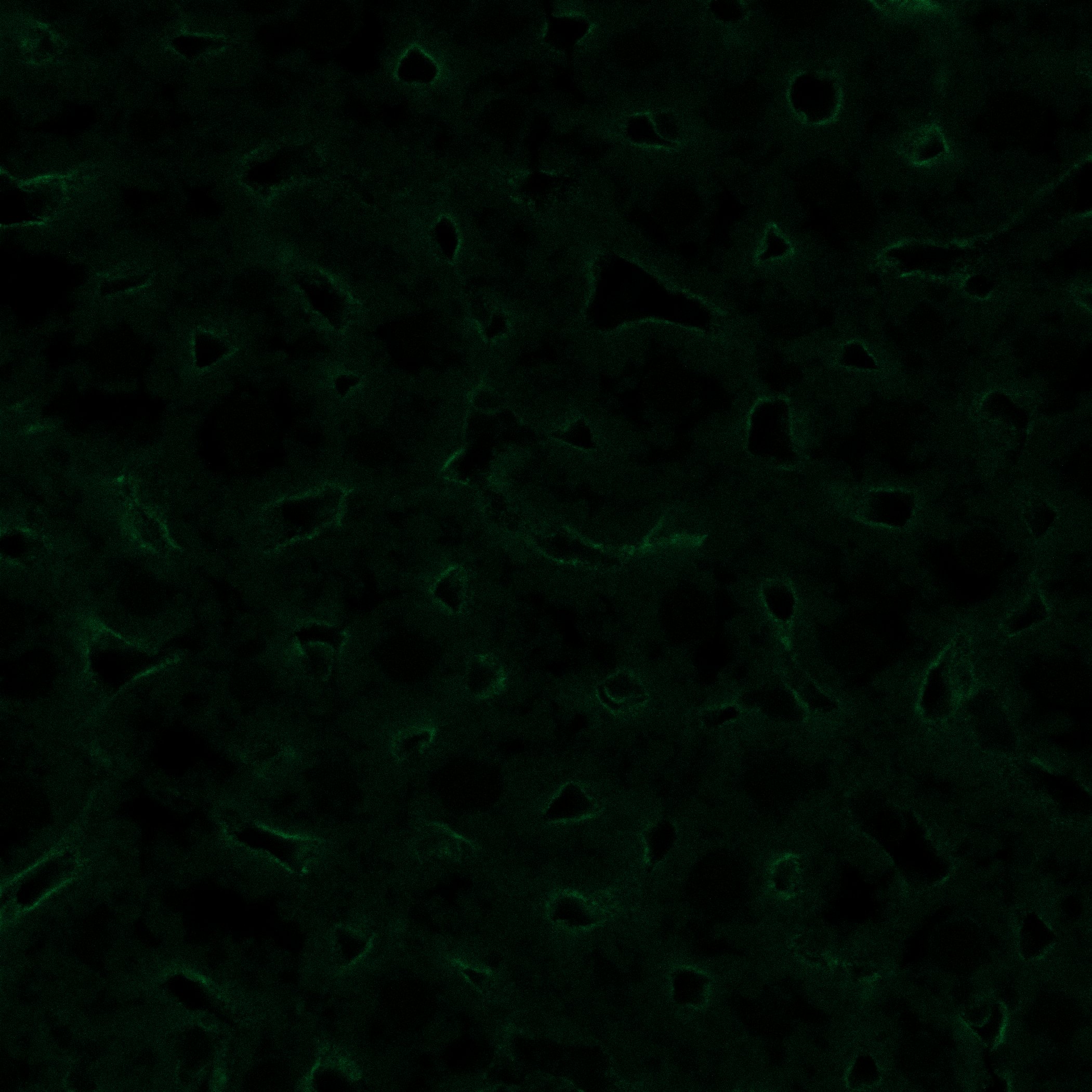

Supplement: Supplementary file 5 — Source data Fig. 1 [file 44319_2025_673_MOESM5_ESM.zip › Figure 1/1C/LSECs/CD146 staining_LSECs.jpg]

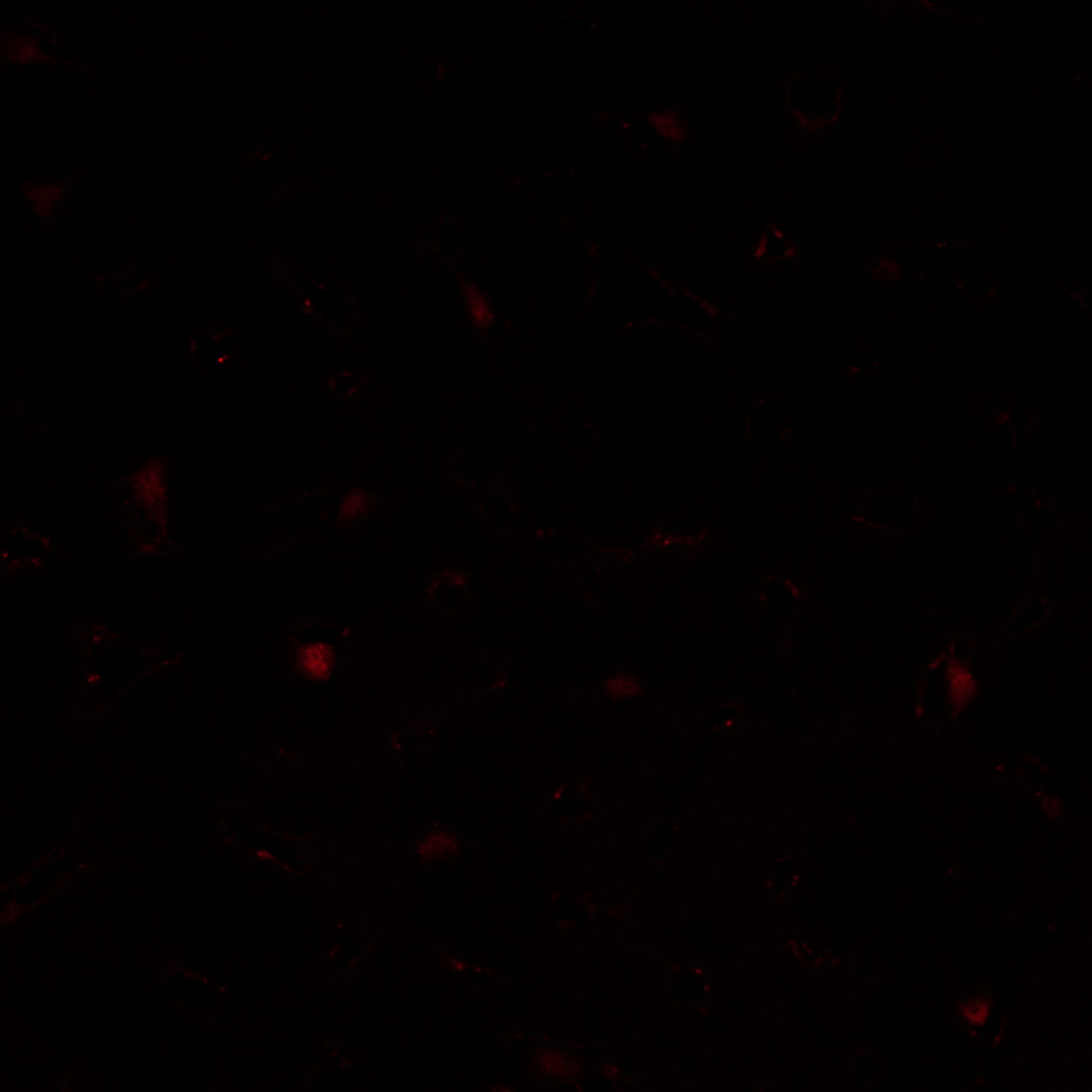

Supplement: Supplementary file 5 — Source data Fig. 1 [file 44319_2025_673_MOESM5_ESM.zip › Figure 1/1C/LSECs/Hgb_LSECs.jpg]

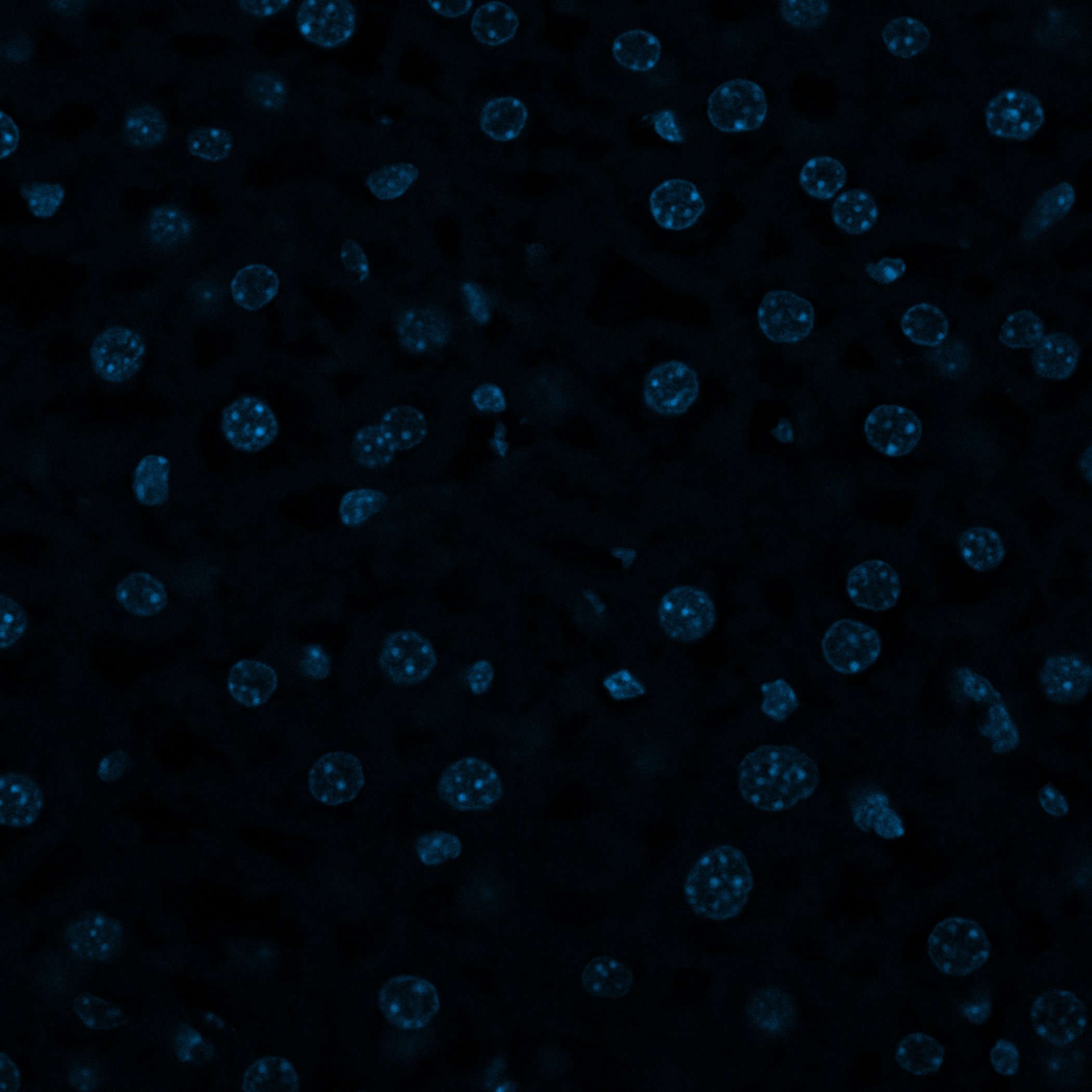

Supplement: Supplementary file 5 — Source data Fig. 1 [file 44319_2025_673_MOESM5_ESM.zip › Figure 1/1C/LSECs/Hoechst staining_LSECs.jpg]

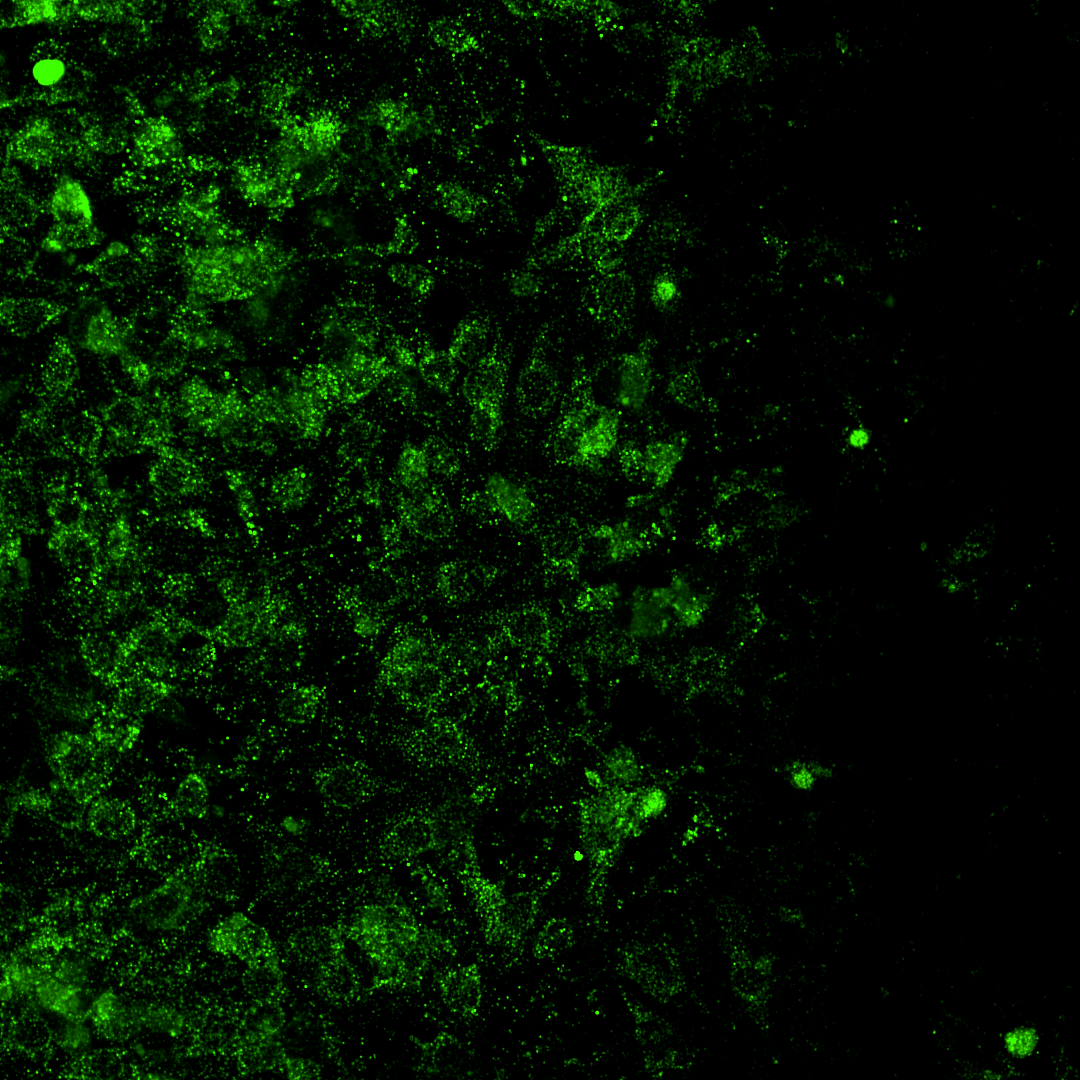

Supplement: Supplementary file 5 — Source data Fig. 1 [file 44319_2025_673_MOESM5_ESM.zip › Figure 1/1D/CTR/Ctrl-HB_A488_STAB2_1.png]

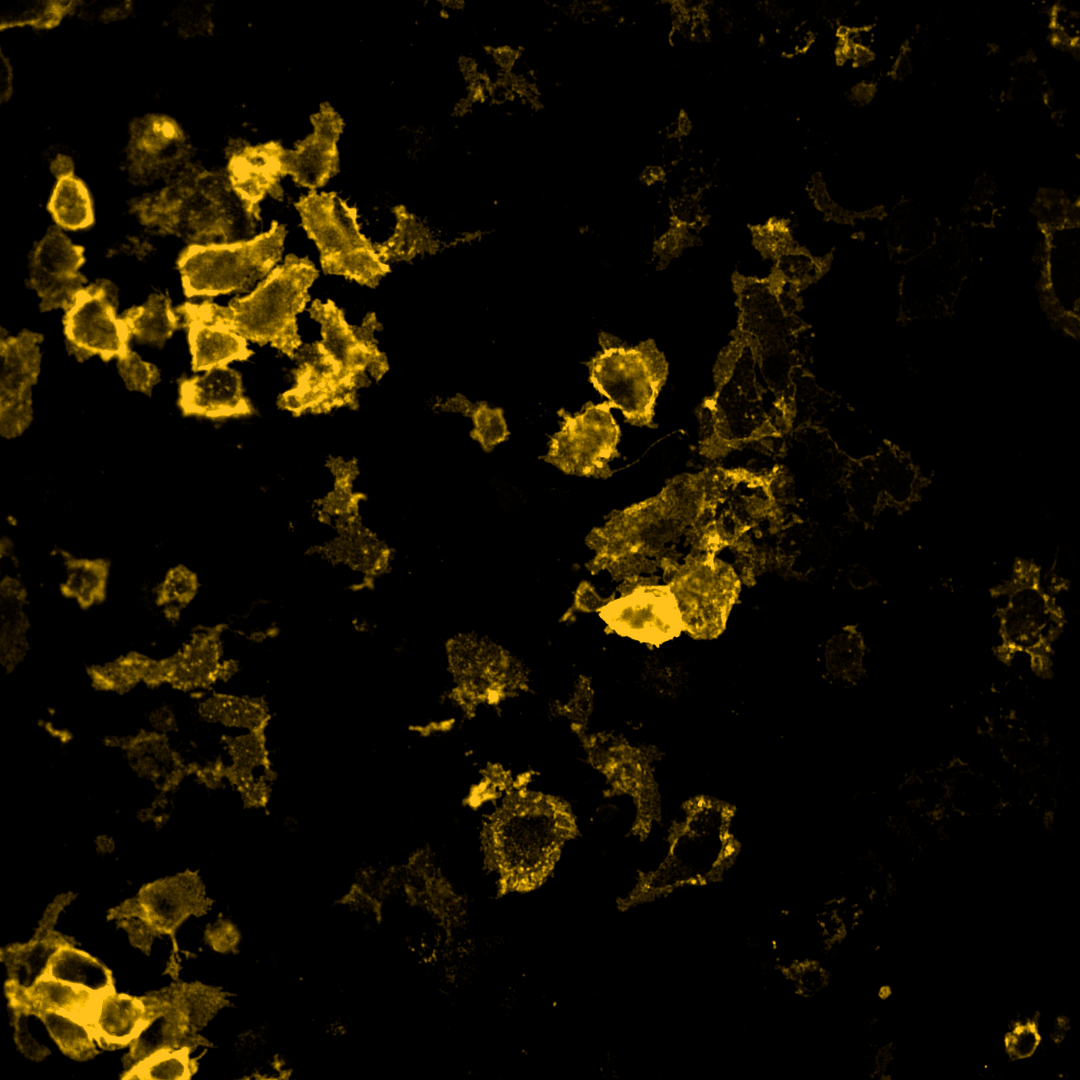

Supplement: Supplementary file 5 — Source data Fig. 1 [file 44319_2025_673_MOESM5_ESM.zip › Figure 1/1D/CTR/Ctrl-HB_A555_F480_1.png]

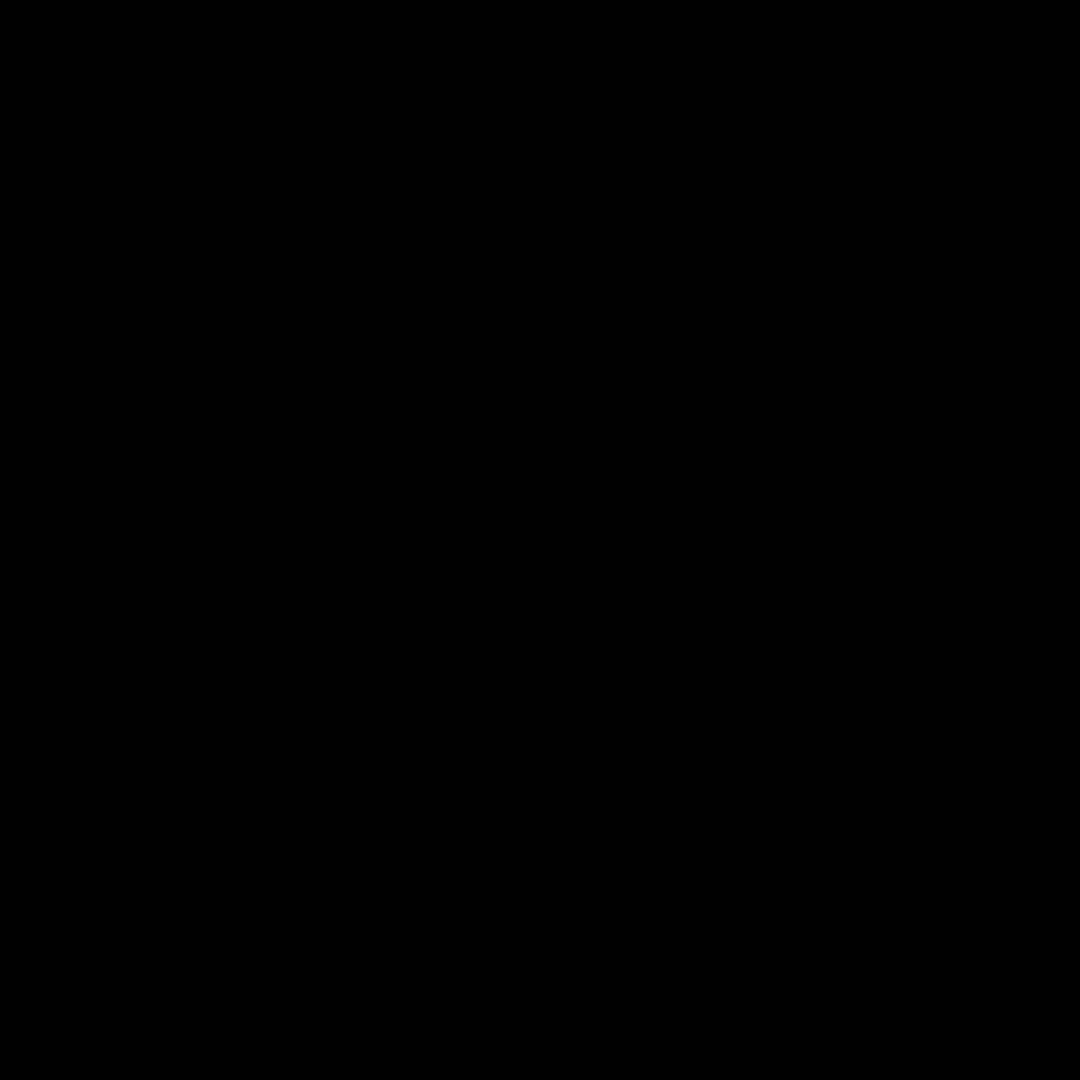

Supplement: Supplementary file 5 — Source data Fig. 1 [file 44319_2025_673_MOESM5_ESM.zip › Figure 1/1D/CTR/Ctrl-HB_A647_HB_1.png]

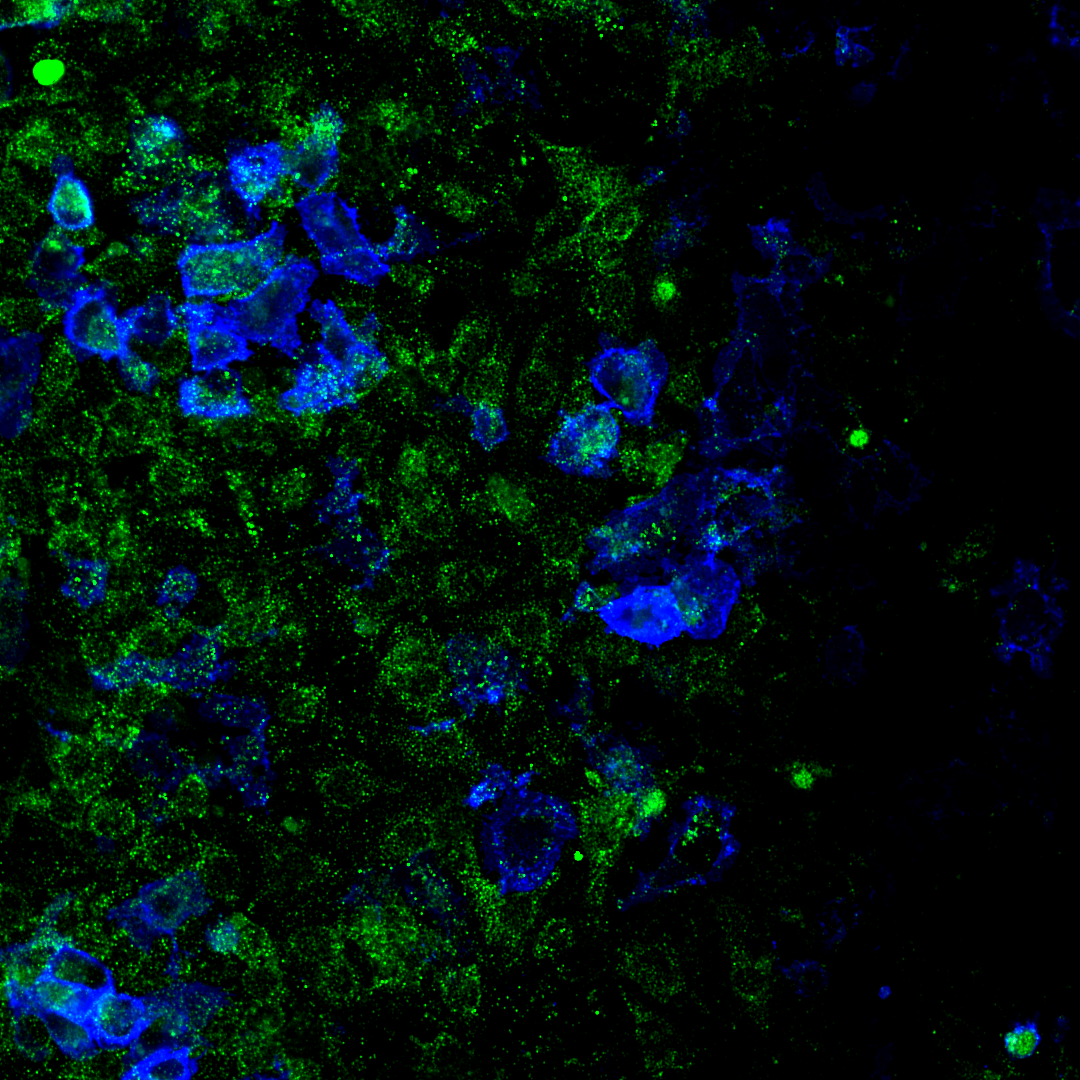

Supplement: Supplementary file 5 — Source data Fig. 1 [file 44319_2025_673_MOESM5_ESM.zip › Figure 1/1D/CTR/Ctrl-HB_M_1.tif]

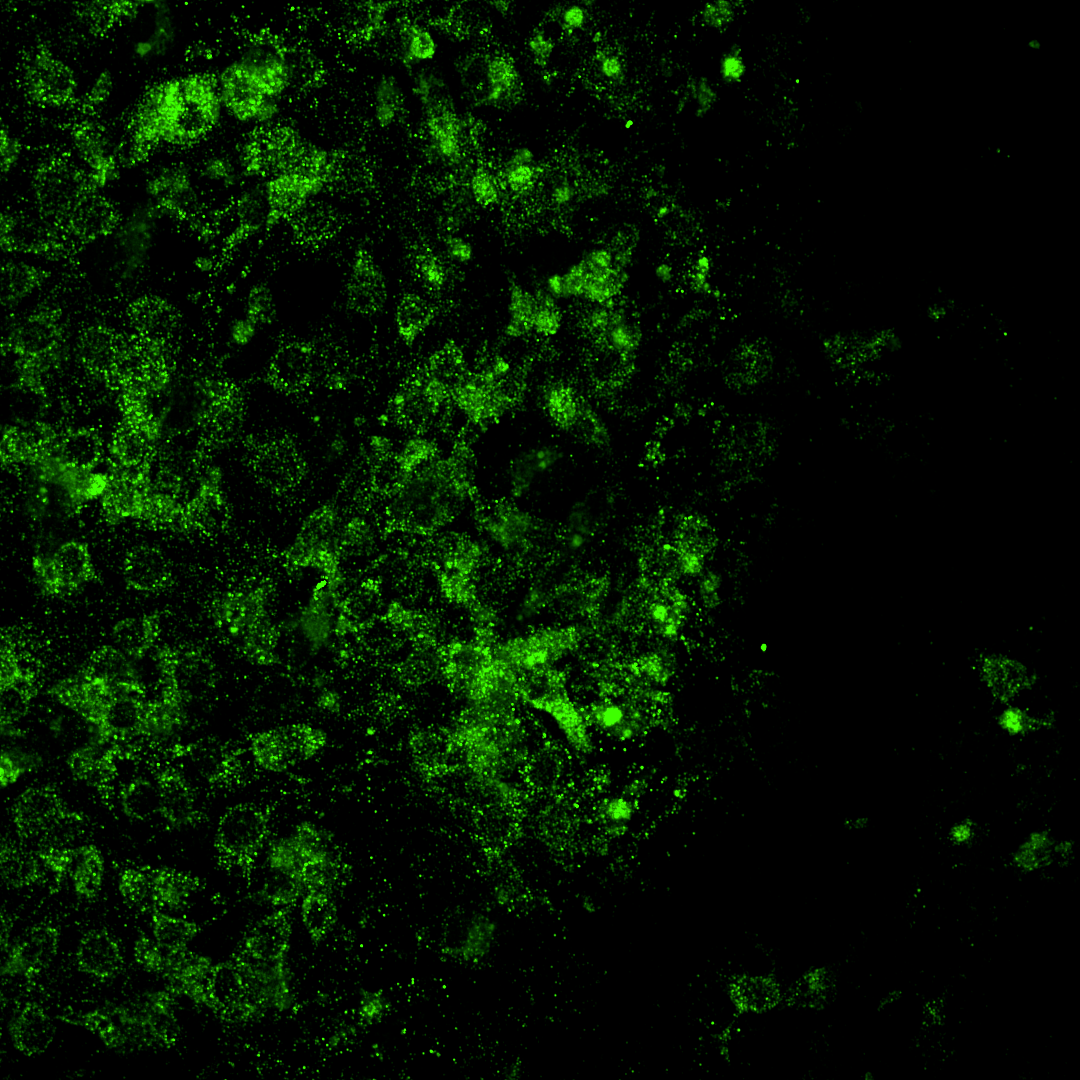

Supplement: Supplementary file 5 — Source data Fig. 1 [file 44319_2025_673_MOESM5_ESM.zip › Figure 1/1D/HGB/Ctrl+HB_A488_STAB2_2.png]

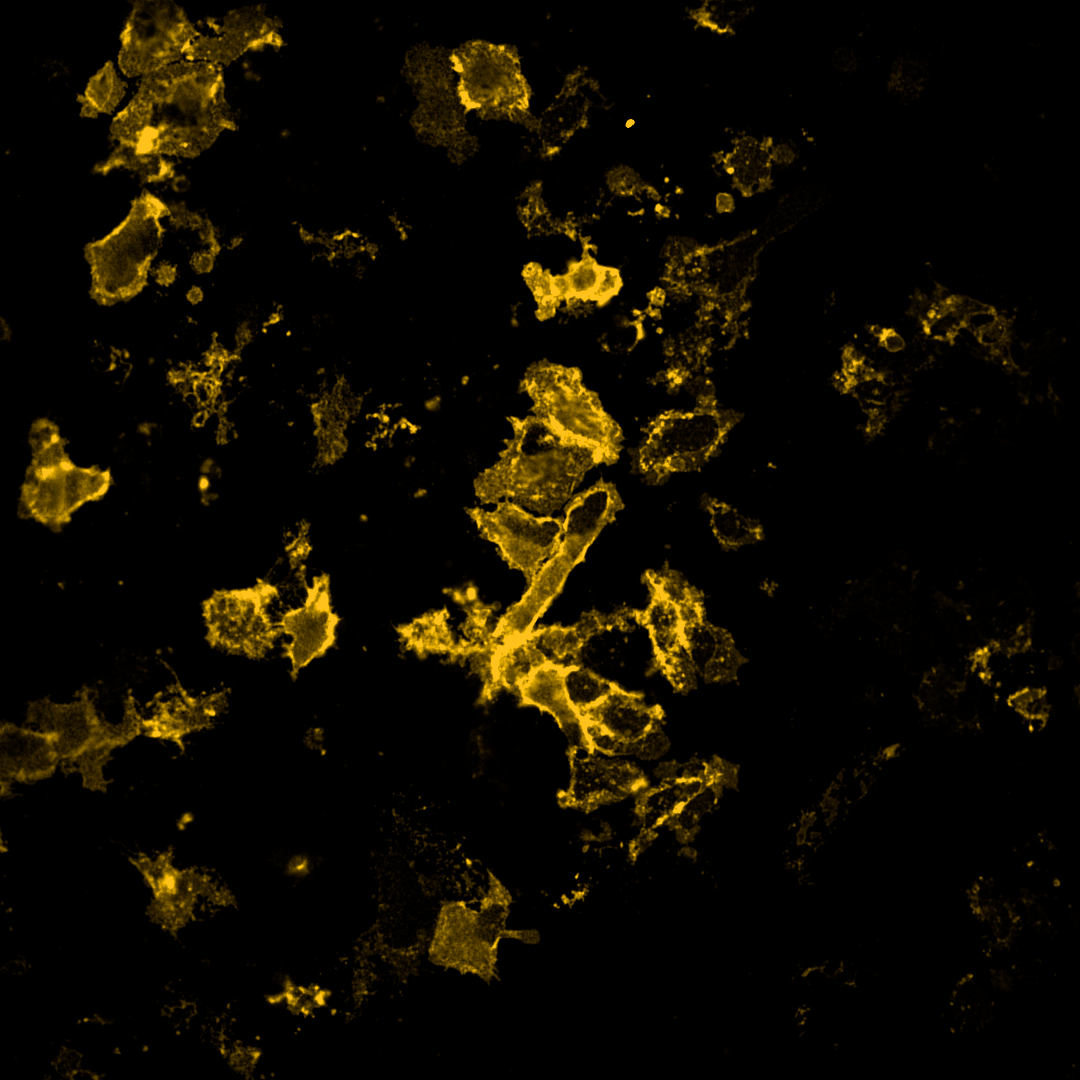

Supplement: Supplementary file 5 — Source data Fig. 1 [file 44319_2025_673_MOESM5_ESM.zip › Figure 1/1D/HGB/Ctrl+HB_A555_F480_2.png]

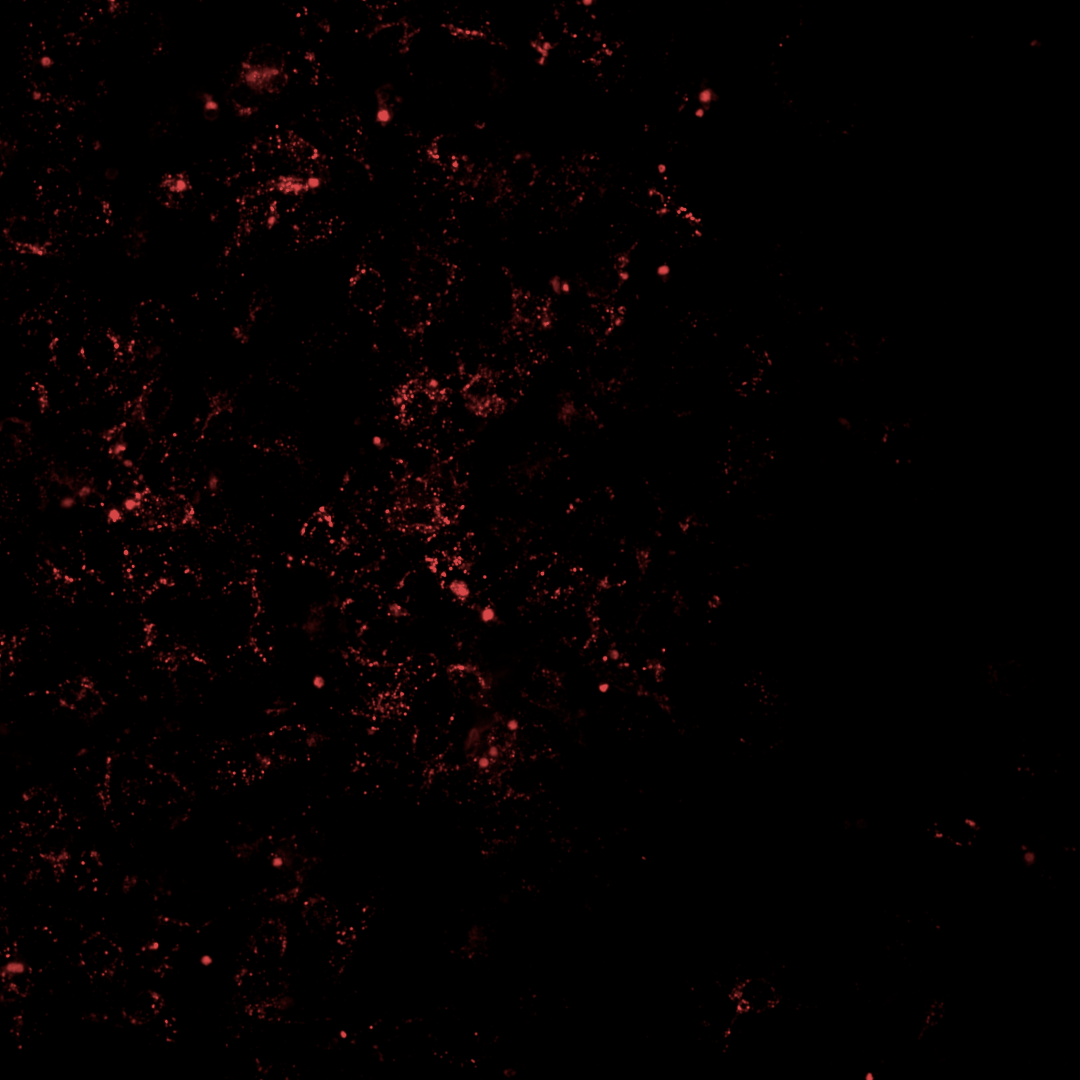

Supplement: Supplementary file 5 — Source data Fig. 1 [file 44319_2025_673_MOESM5_ESM.zip › Figure 1/1D/HGB/Ctrl+HB_A647_HB_2.png]

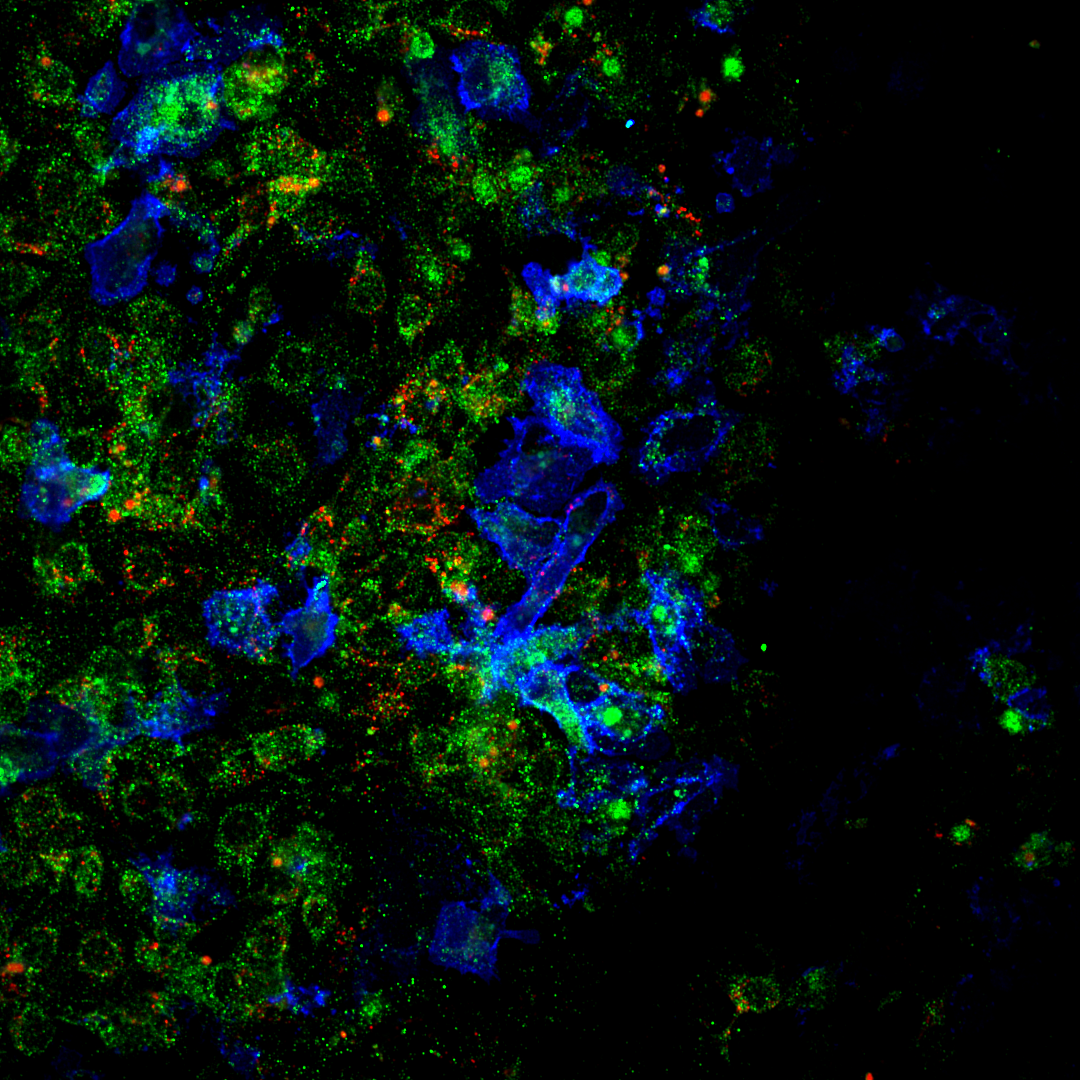

Supplement: Supplementary file 5 — Source data Fig. 1 [file 44319_2025_673_MOESM5_ESM.zip › Figure 1/1D/HGB/Ctrl+HB_M_2.tif]

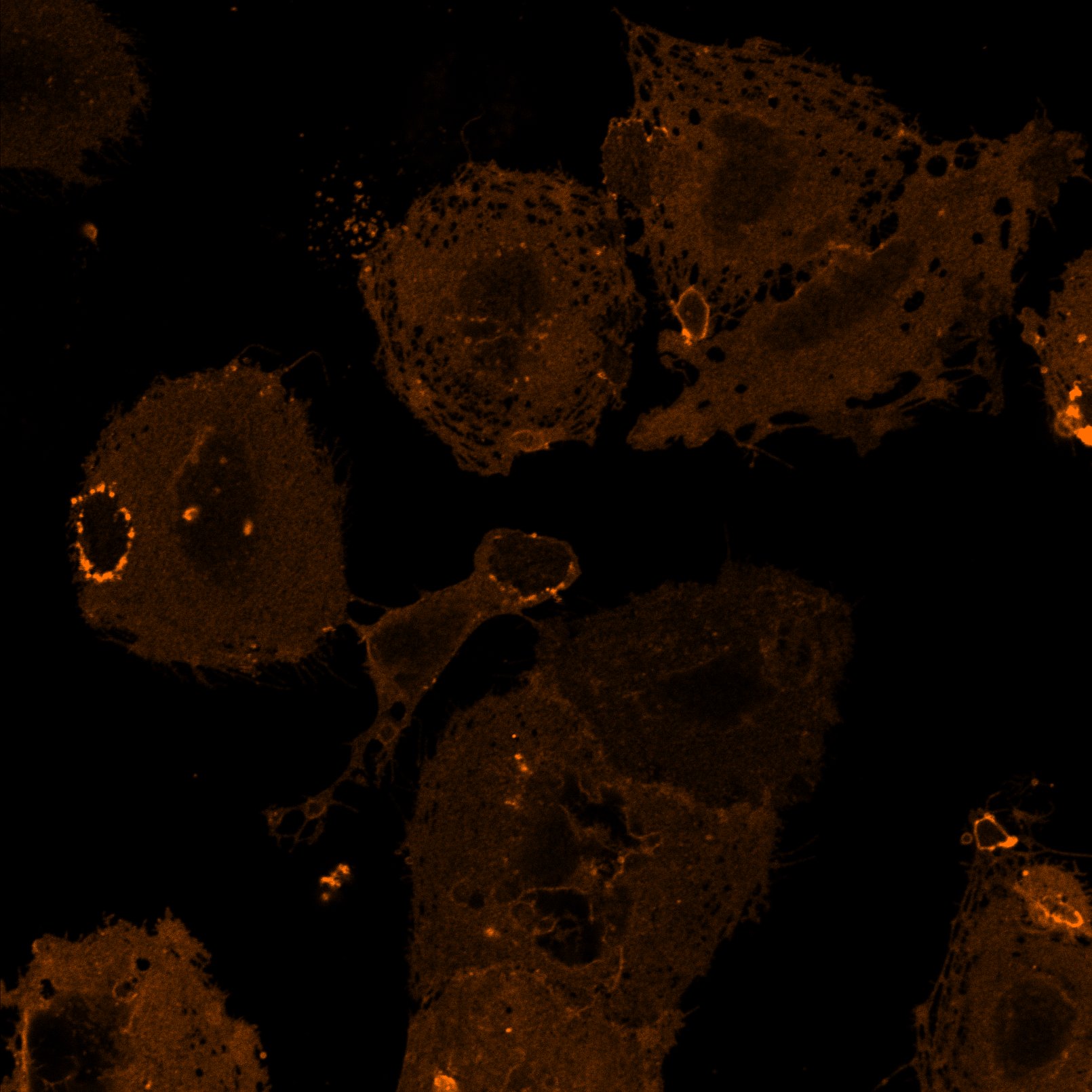

Supplement: Supplementary file 5 — Source data Fig. 1 [file 44319_2025_673_MOESM5_ESM.zip › Figure 1/1F/CD32B/CD32B staining.jpg]

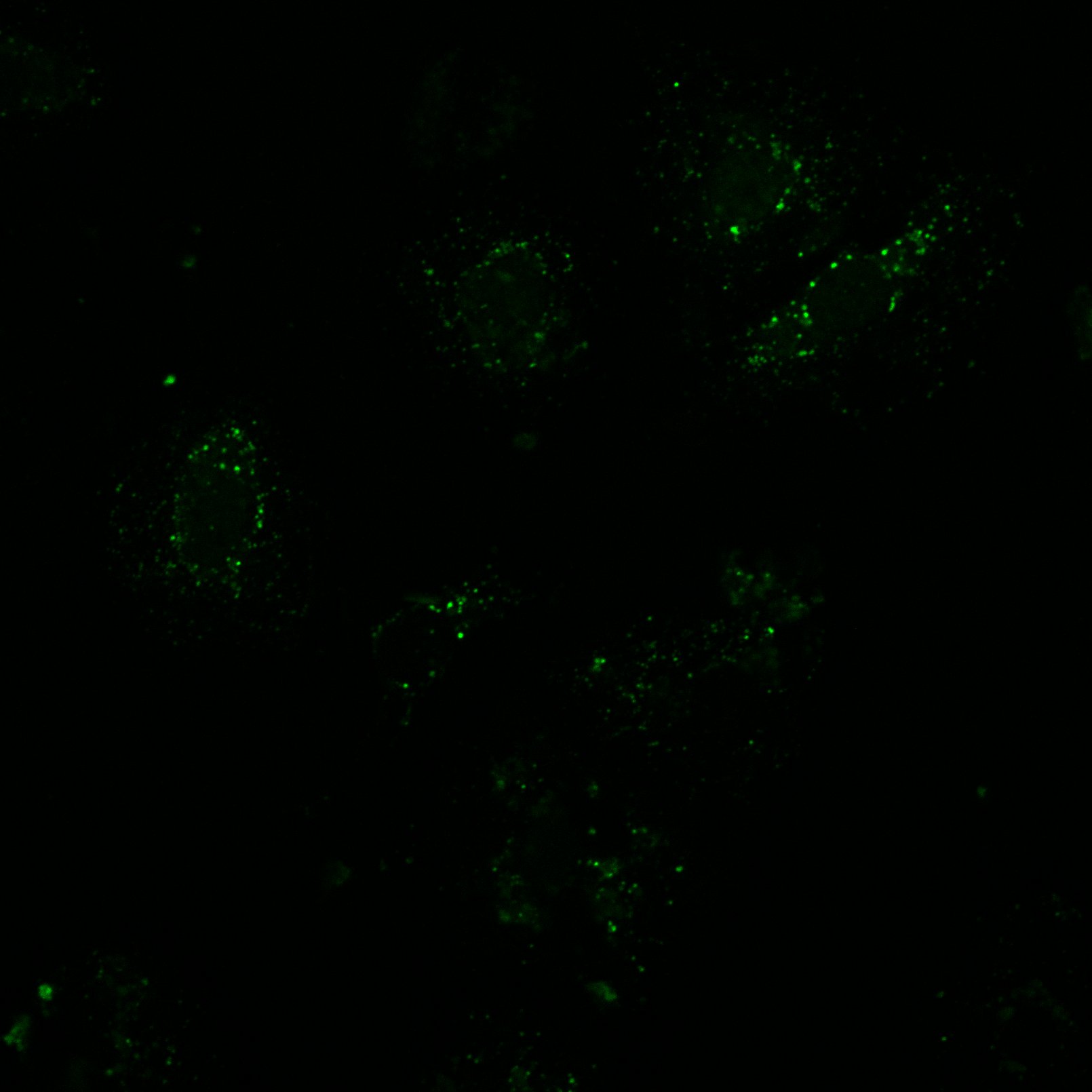

Supplement: Supplementary file 5 — Source data Fig. 1 [file 44319_2025_673_MOESM5_ESM.zip › Figure 1/1F/CD32B/Hgb.jpg]

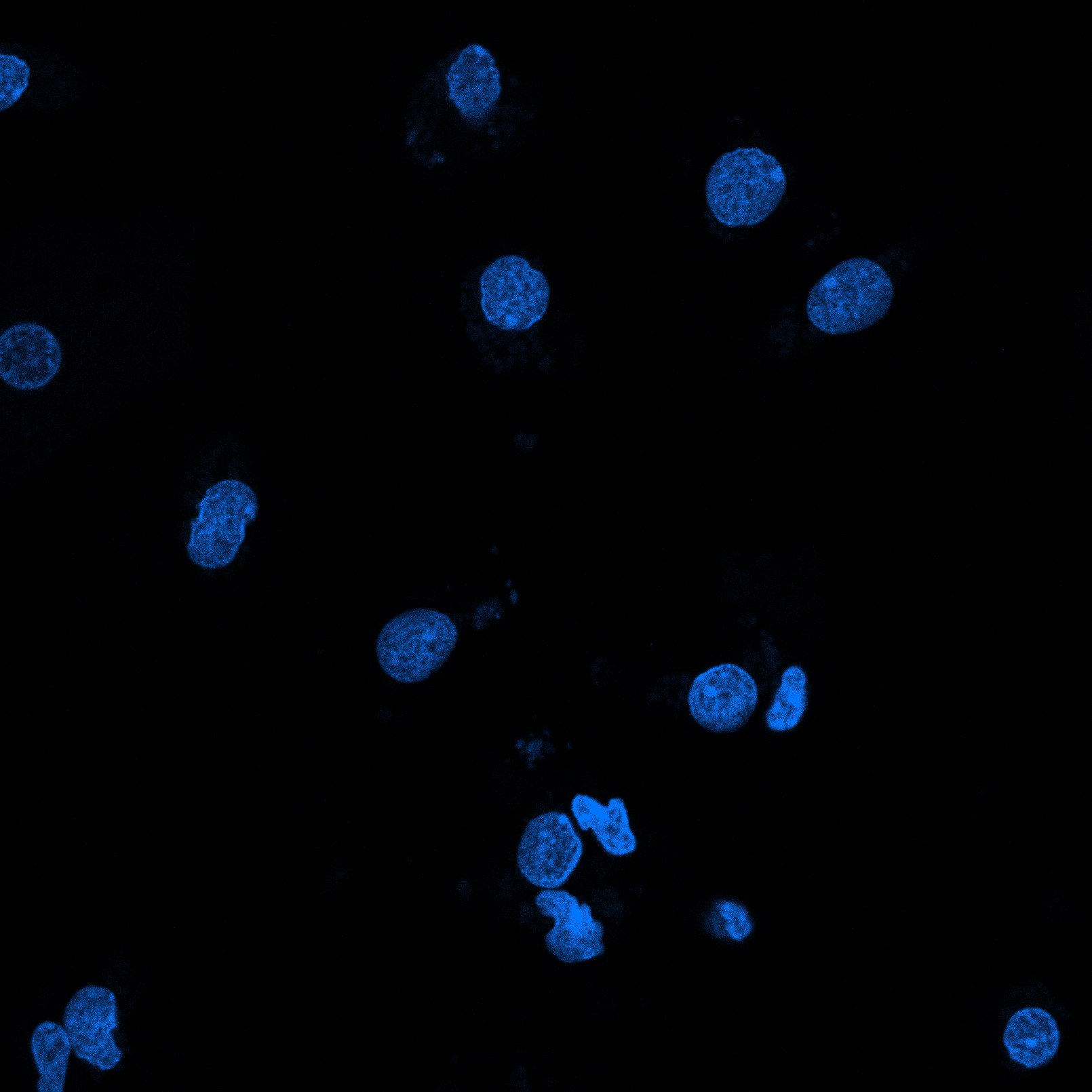

Supplement: Supplementary file 5 — Source data Fig. 1 [file 44319_2025_673_MOESM5_ESM.zip › Figure 1/1F/CD32B/Hoechst staining.jpg]

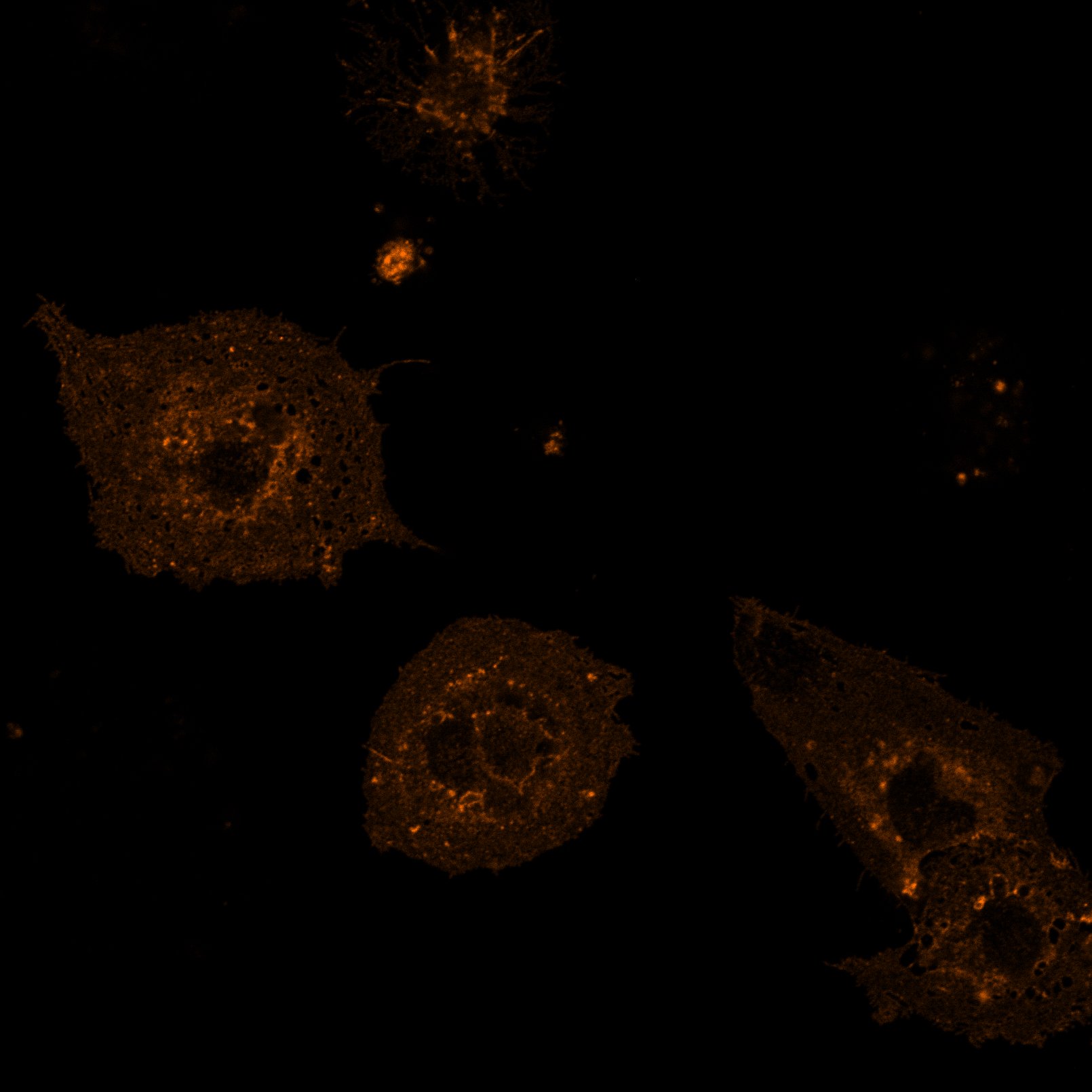

Supplement: Supplementary file 5 — Source data Fig. 1 [file 44319_2025_673_MOESM5_ESM.zip › Figure 1/1F/CD36/CD36.jpg]

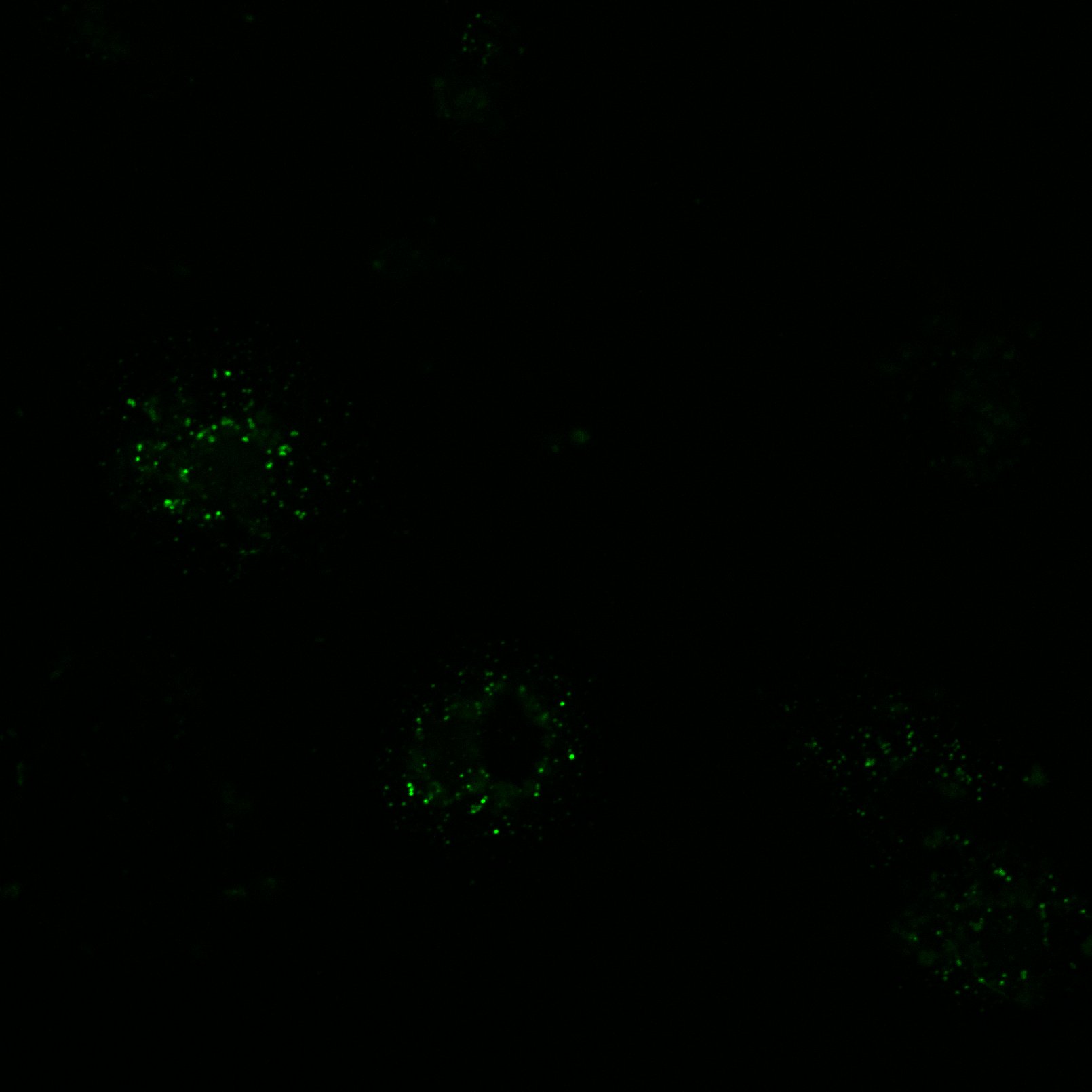

Supplement: Supplementary file 5 — Source data Fig. 1 [file 44319_2025_673_MOESM5_ESM.zip › Figure 1/1F/CD36/Hgb.jpg]

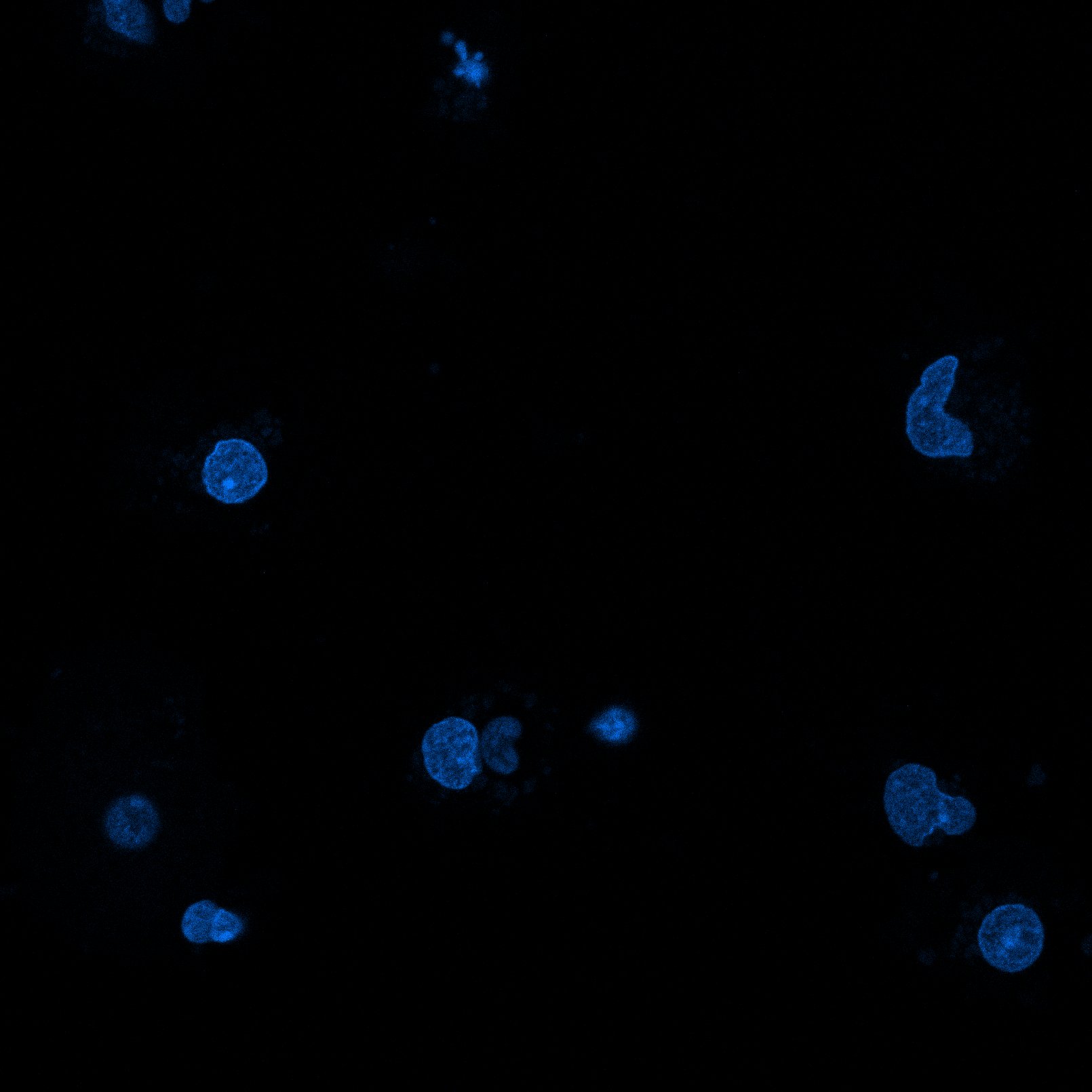

Supplement: Supplementary file 5 — Source data Fig. 1 [file 44319_2025_673_MOESM5_ESM.zip › Figure 1/1F/CD36/Hoechst staining.jpg]

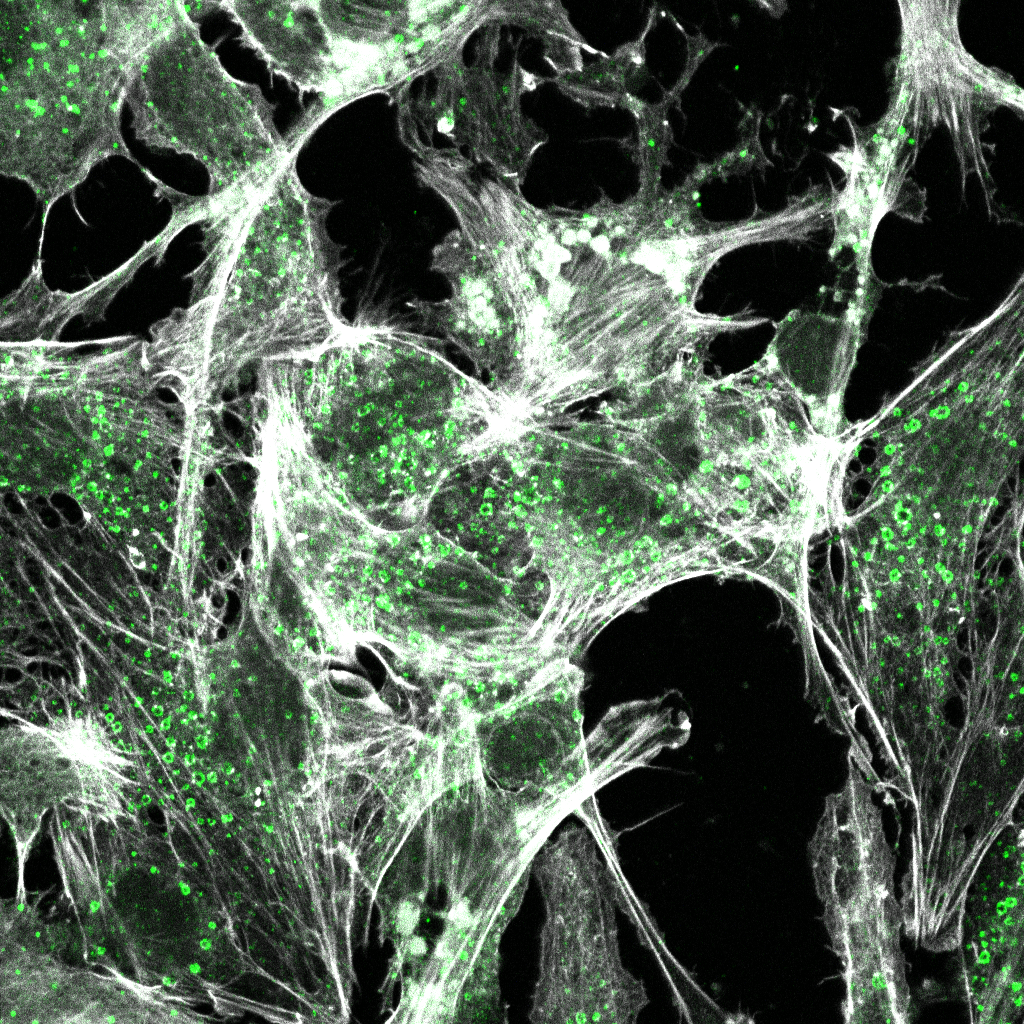

Supplement: Supplementary file 6 — Source data Fig. 2 [file 44319_2025_673_MOESM6_ESM.zip › Figure 2/2C/control_merged.tif]

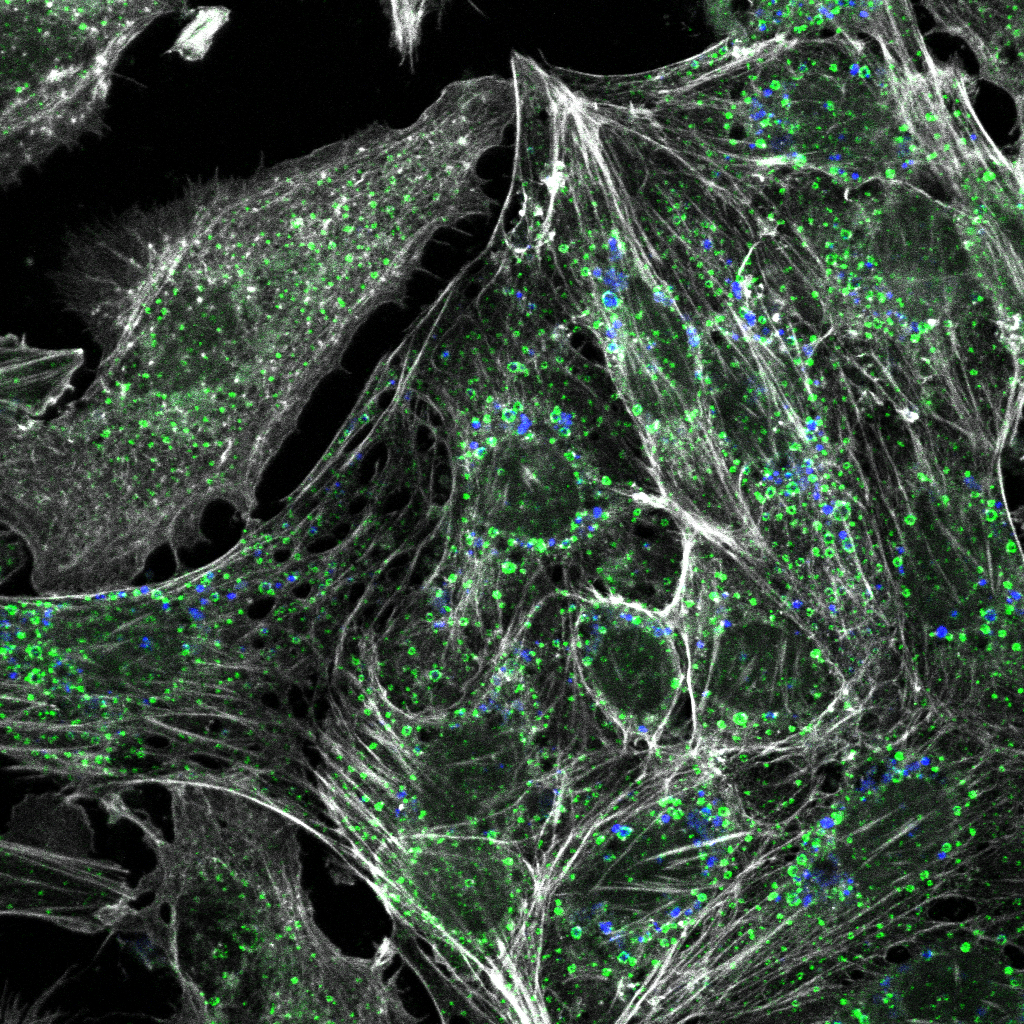

Supplement: Supplementary file 6 — Source data Fig. 2 [file 44319_2025_673_MOESM6_ESM.zip › Figure 2/2C/Hb 1h_merged.tif]

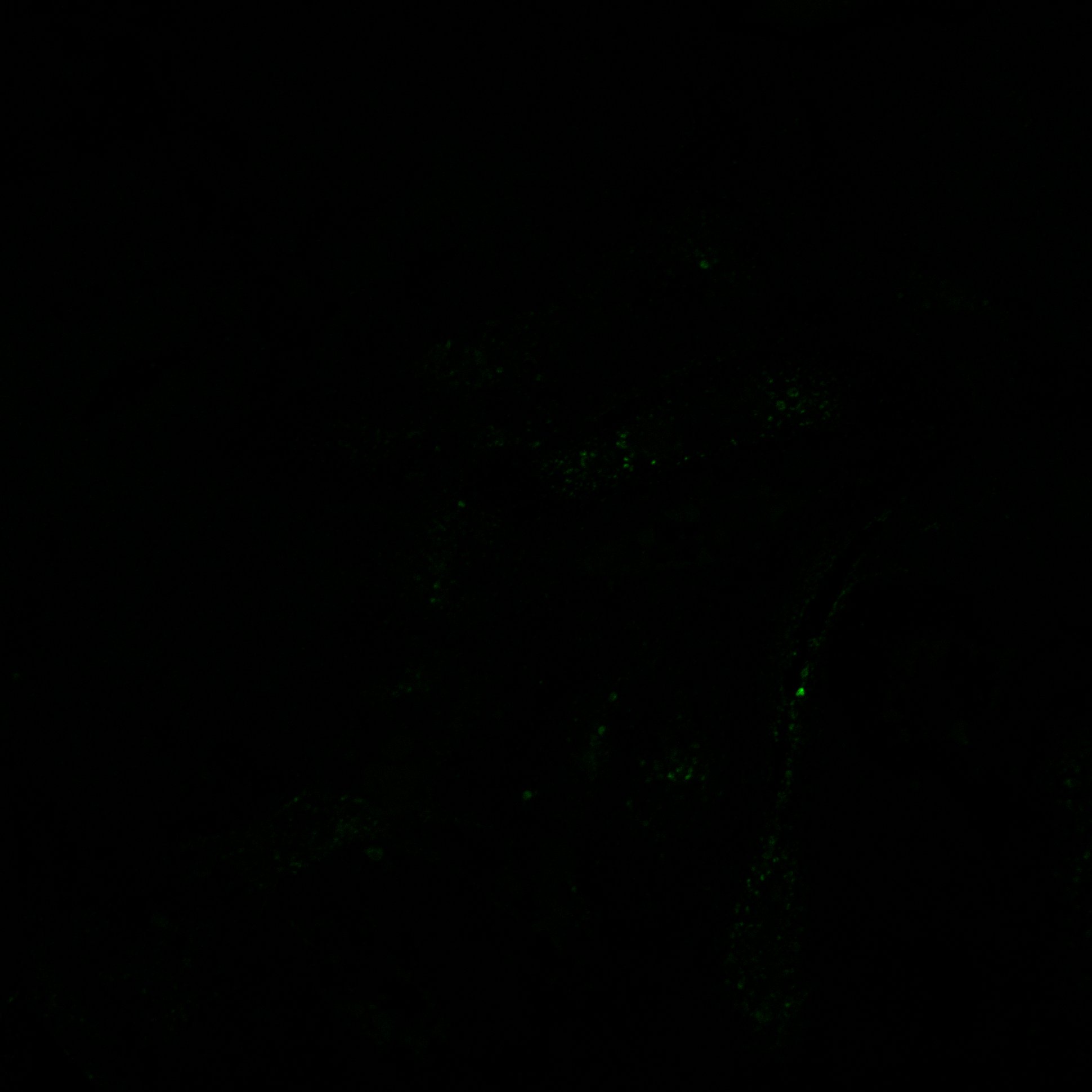

Supplement: Supplementary file 6 — Source data Fig. 2 [file 44319_2025_673_MOESM6_ESM.zip › Figure 2/2D/Dextran.jpg]

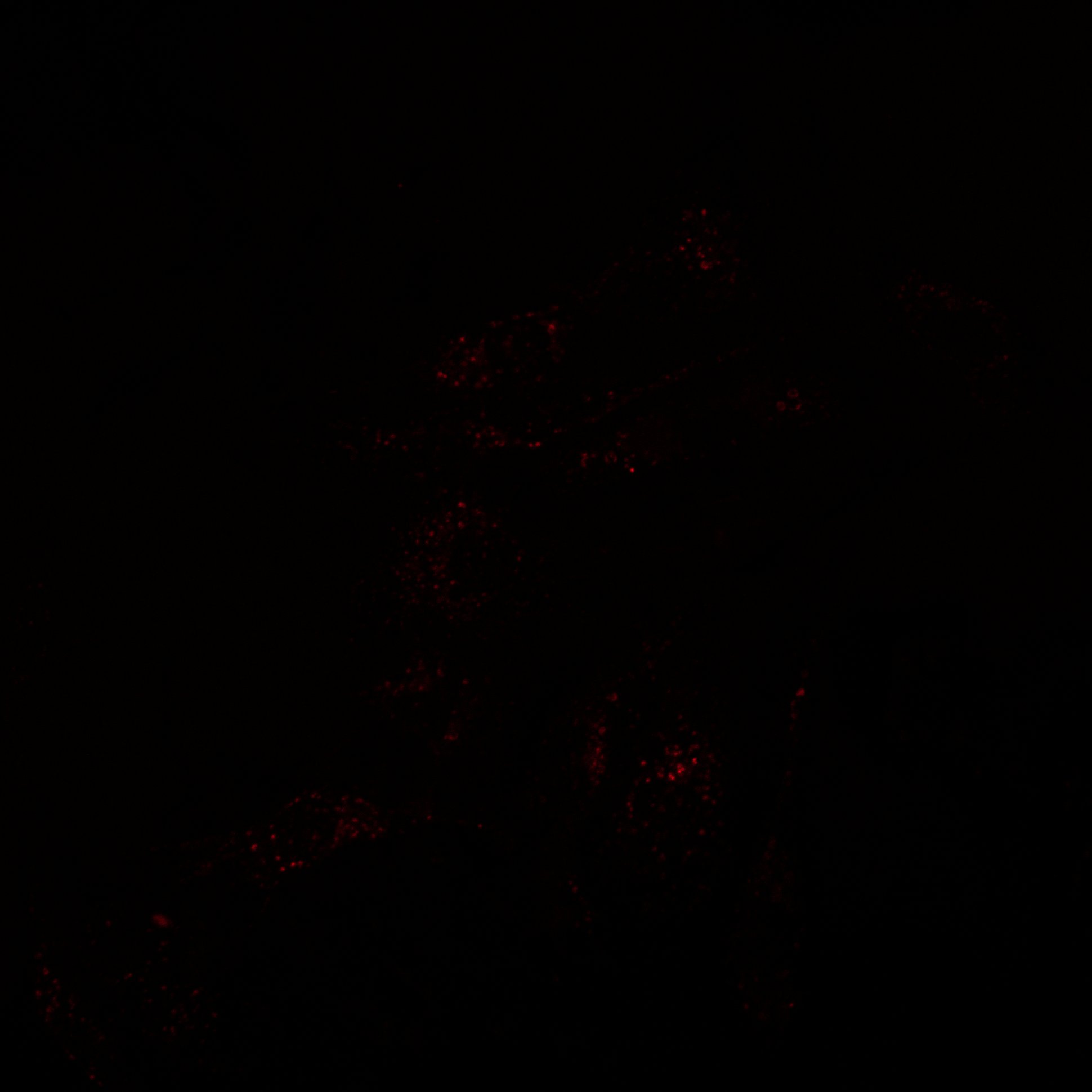

Supplement: Supplementary file 6 — Source data Fig. 2 [file 44319_2025_673_MOESM6_ESM.zip › Figure 2/2D/Hgb.jpg]

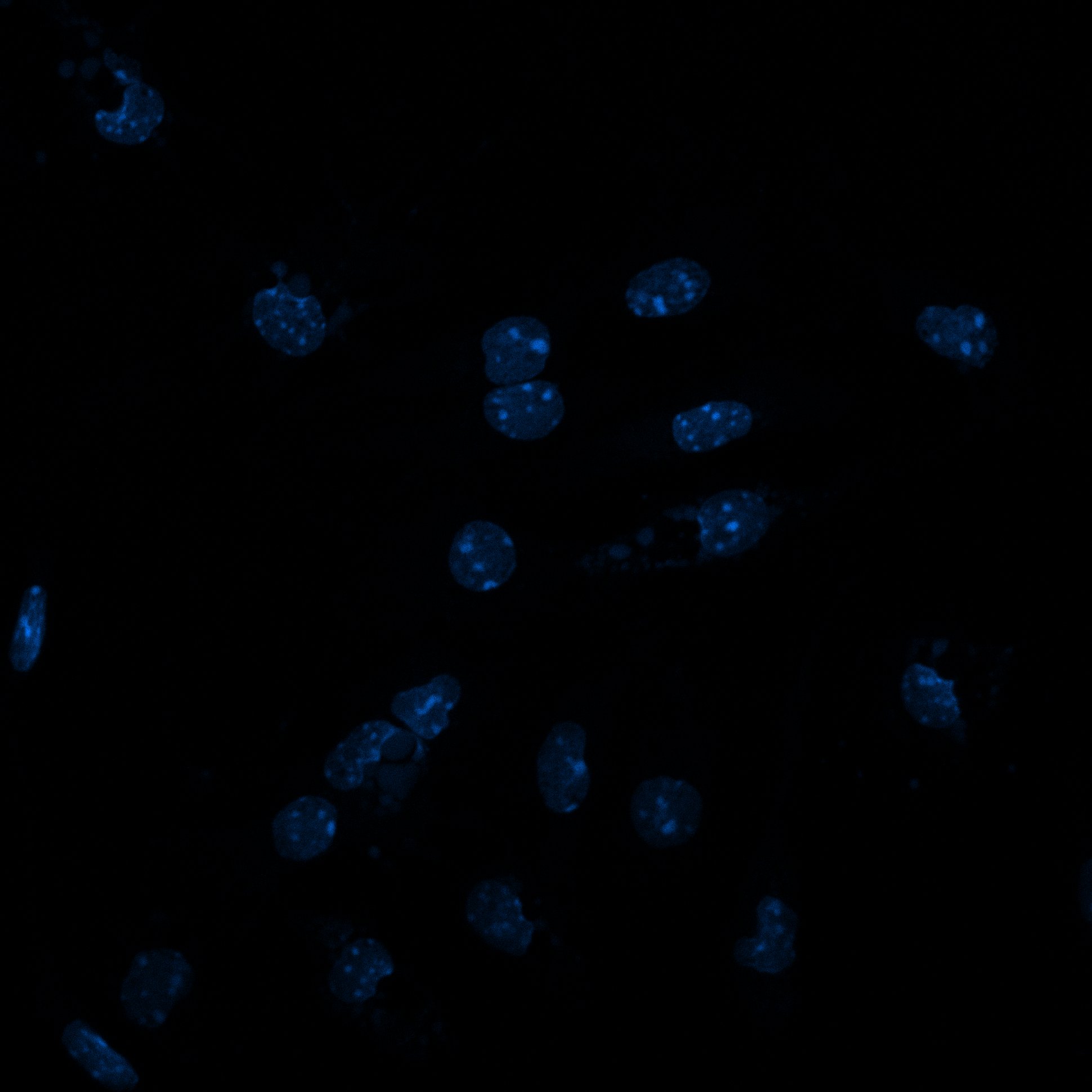

Supplement: Supplementary file 6 — Source data Fig. 2 [file 44319_2025_673_MOESM6_ESM.zip › Figure 2/2D/Hoechst staining.jpg]

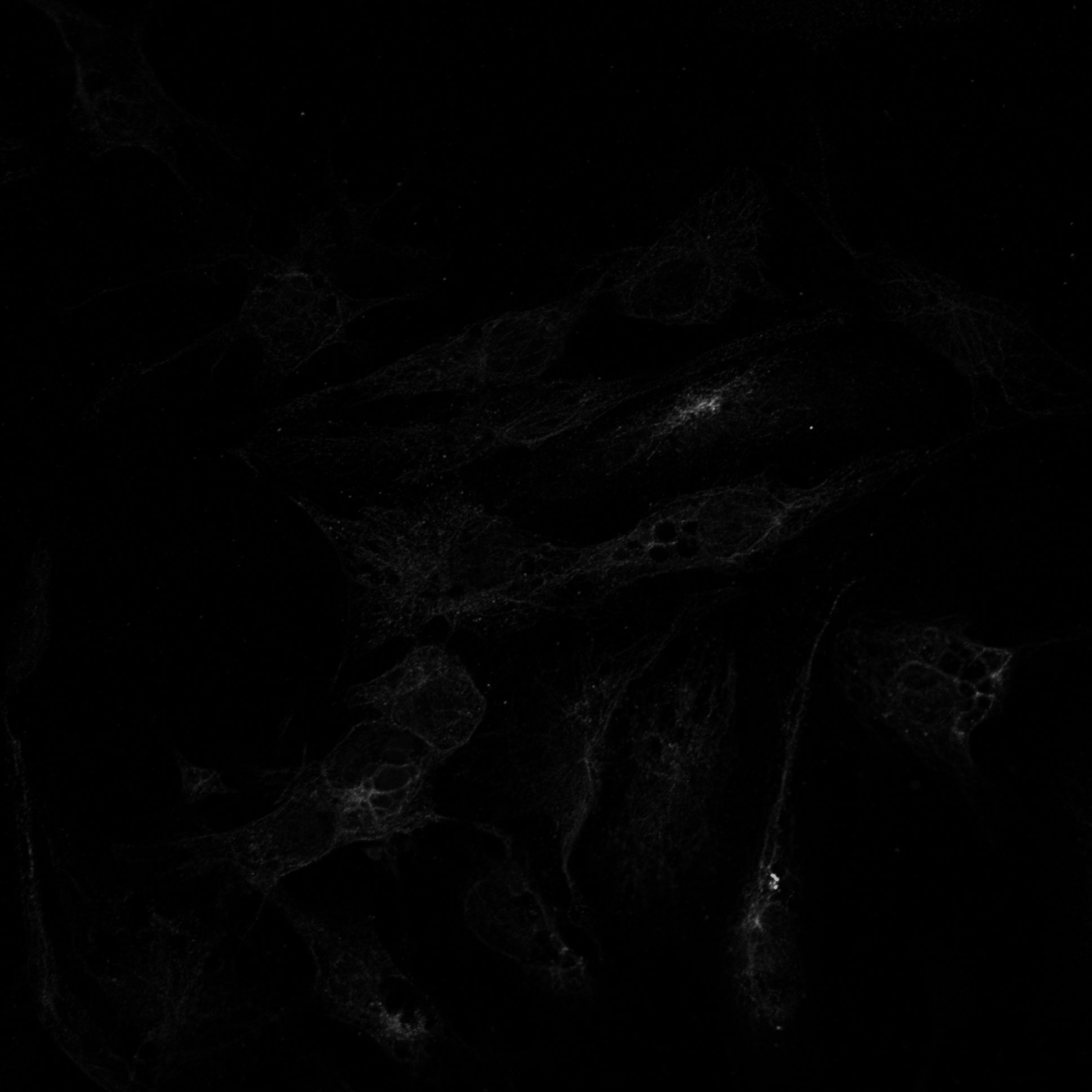

Supplement: Supplementary file 6 — Source data Fig. 2 [file 44319_2025_673_MOESM6_ESM.zip › Figure 2/2D/Stab2 staining.jpg]

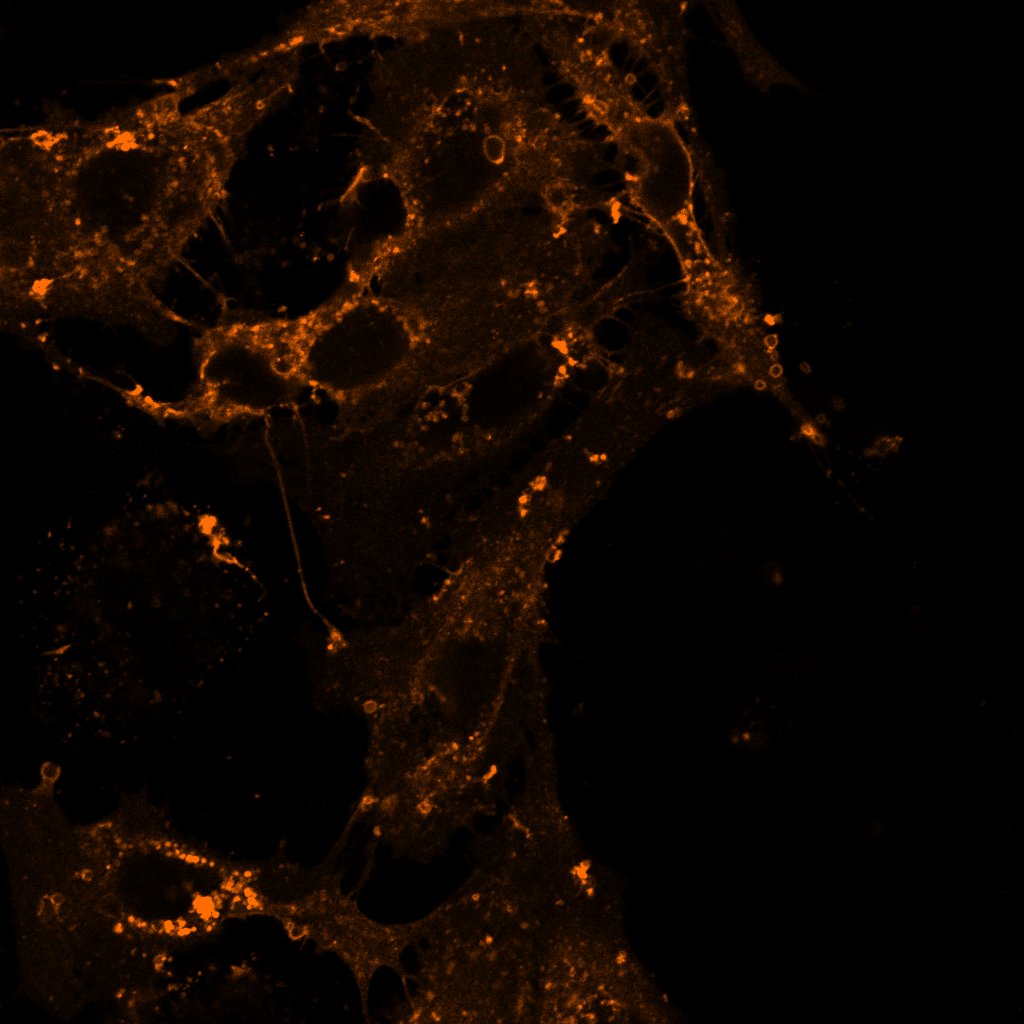

Supplement: Supplementary file 6 — Source data Fig. 2 [file 44319_2025_673_MOESM6_ESM.zip › Figure 2/2E/CD36 staining.jpg]

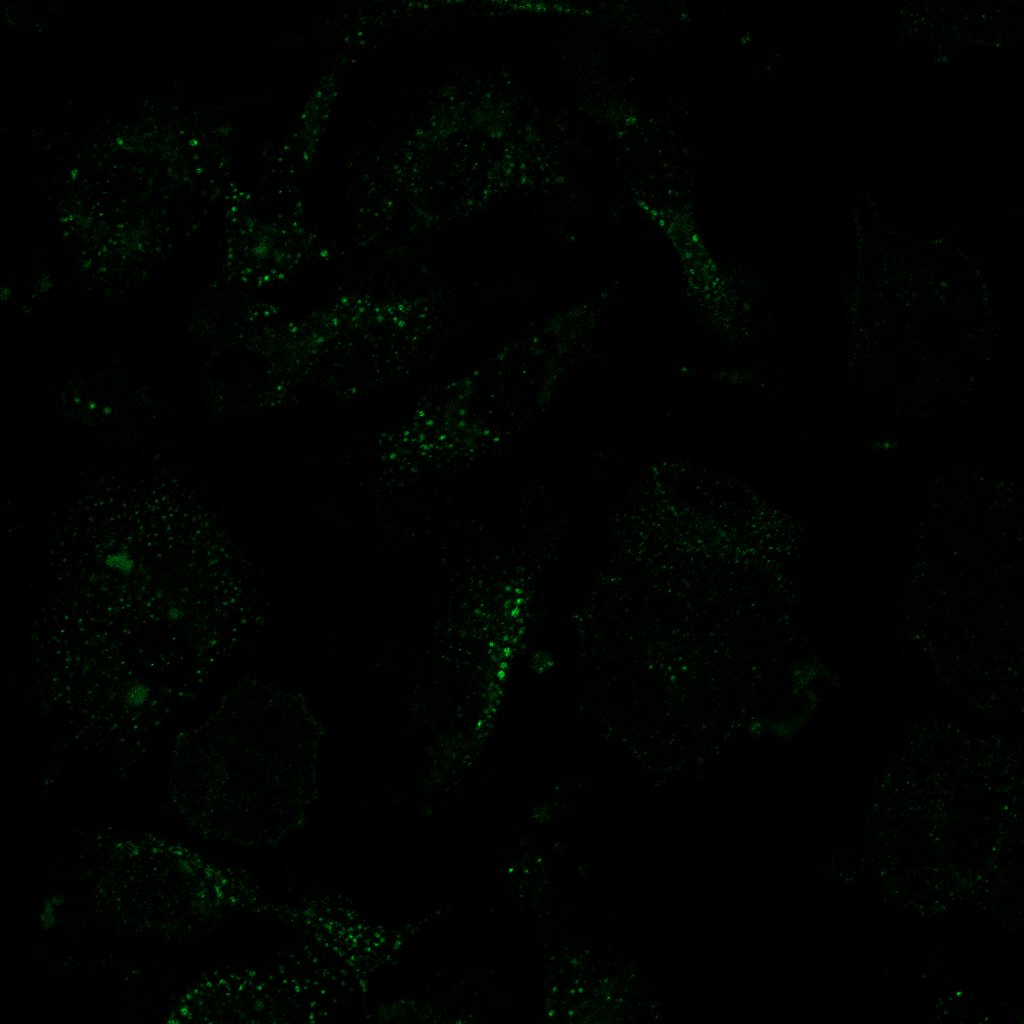

Supplement: Supplementary file 6 — Source data Fig. 2 [file 44319_2025_673_MOESM6_ESM.zip › Figure 2/2E/EEA1 staining.jpg]

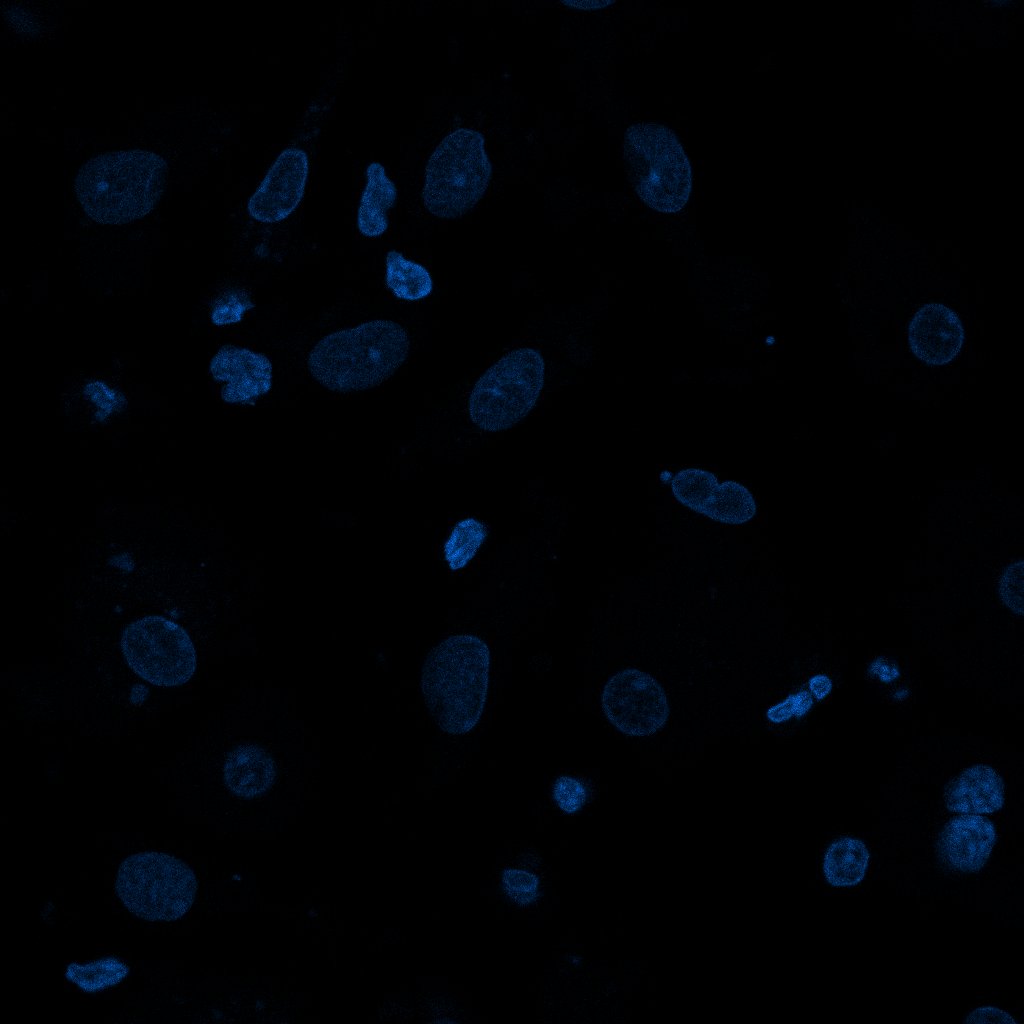

Supplement: Supplementary file 6 — Source data Fig. 2 [file 44319_2025_673_MOESM6_ESM.zip › Figure 2/2E/Hoechst staining.jpg]

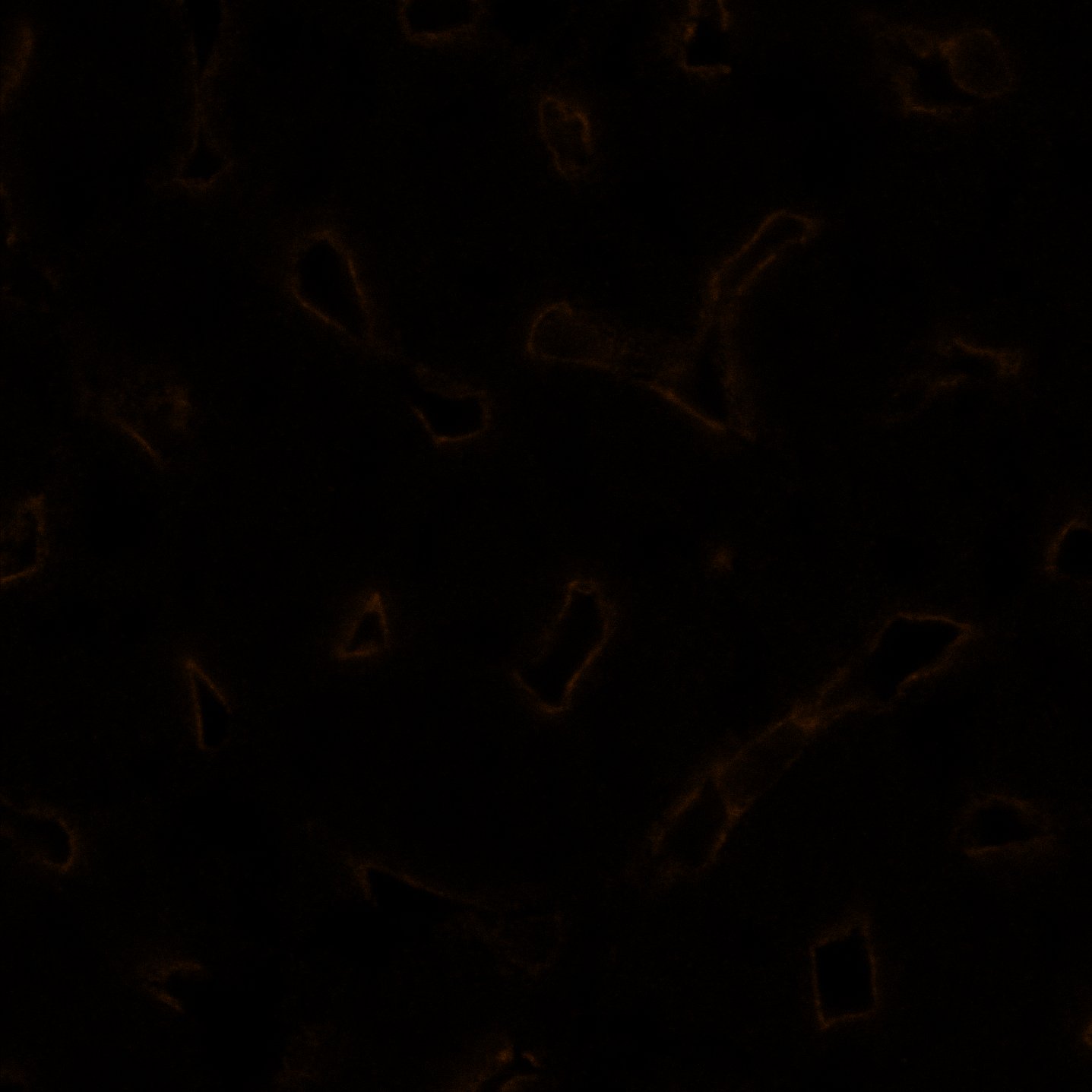

Supplement: Supplementary file 11 — Source data Fig. 4A-H [file 44319_2025_673_MOESM11_ESM.zip › 4A/FPN/CD146 staining.jpg]

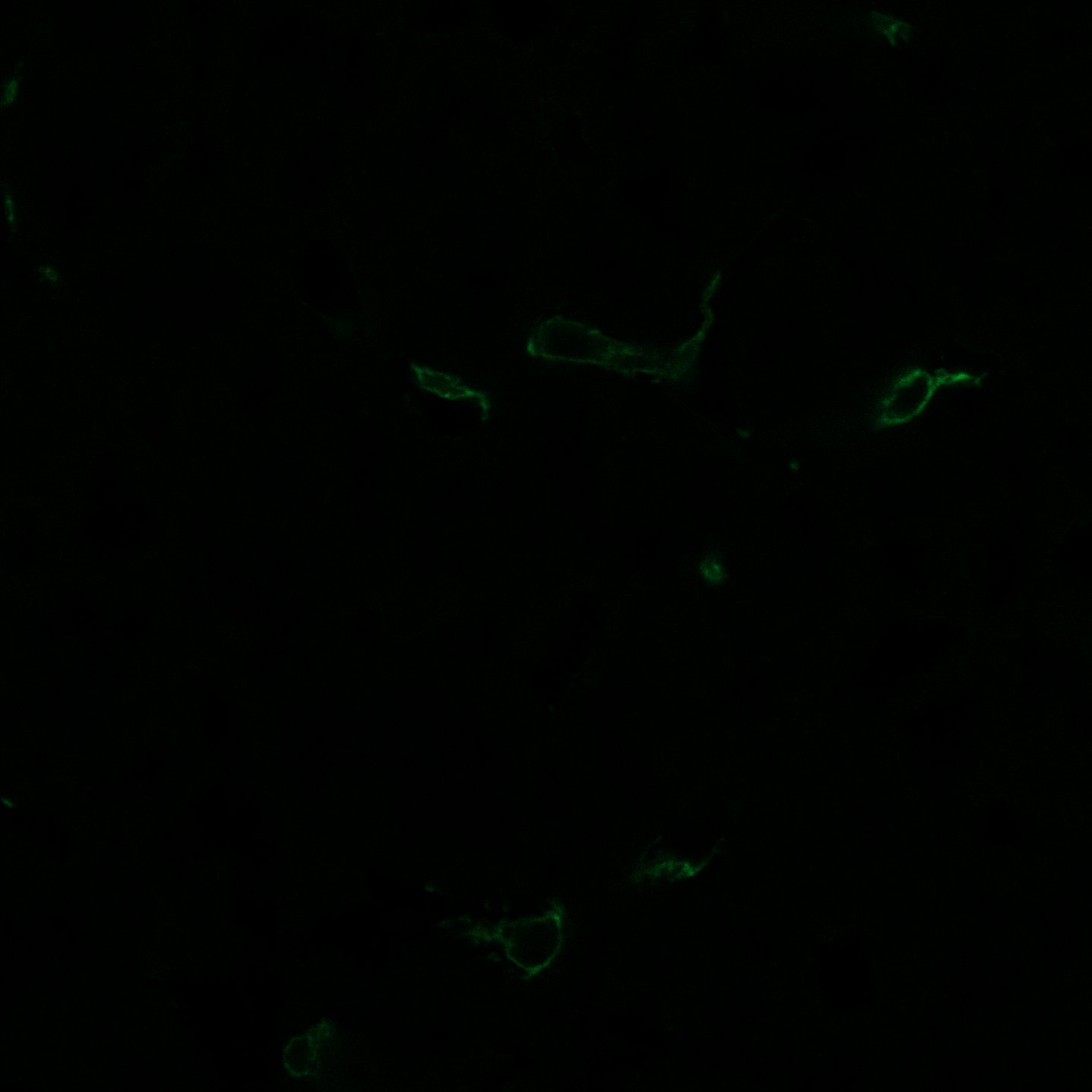

Supplement: Supplementary file 11 — Source data Fig. 4A-H [file 44319_2025_673_MOESM11_ESM.zip › 4A/FPN/F480 staining.jpg]

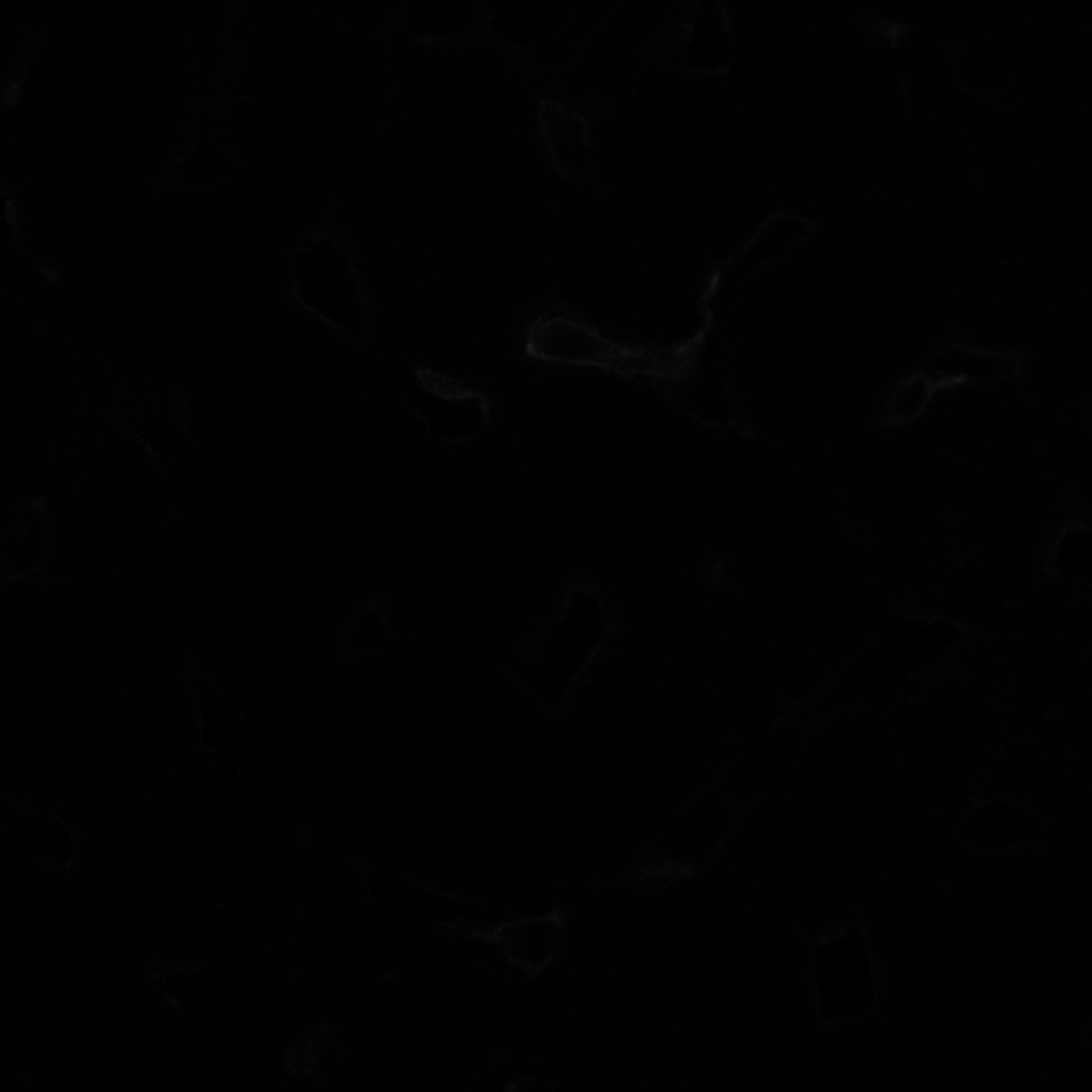

Supplement: Supplementary file 11 — Source data Fig. 4A-H [file 44319_2025_673_MOESM11_ESM.zip › 4A/FPN/FPN staining.jpg]

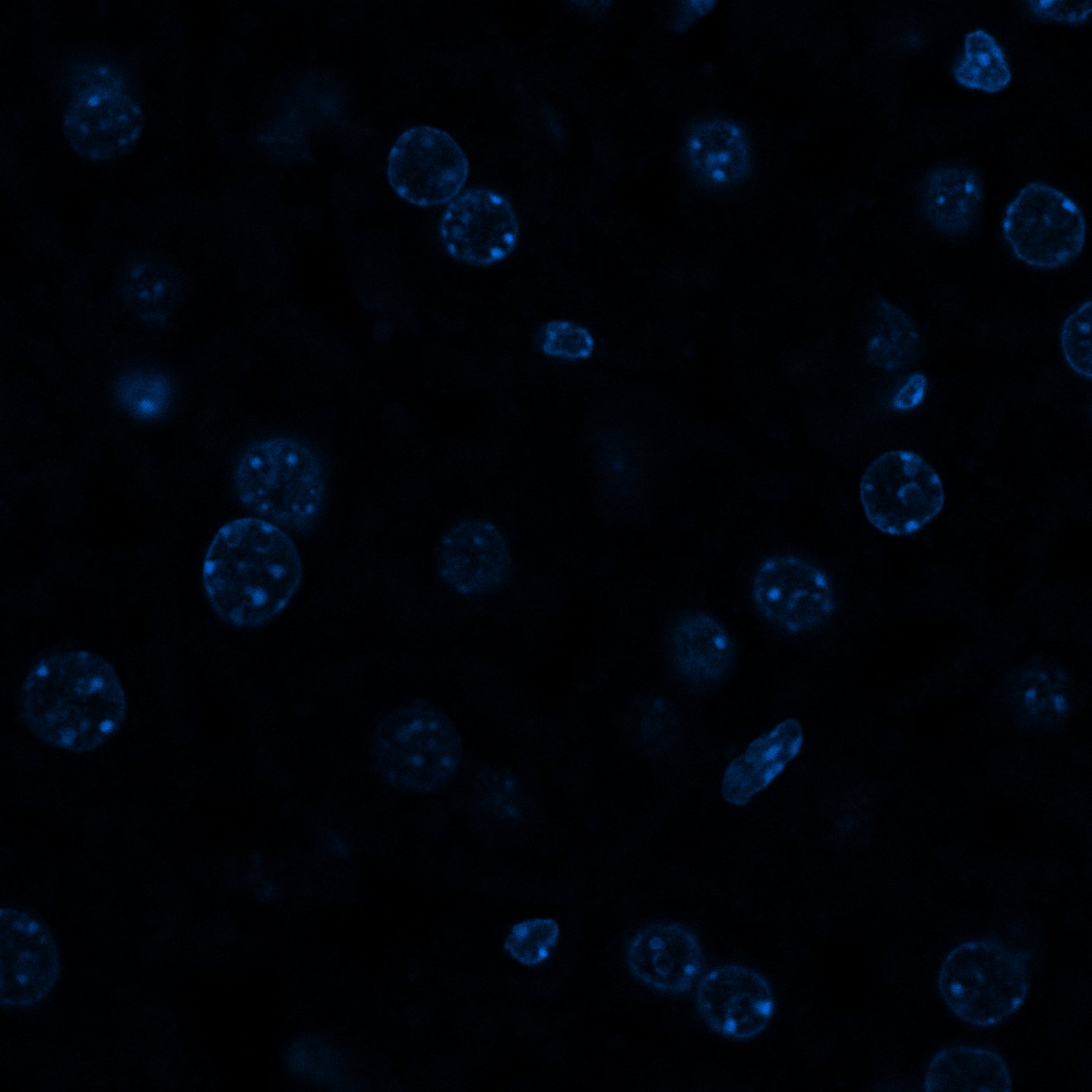

Supplement: Supplementary file 11 — Source data Fig. 4A-H [file 44319_2025_673_MOESM11_ESM.zip › 4A/FPN/Hoechst staining.jpg]

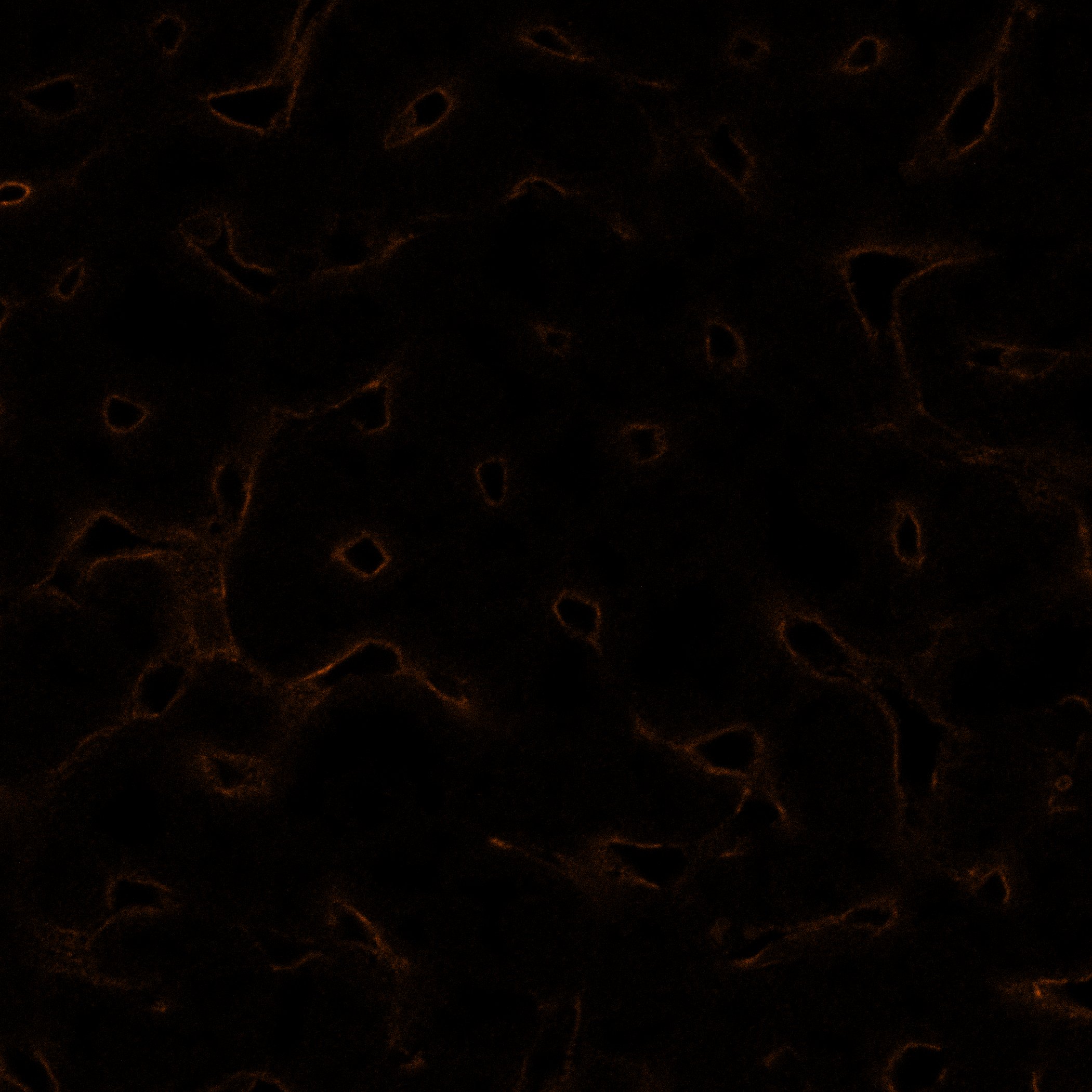

Supplement: Supplementary file 11 — Source data Fig. 4A-H [file 44319_2025_673_MOESM11_ESM.zip › 4A/HO1/CD146 staining.jpg]

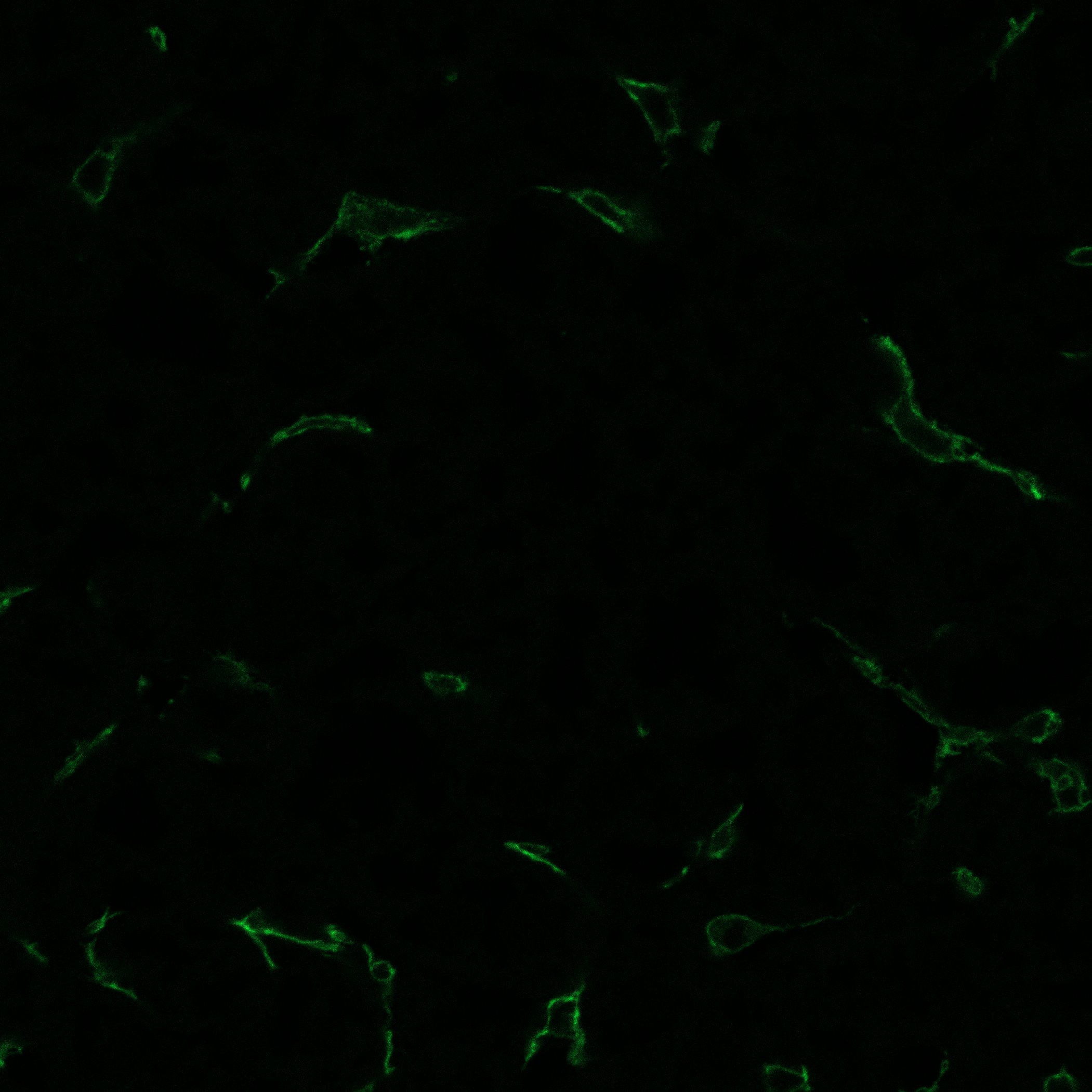

Supplement: Supplementary file 11 — Source data Fig. 4A-H [file 44319_2025_673_MOESM11_ESM.zip › 4A/HO1/F480 staining.jpg]

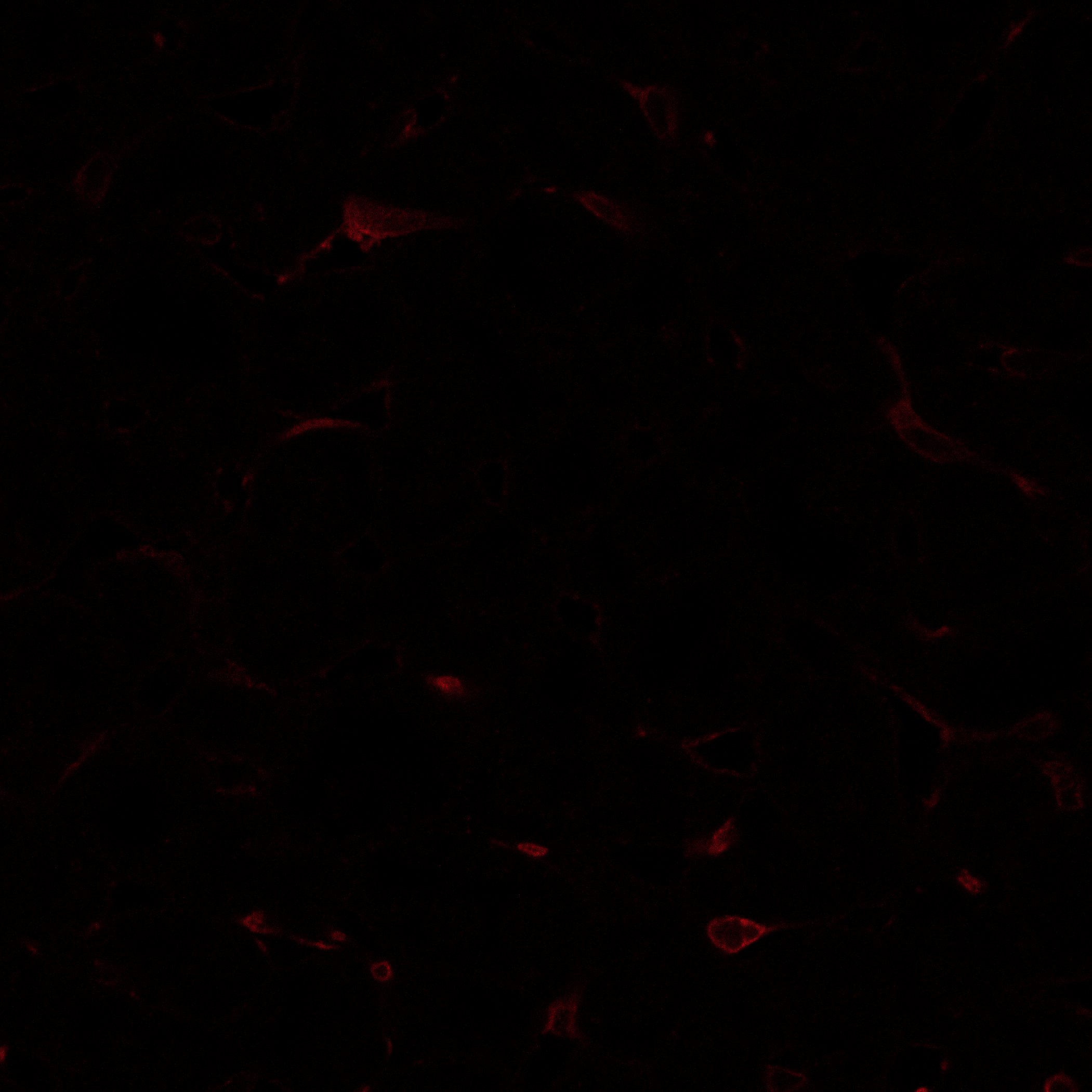

Supplement: Supplementary file 11 — Source data Fig. 4A-H [file 44319_2025_673_MOESM11_ESM.zip › 4A/HO1/HO1 staining.jpg]

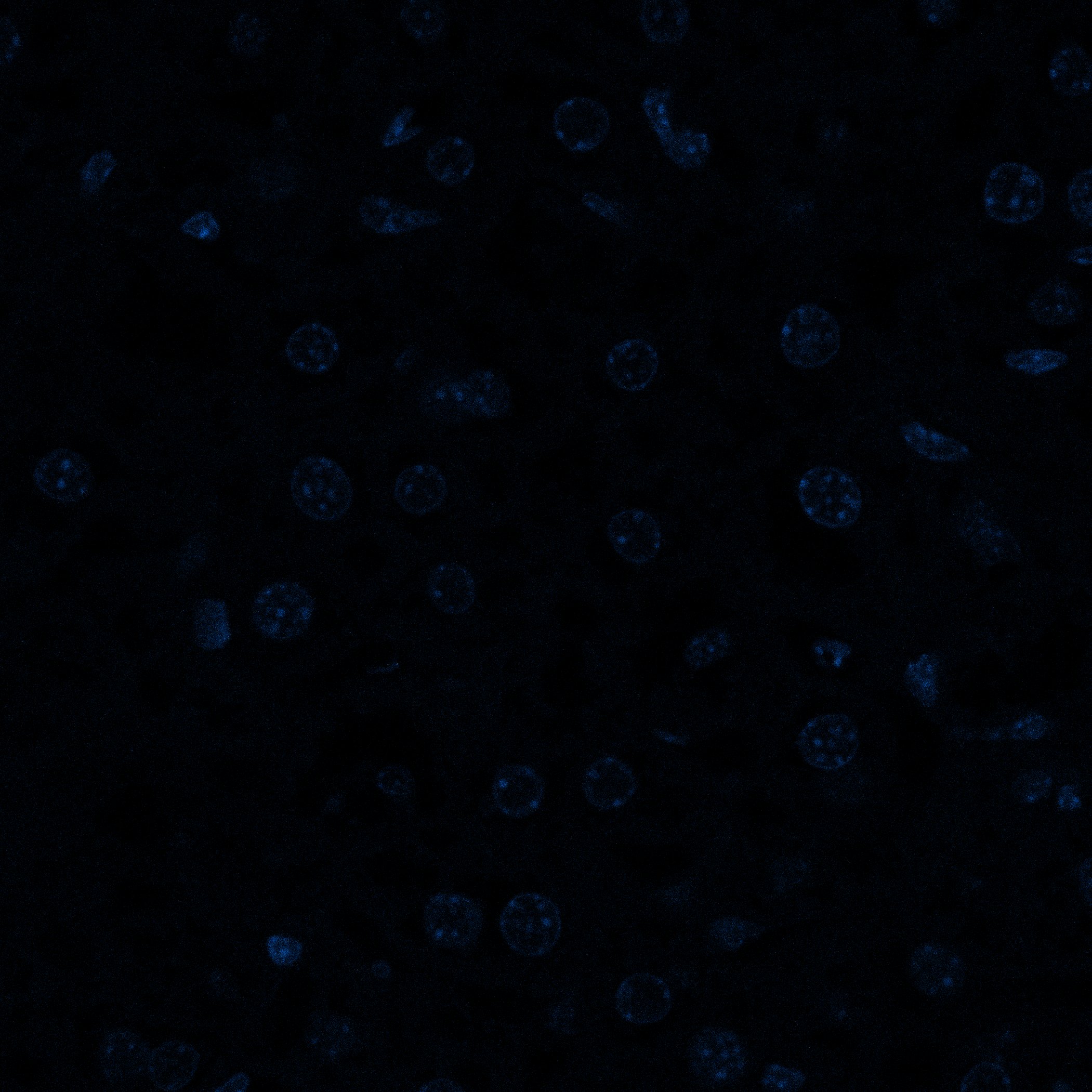

Supplement: Supplementary file 11 — Source data Fig. 4A-H [file 44319_2025_673_MOESM11_ESM.zip › 4A/HO1/Hoechst staining.jpg]

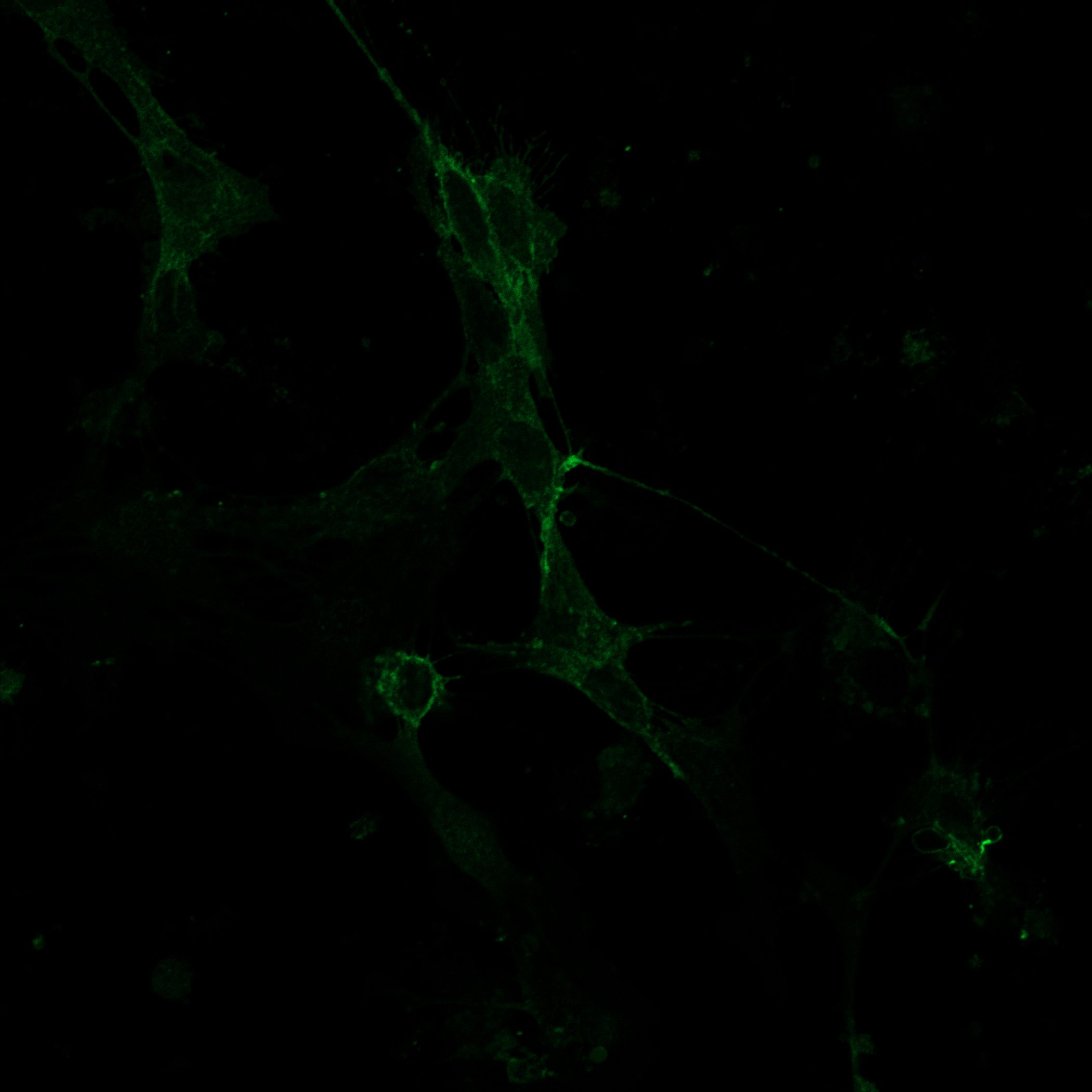

Supplement: Supplementary file 11 — Source data Fig. 4A-H [file 44319_2025_673_MOESM11_ESM.zip › 4B/FPN/CD31 staining.jpg]

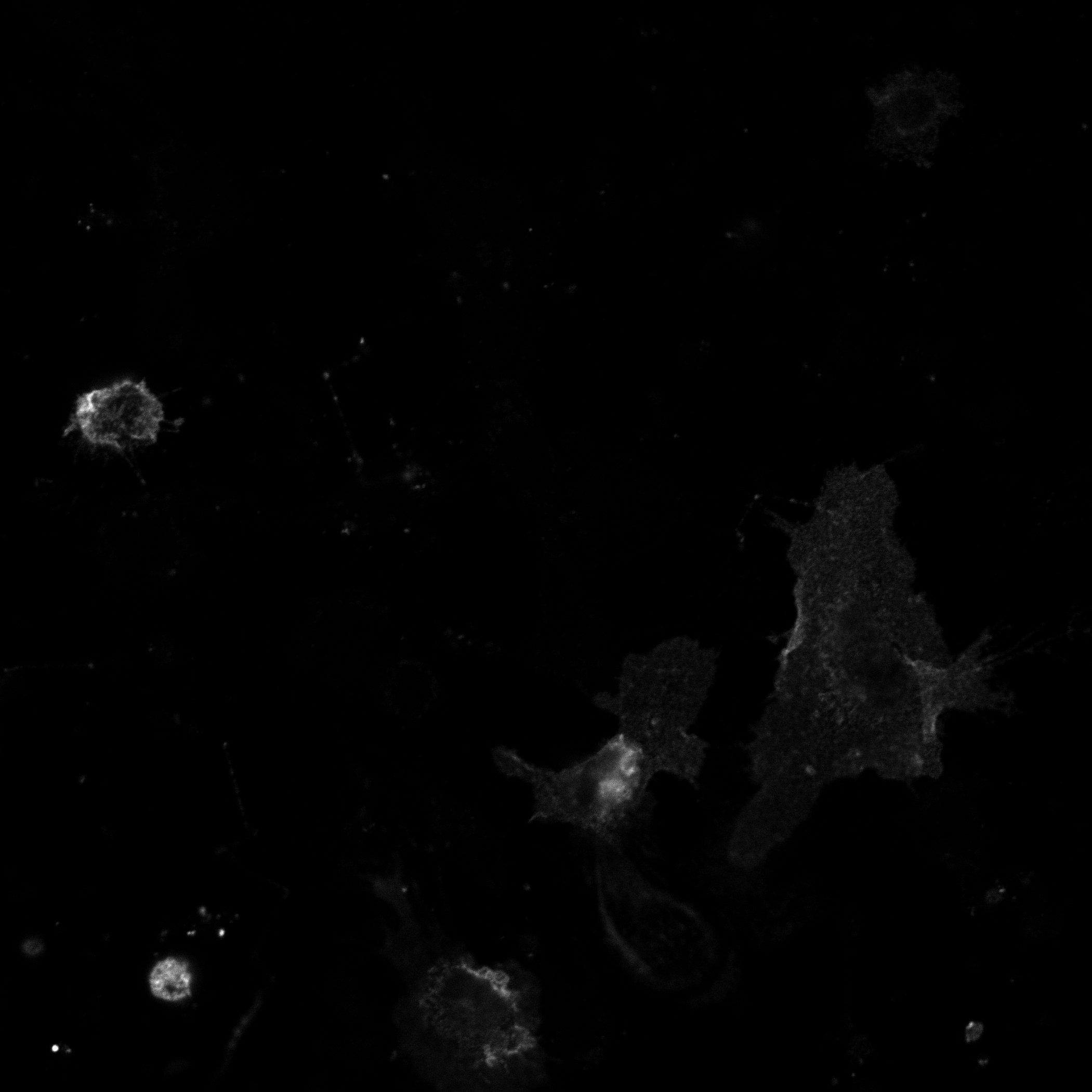

Supplement: Supplementary file 11 — Source data Fig. 4A-H [file 44319_2025_673_MOESM11_ESM.zip › 4B/FPN/F480 staining.jpg]

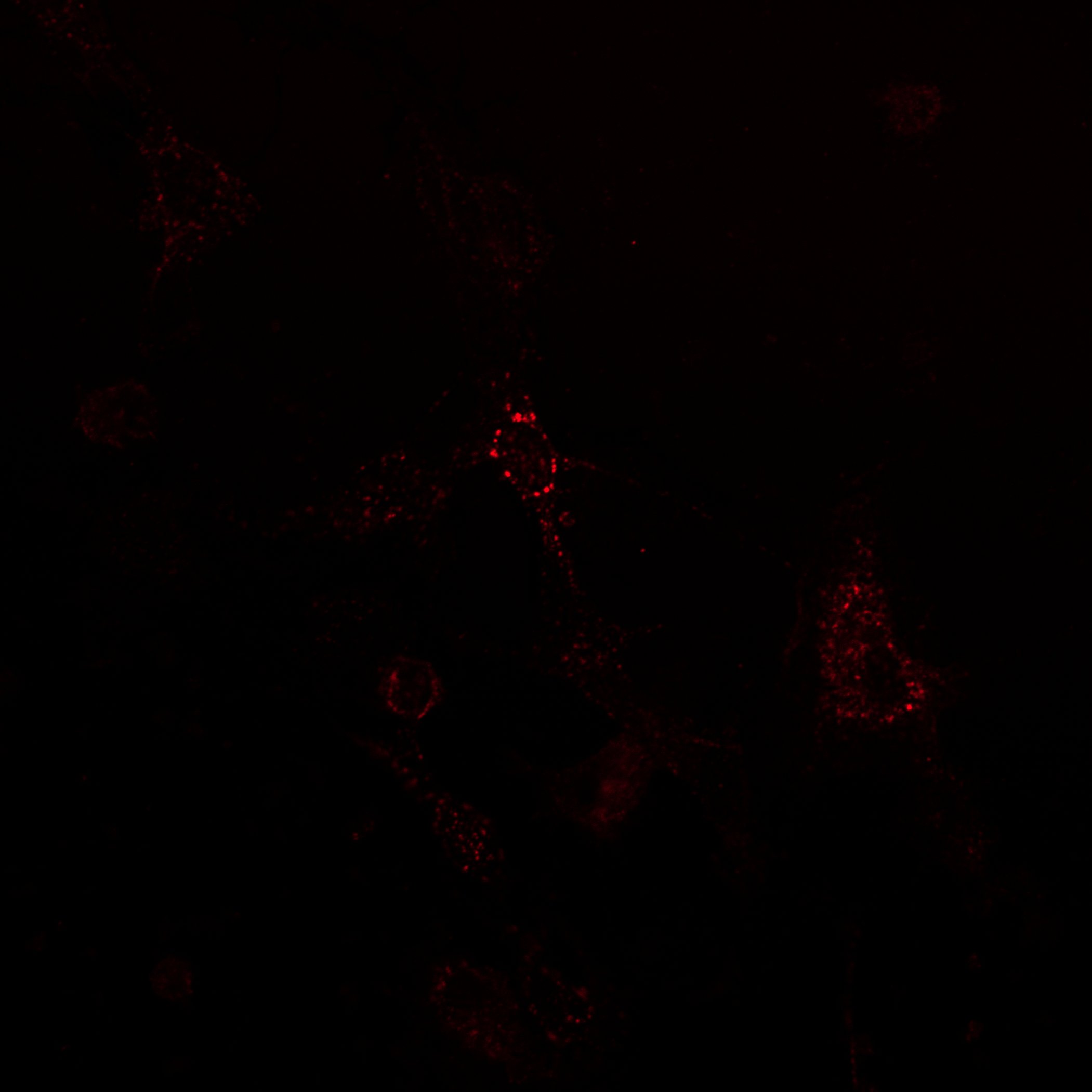

Supplement: Supplementary file 11 — Source data Fig. 4A-H [file 44319_2025_673_MOESM11_ESM.zip › 4B/FPN/FPN staining.jpg]

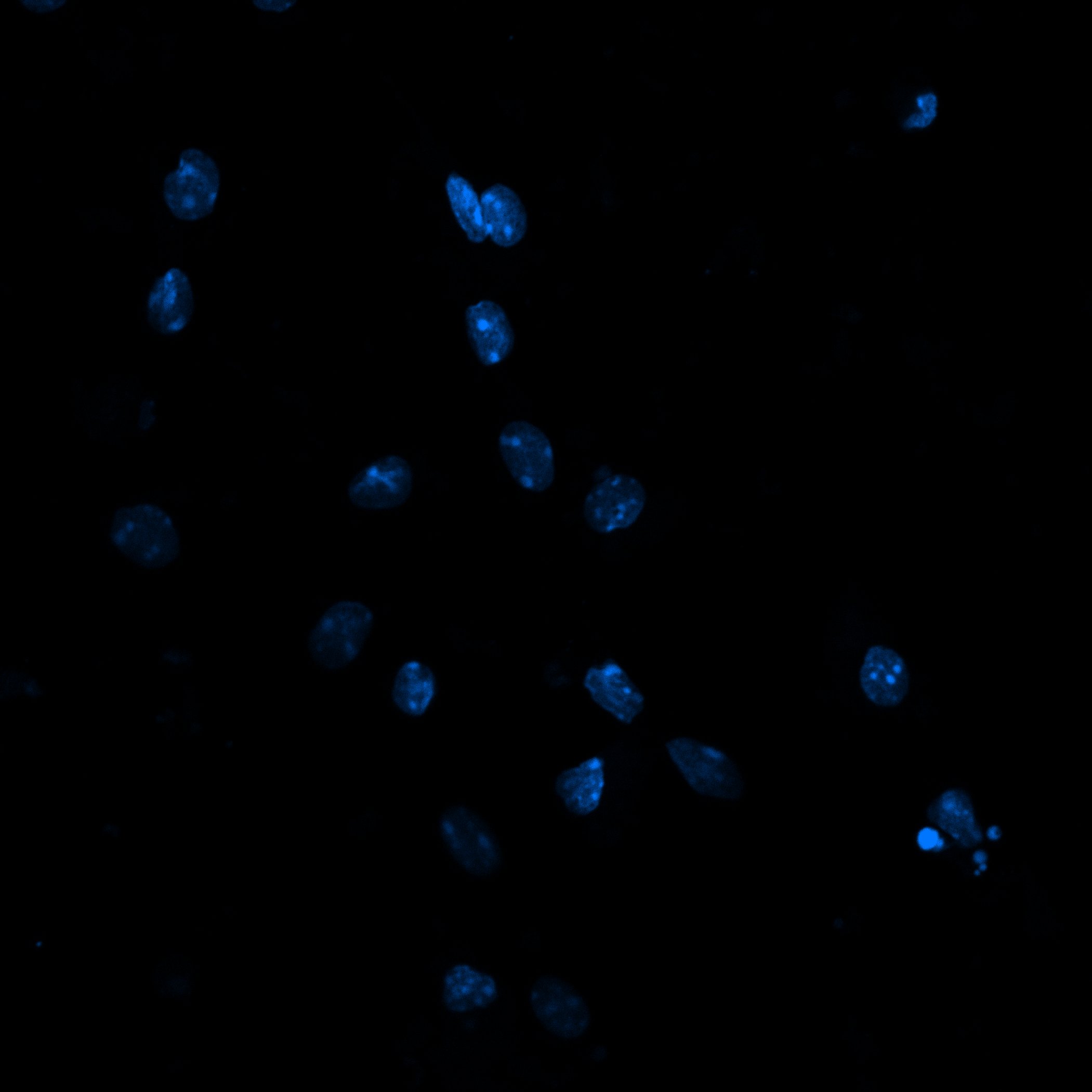

Supplement: Supplementary file 11 — Source data Fig. 4A-H [file 44319_2025_673_MOESM11_ESM.zip › 4B/FPN/Hoechst staining.jpg]

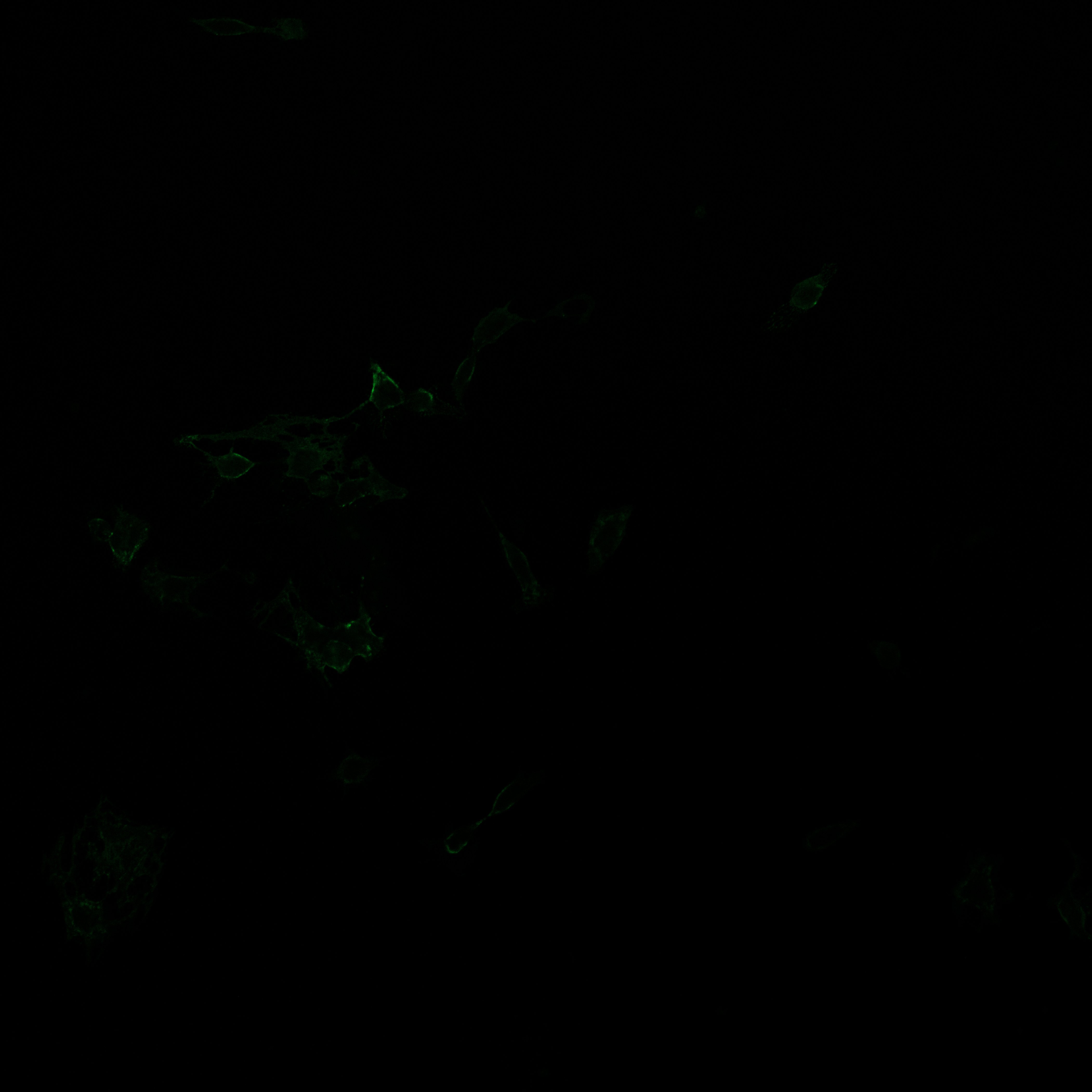

Supplement: Supplementary file 11 — Source data Fig. 4A-H [file 44319_2025_673_MOESM11_ESM.zip › 4B/HO1/CD31 staining.jpg]

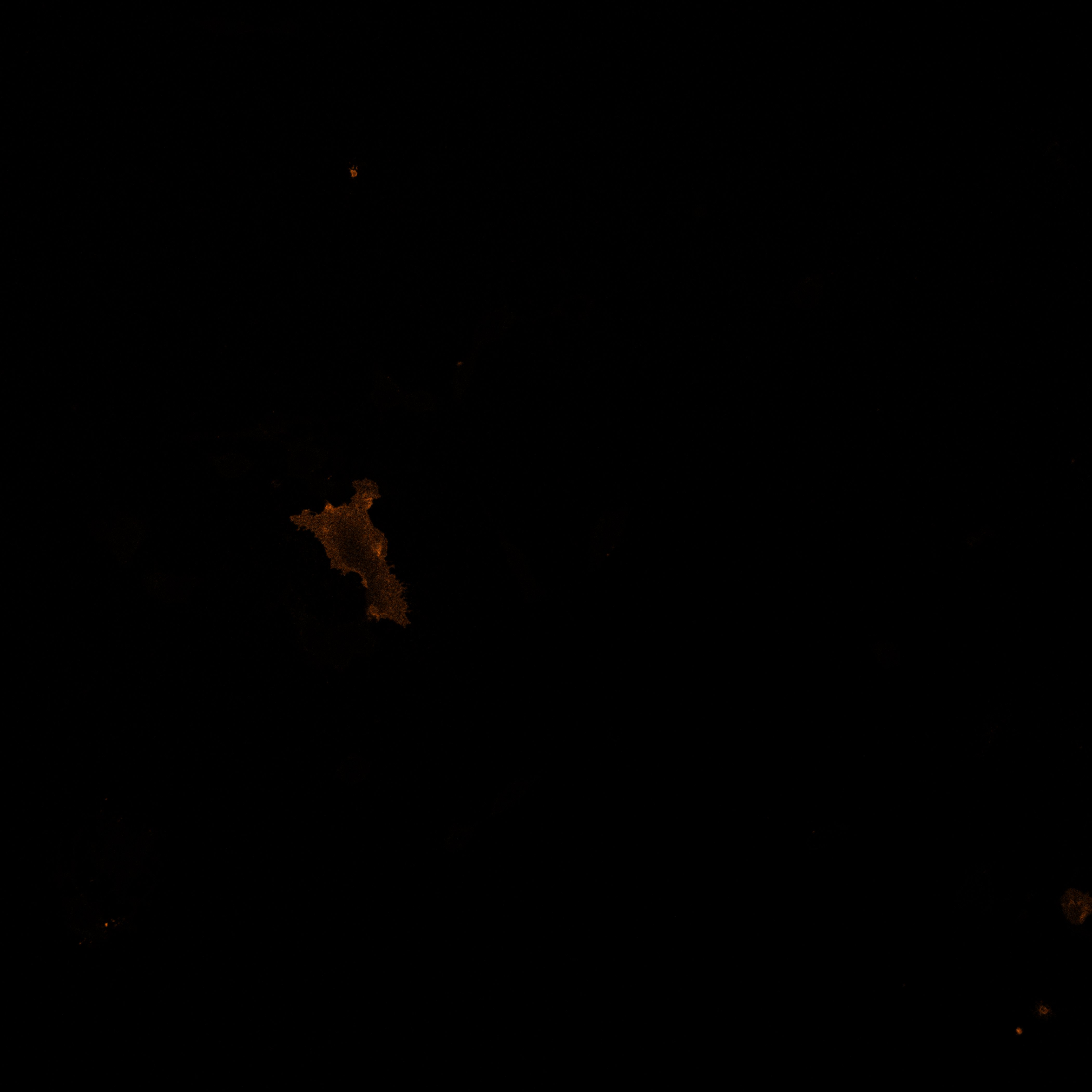

Supplement: Supplementary file 11 — Source data Fig. 4A-H [file 44319_2025_673_MOESM11_ESM.zip › 4B/HO1/F480 staining.jpg]

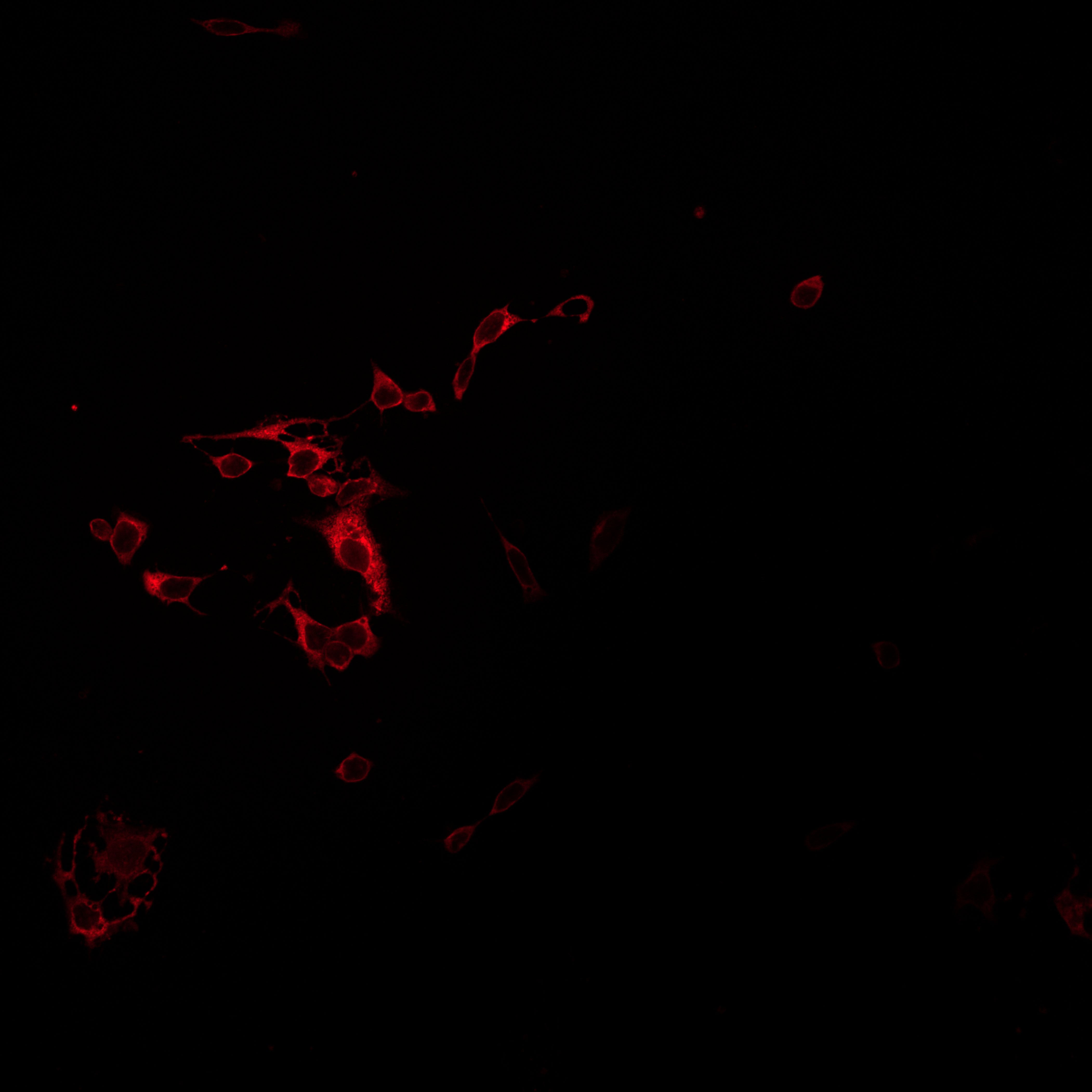

Supplement: Supplementary file 11 — Source data Fig. 4A-H [file 44319_2025_673_MOESM11_ESM.zip › 4B/HO1/HO1 staining.jpg]

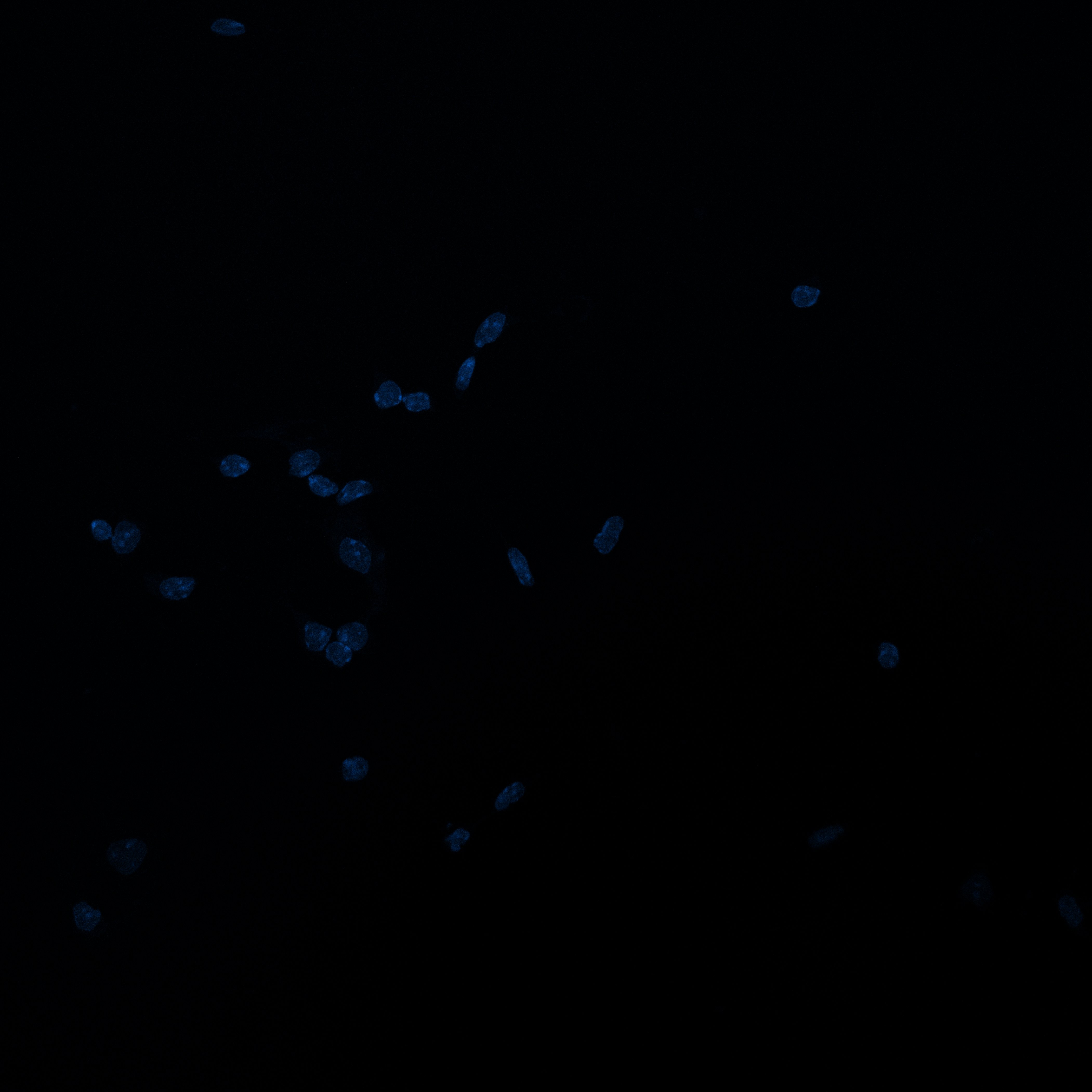

Supplement: Supplementary file 11 — Source data Fig. 4A-H [file 44319_2025_673_MOESM11_ESM.zip › 4B/HO1/Hoechst staining.jpg]

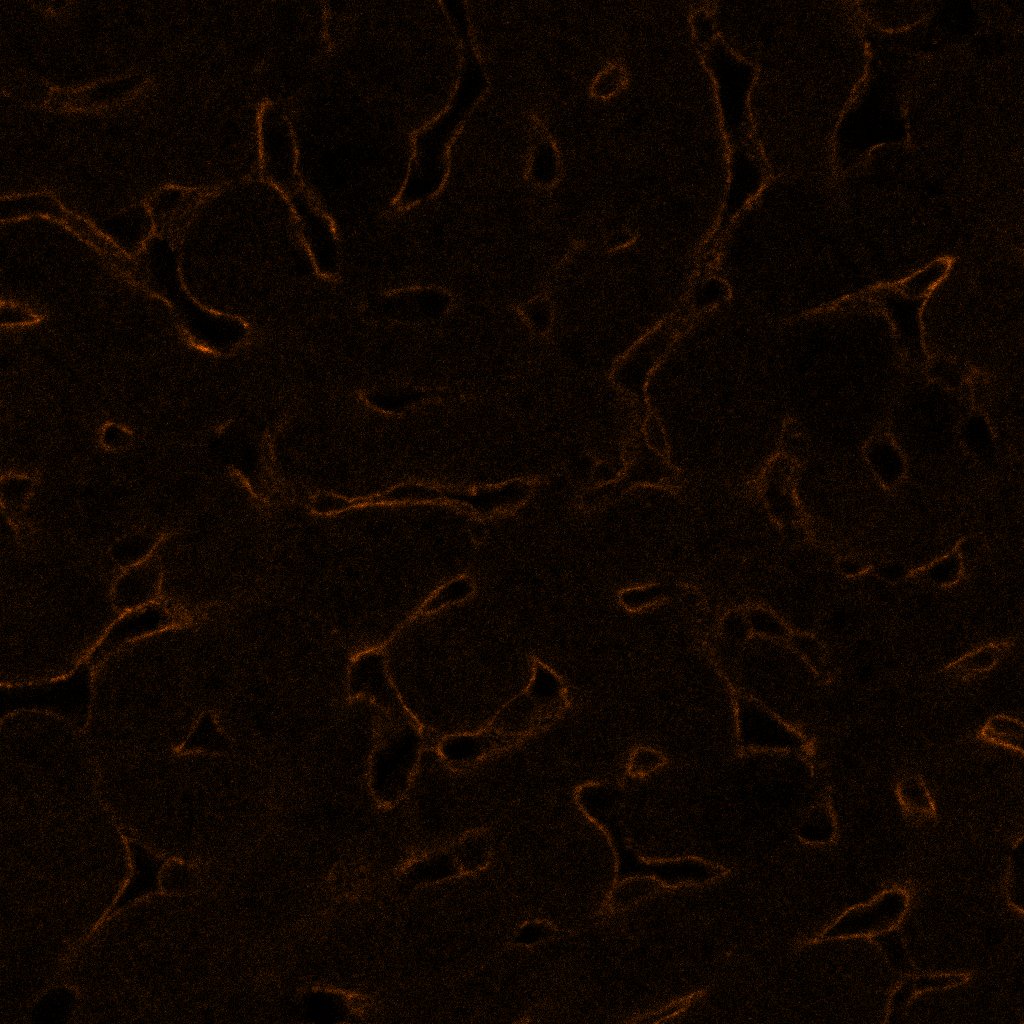

Supplement: Supplementary file 16 — Source data Fig. 6 [file 44319_2025_673_MOESM16_ESM.zip › Figure 6/Fig. 6E/CTR/Cd146 staining.jpg]

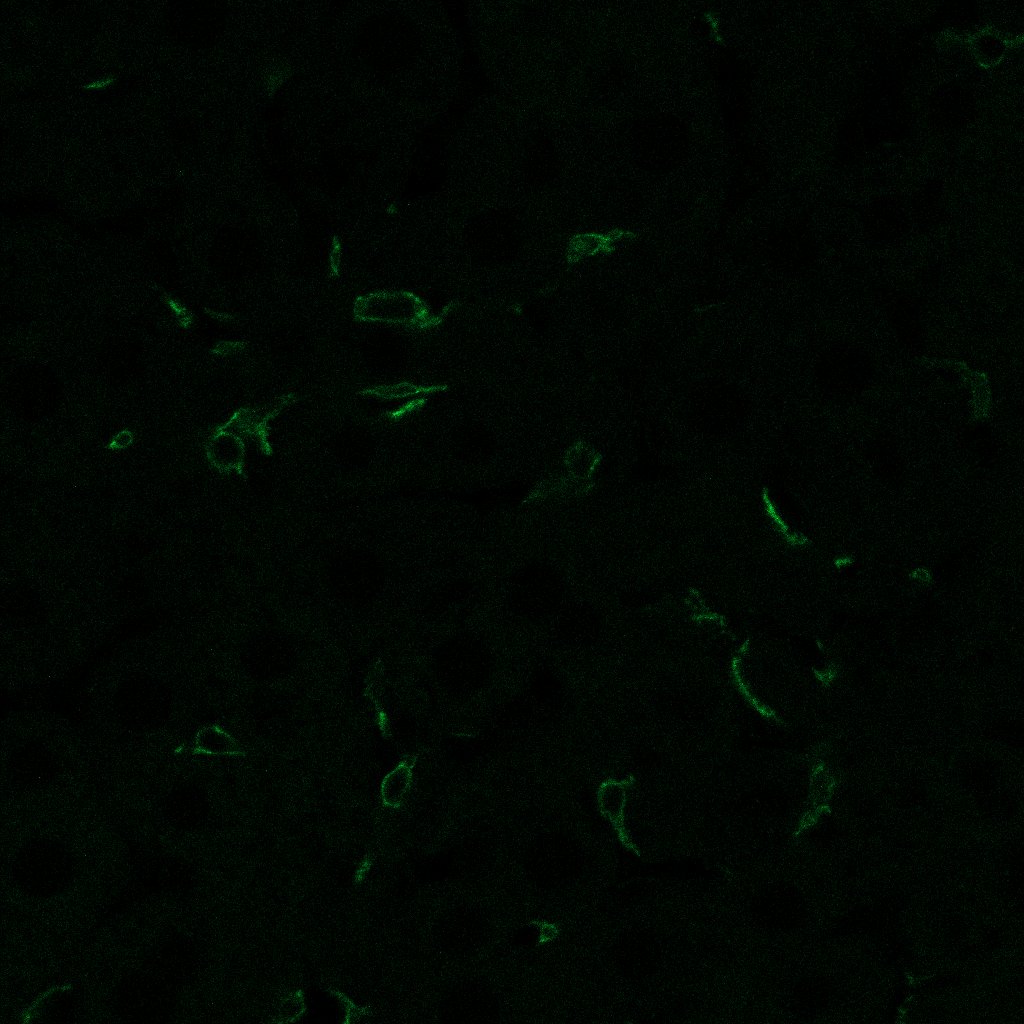

Supplement: Supplementary file 16 — Source data Fig. 6 [file 44319_2025_673_MOESM16_ESM.zip › Figure 6/Fig. 6E/CTR/F480 staining.jpg]

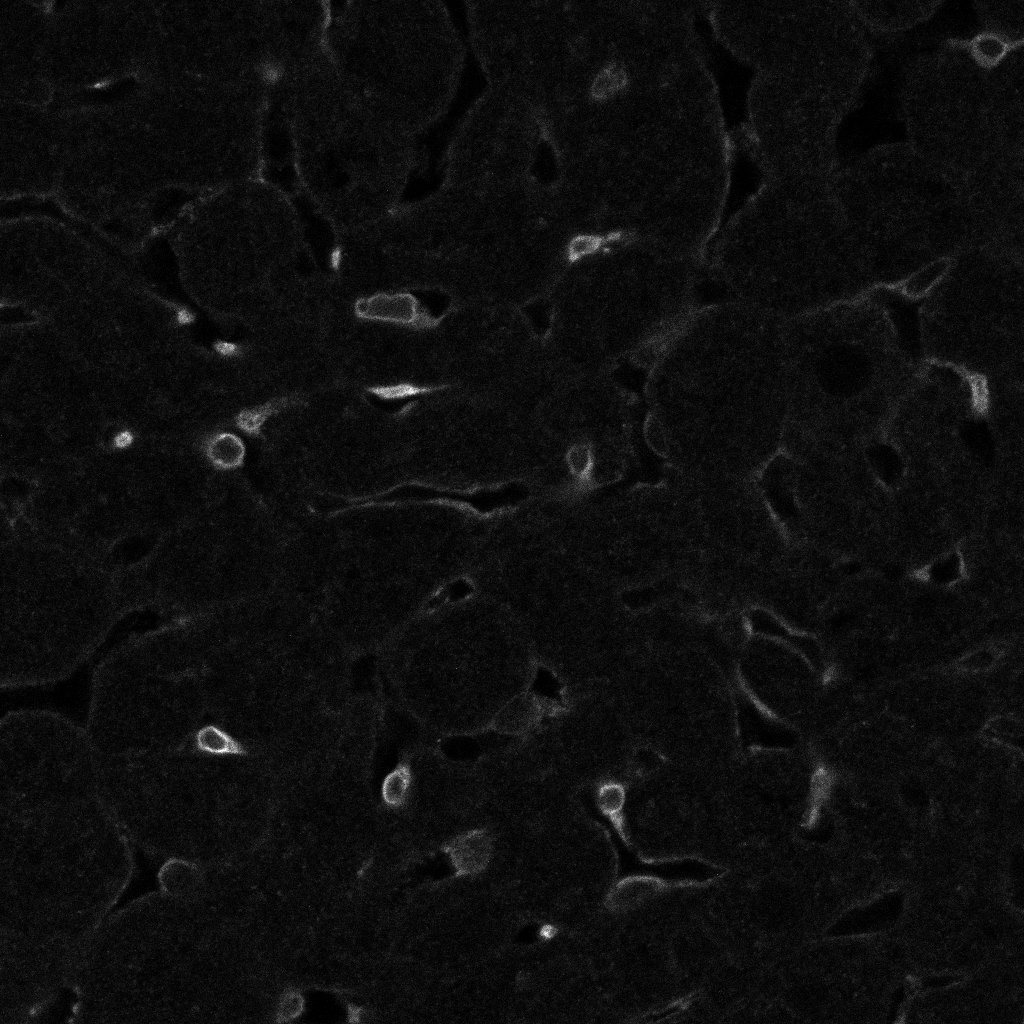

Supplement: Supplementary file 16 — Source data Fig. 6 [file 44319_2025_673_MOESM16_ESM.zip › Figure 6/Fig. 6E/CTR/HO1 staining.jpg]

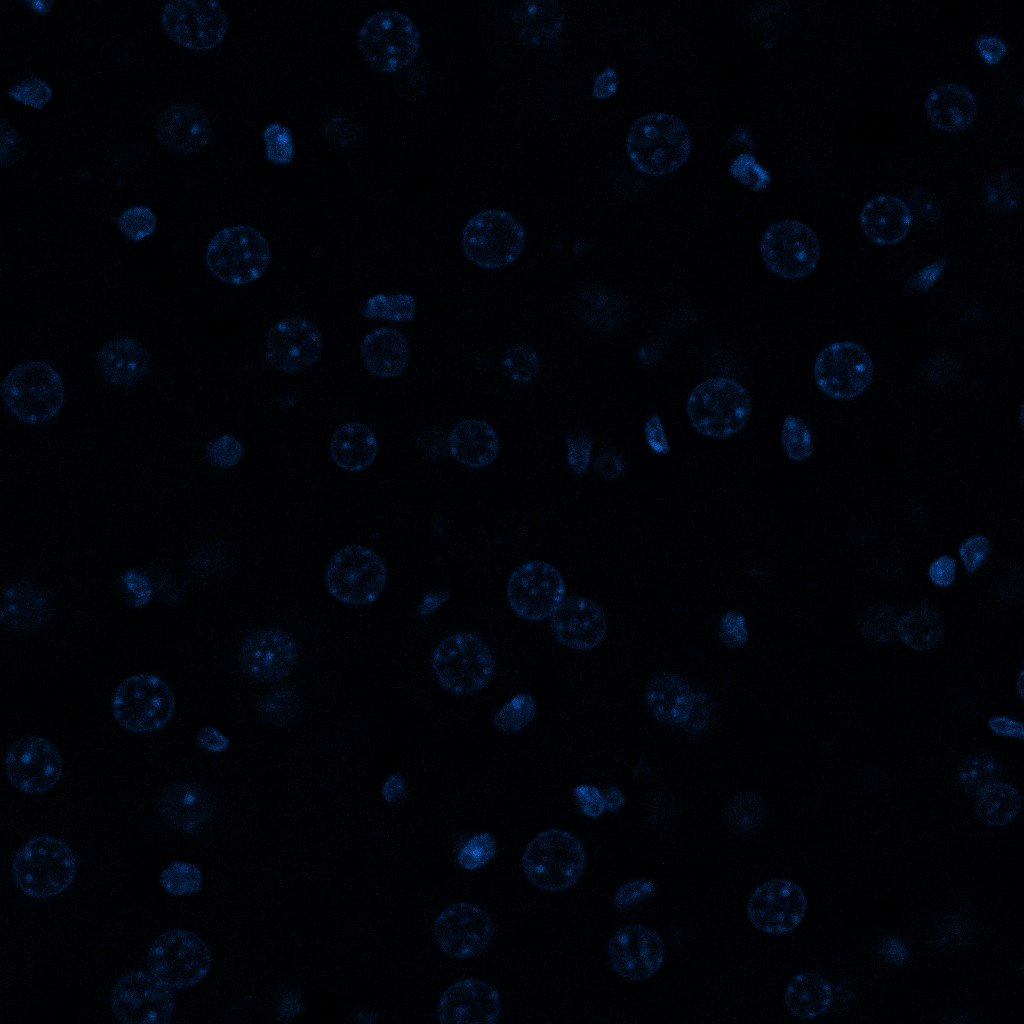

Supplement: Supplementary file 16 — Source data Fig. 6 [file 44319_2025_673_MOESM16_ESM.zip › Figure 6/Fig. 6E/CTR/Hoechst staining.jpg]

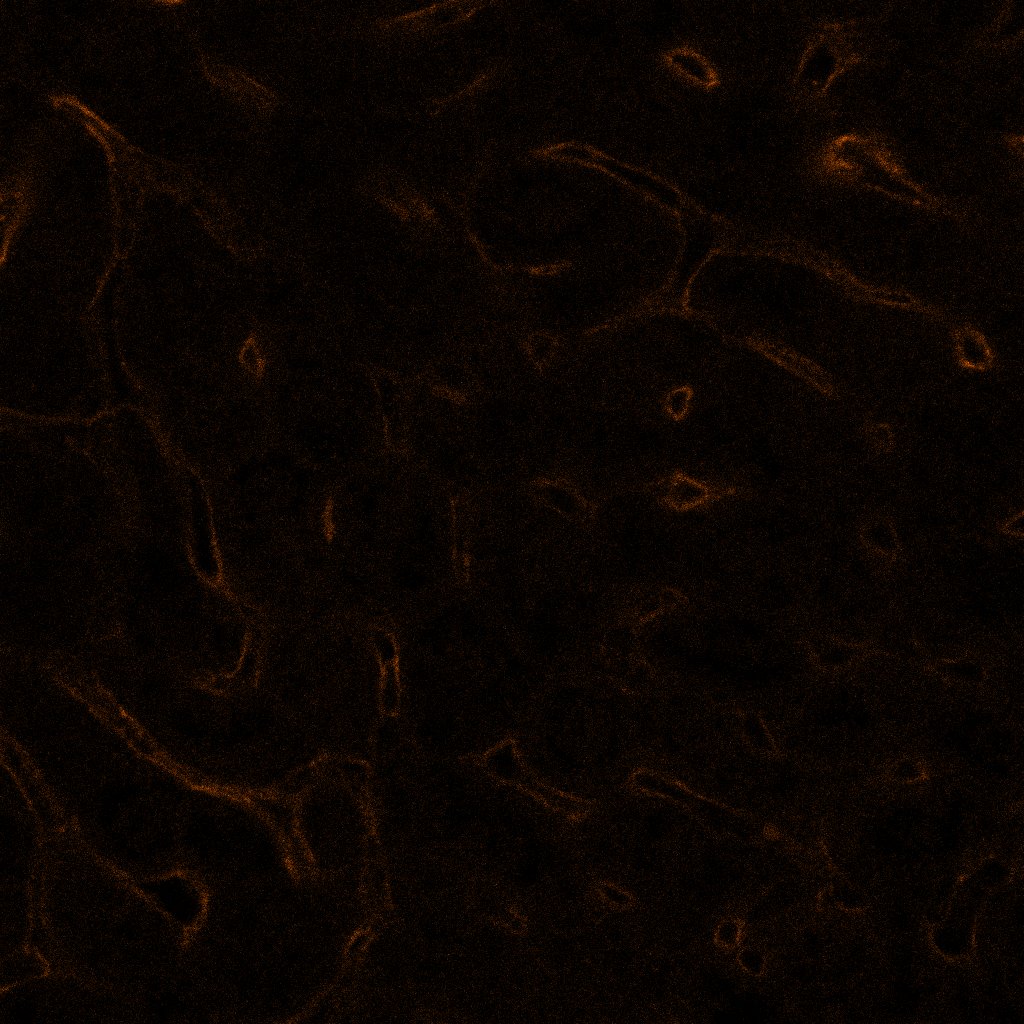

Supplement: Supplementary file 16 — Source data Fig. 6 [file 44319_2025_673_MOESM16_ESM.zip › Figure 6/Fig. 6E/HGB 6h/CD146 staining.jpg]

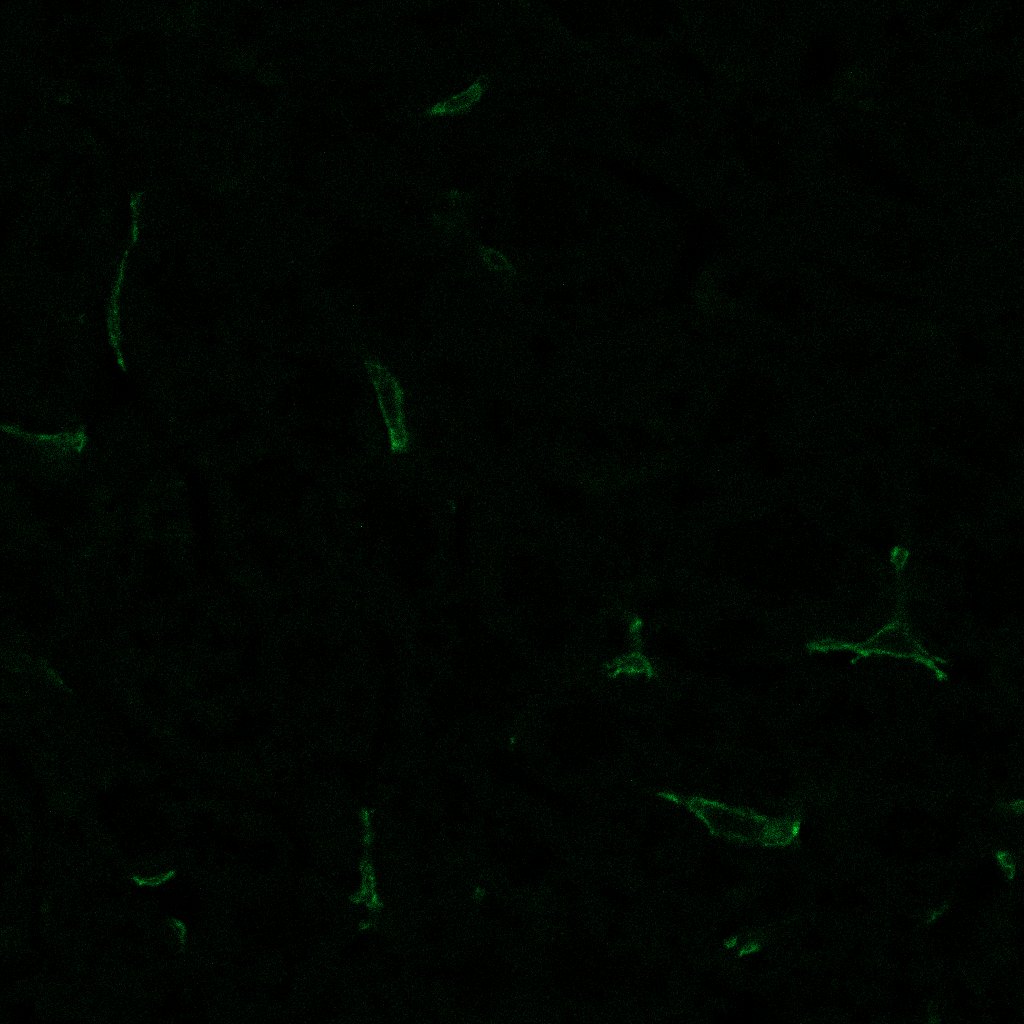

Supplement: Supplementary file 16 — Source data Fig. 6 [file 44319_2025_673_MOESM16_ESM.zip › Figure 6/Fig. 6E/HGB 6h/F480 staining.jpg]

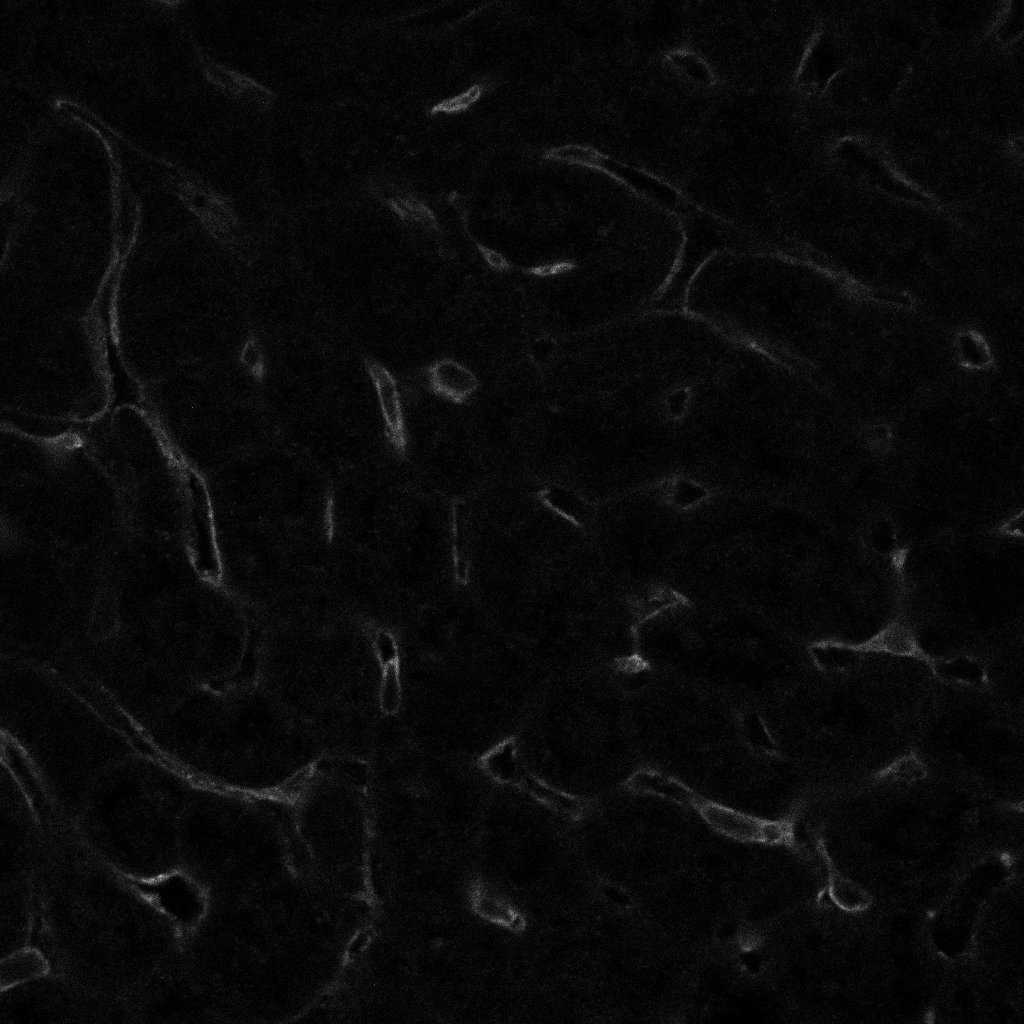

Supplement: Supplementary file 16 — Source data Fig. 6 [file 44319_2025_673_MOESM16_ESM.zip › Figure 6/Fig. 6E/HGB 6h/HO1 staining.jpg]

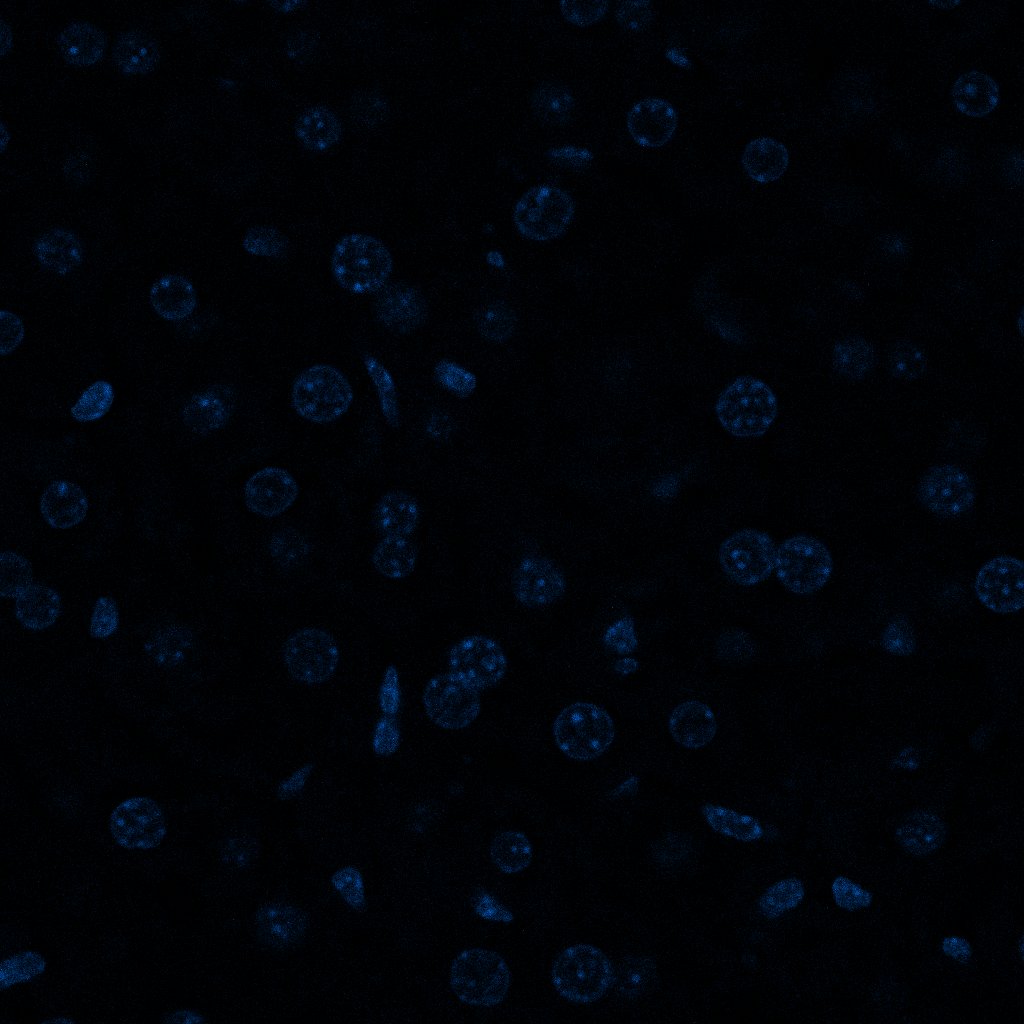

Supplement: Supplementary file 16 — Source data Fig. 6 [file 44319_2025_673_MOESM16_ESM.zip › Figure 6/Fig. 6E/HGB 6h/Hoechst staining.jpg]

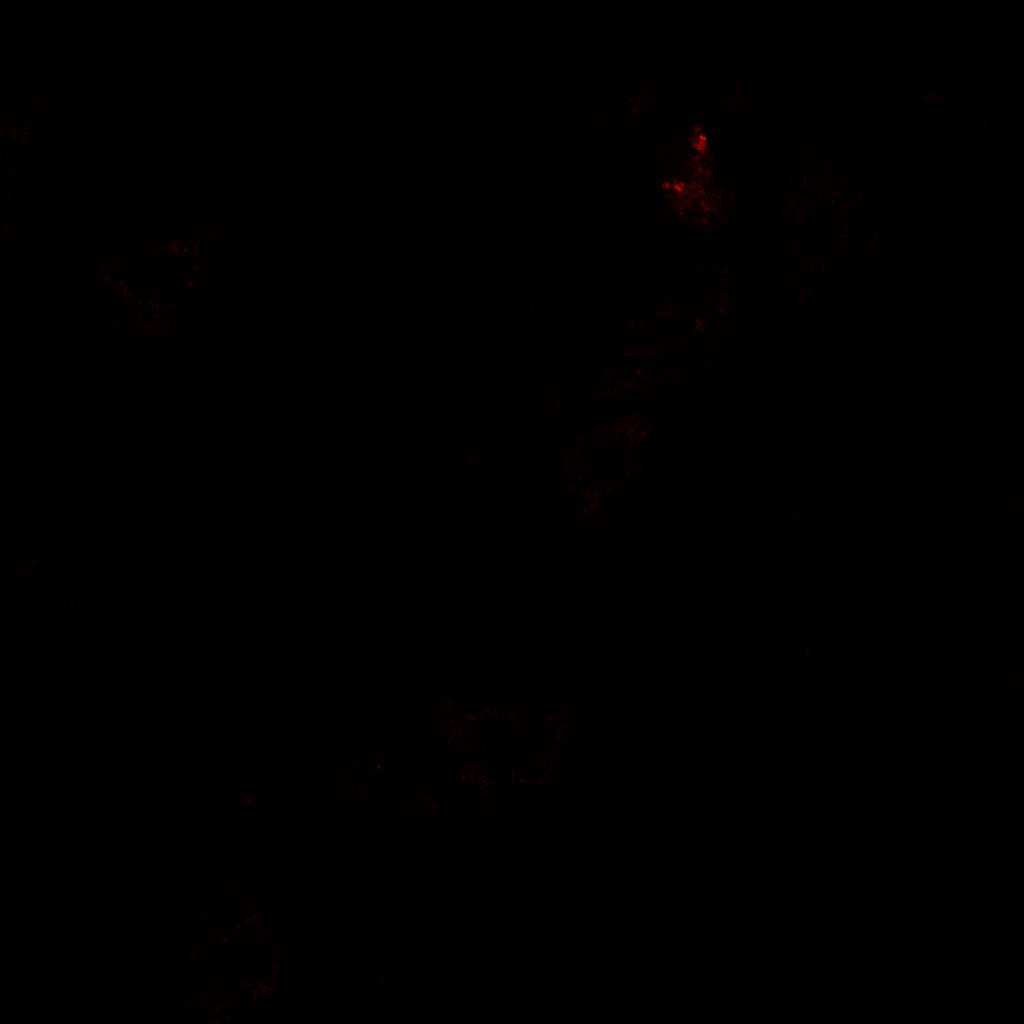

Supplement: Supplementary file 18 — Figure EV2C Source Data [file 44319_2025_673_MOESM18_ESM.zip › EV2C/Control/Control_Hb.jpg]

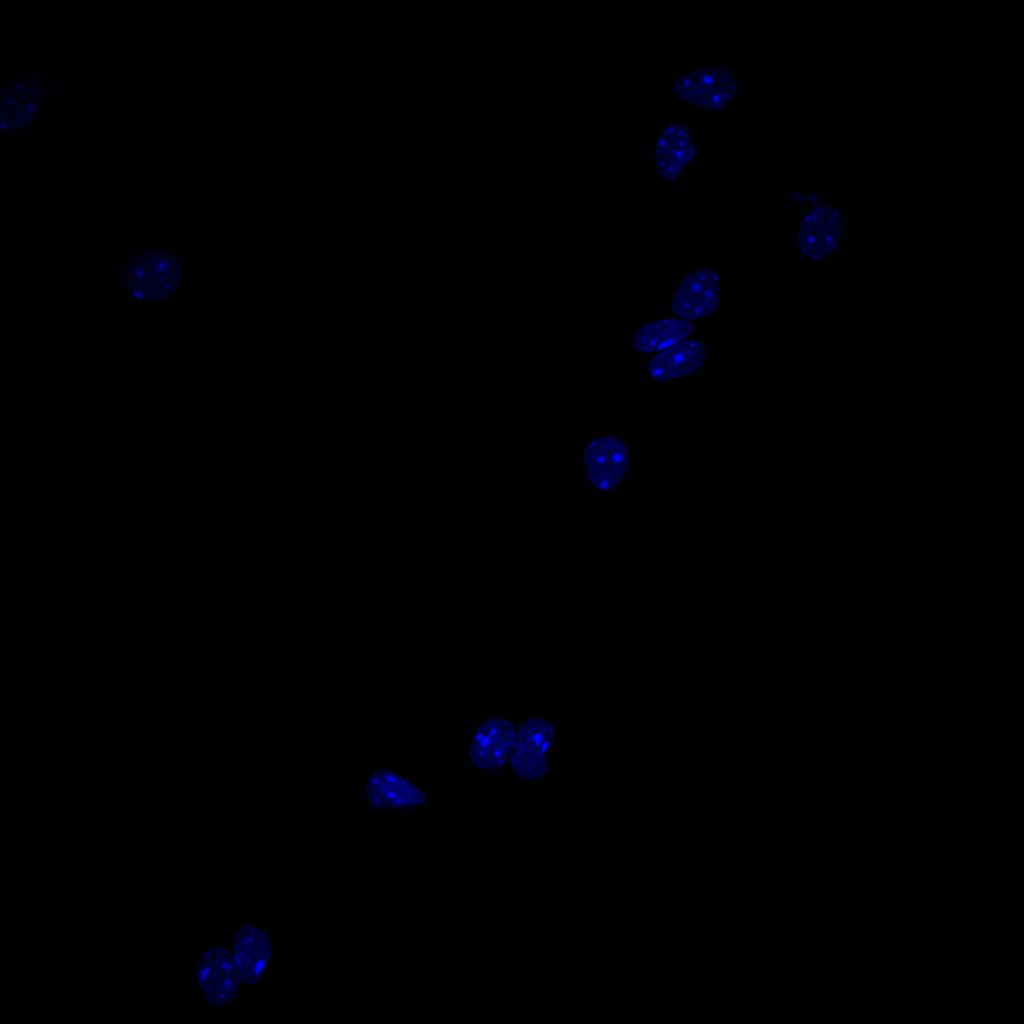

Supplement: Supplementary file 18 — Figure EV2C Source Data [file 44319_2025_673_MOESM18_ESM.zip › EV2C/Control/Control_hoechst staining.jpg]

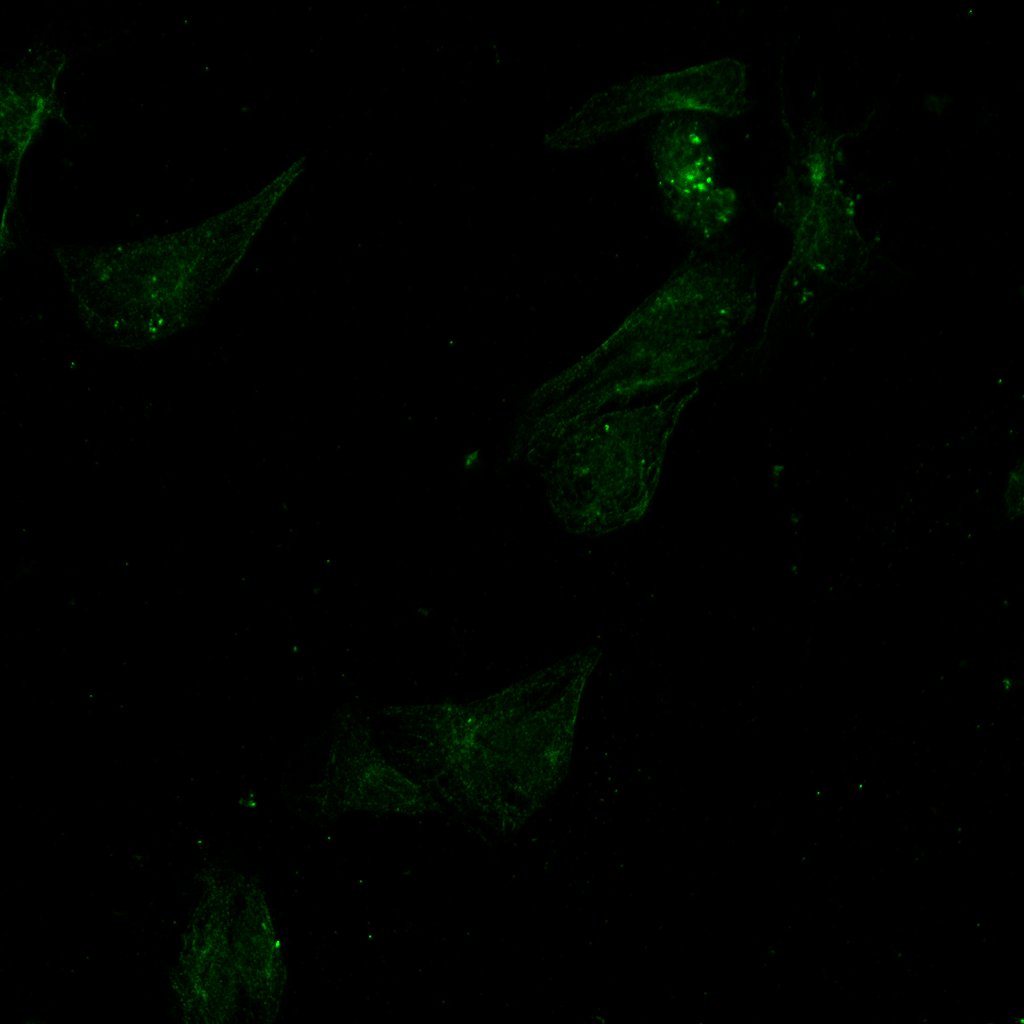

Supplement: Supplementary file 18 — Figure EV2C Source Data [file 44319_2025_673_MOESM18_ESM.zip › EV2C/Control/Control_Stab2 staining.jpg]

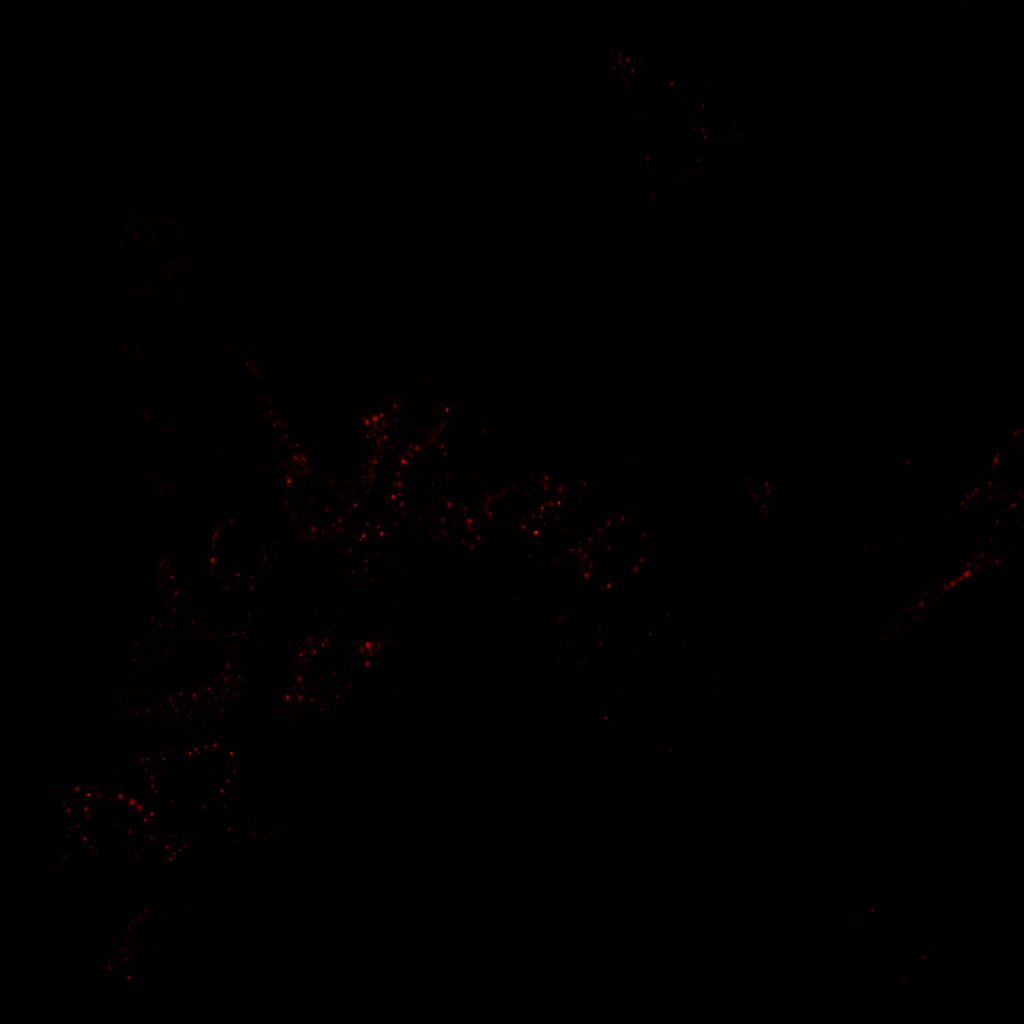

Supplement: Supplementary file 18 — Figure EV2C Source Data [file 44319_2025_673_MOESM18_ESM.zip › EV2C/CPZ+Hb/CPZ+Hb_Hb.jpg]

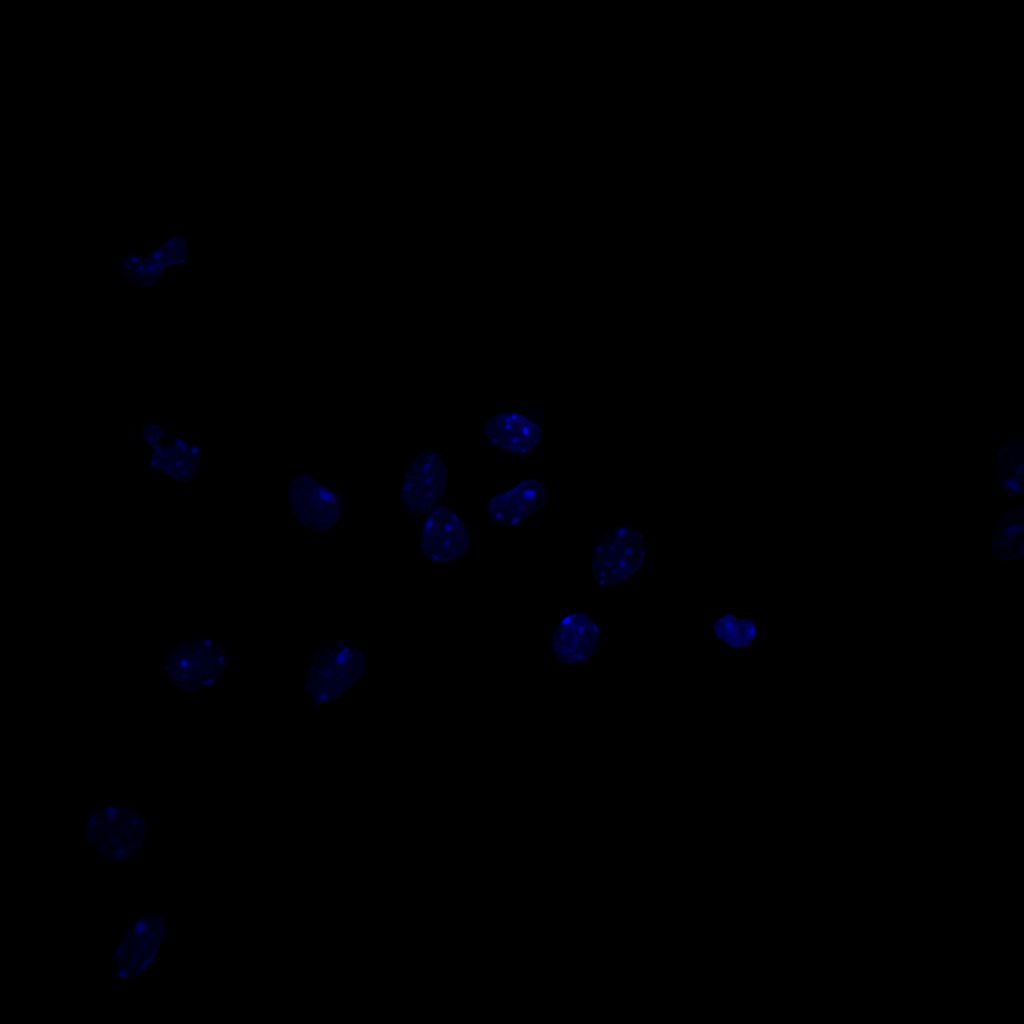

Supplement: Supplementary file 18 — Figure EV2C Source Data [file 44319_2025_673_MOESM18_ESM.zip › EV2C/CPZ+Hb/CPZ+Hb_hoechst staining.jpg]

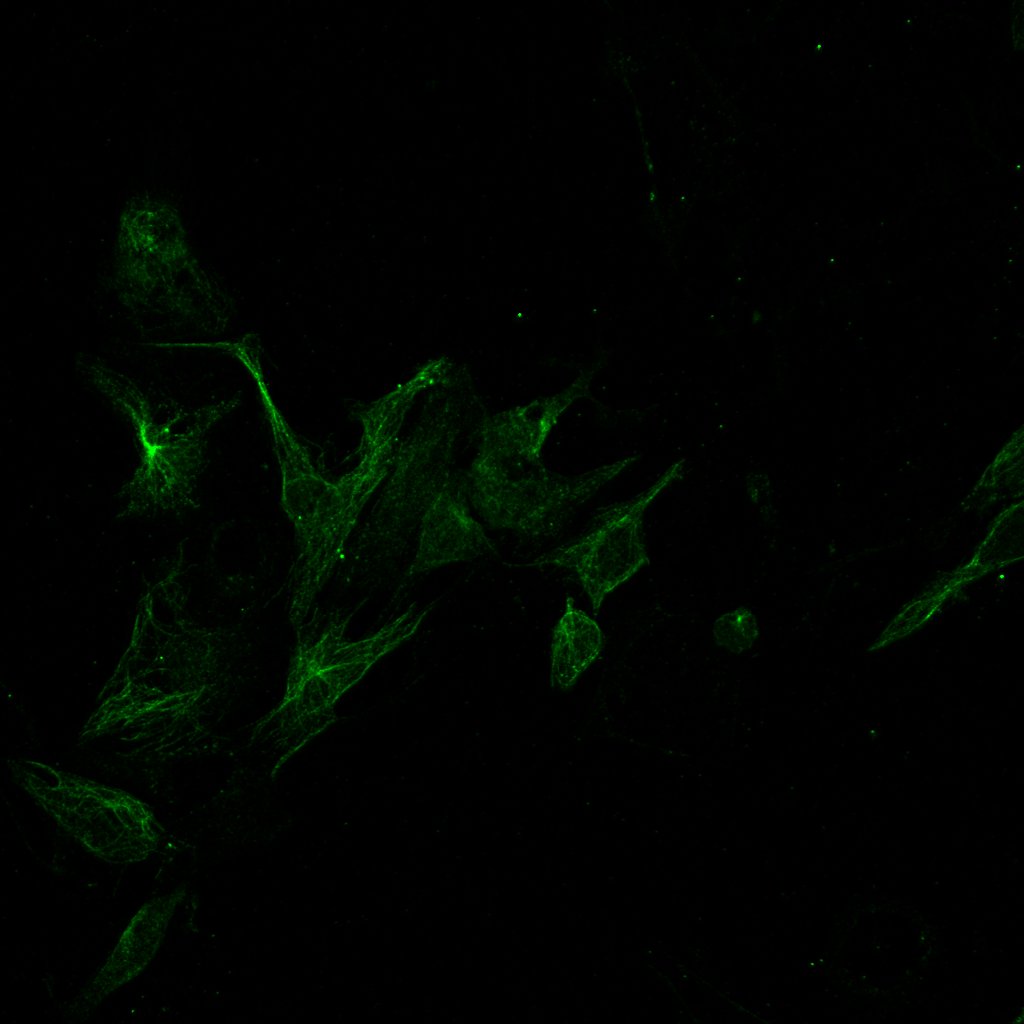

Supplement: Supplementary file 18 — Figure EV2C Source Data [file 44319_2025_673_MOESM18_ESM.zip › EV2C/CPZ+Hb/CPZ+Hb_Stab2 staining.jpg]

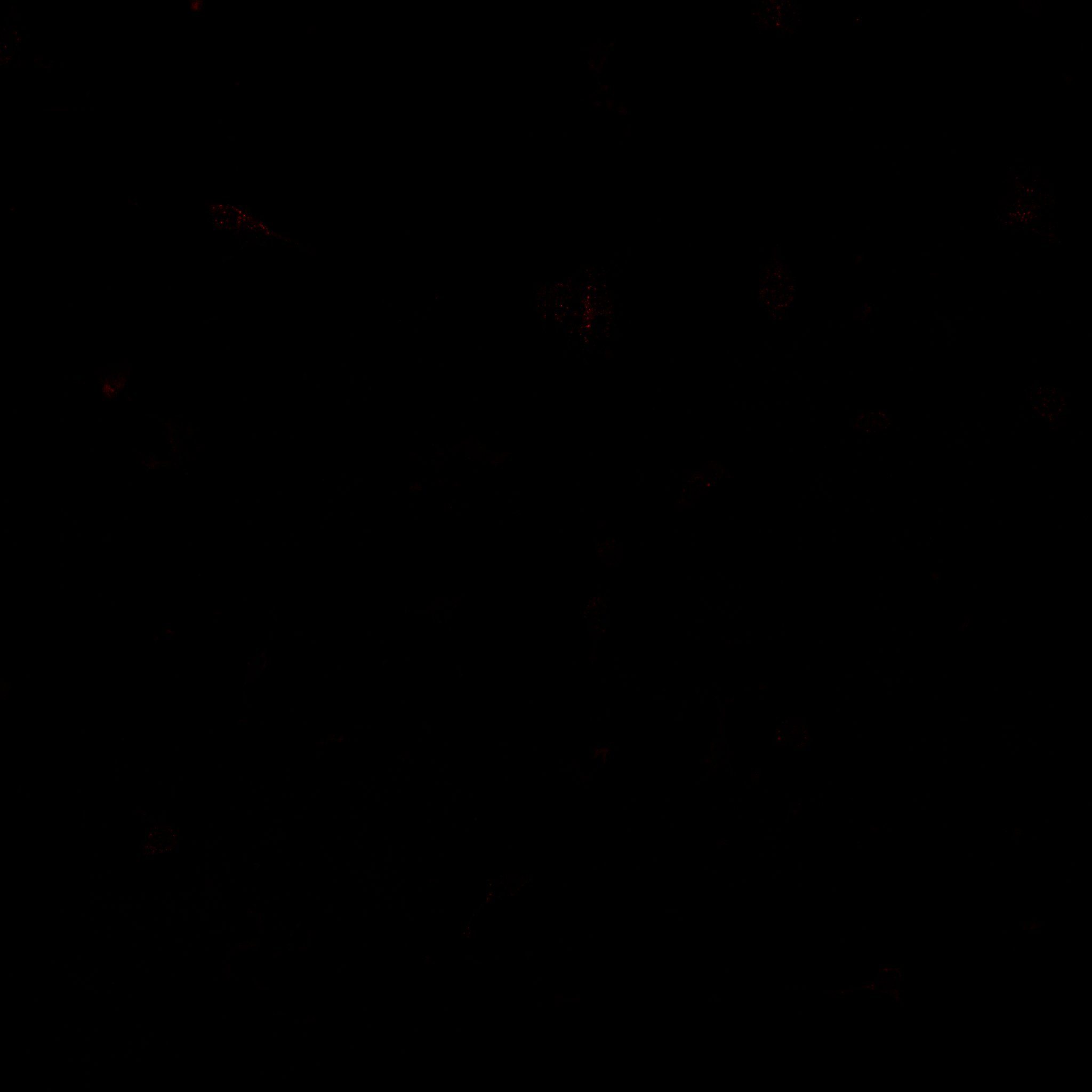

Supplement: Supplementary file 18 — Figure EV2C Source Data [file 44319_2025_673_MOESM18_ESM.zip › EV2C/EIPA+Hb/EIPA+Hb_Hb.jpg]

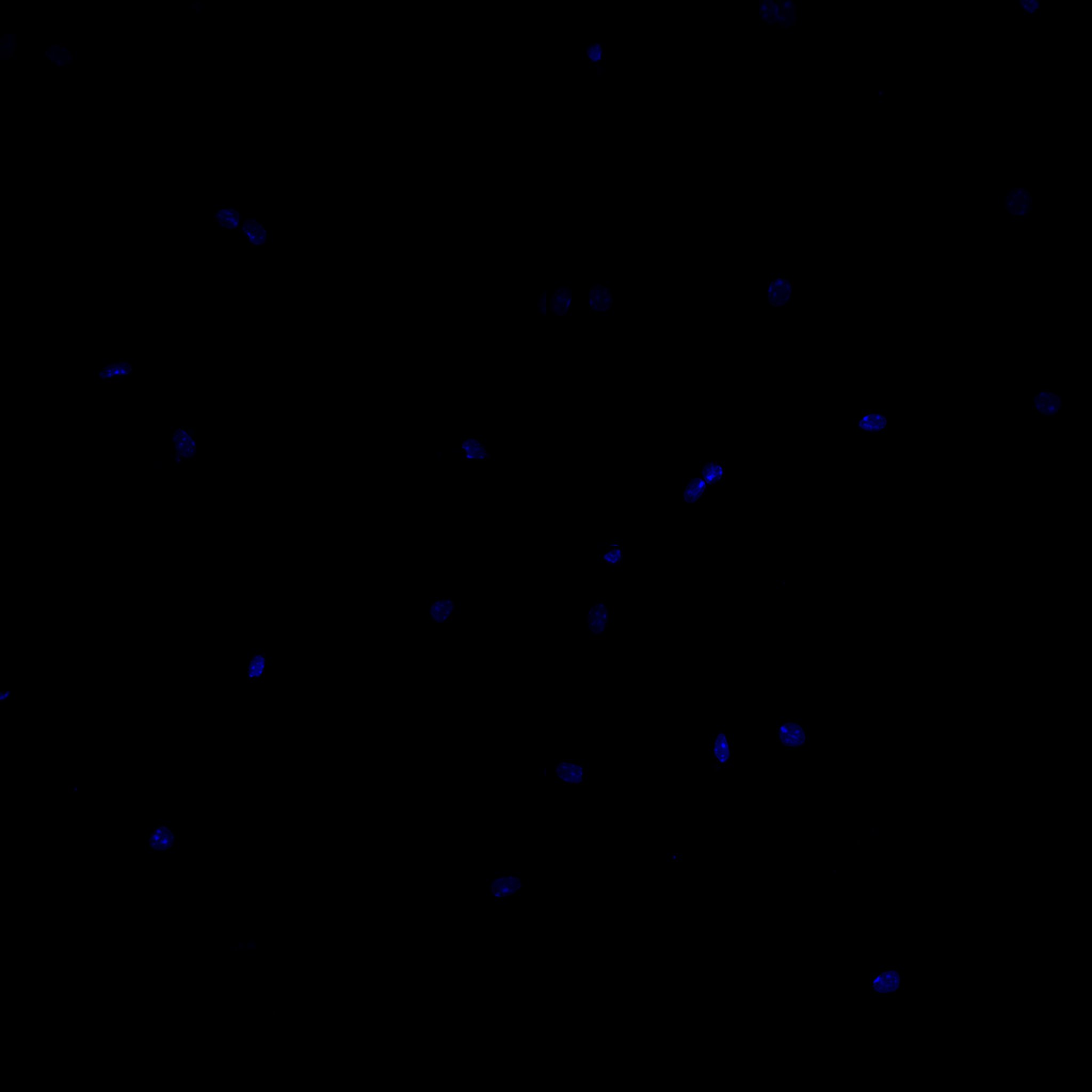

Supplement: Supplementary file 18 — Figure EV2C Source Data [file 44319_2025_673_MOESM18_ESM.zip › EV2C/EIPA+Hb/EIPA+Hb_hoechst staining.jpg]

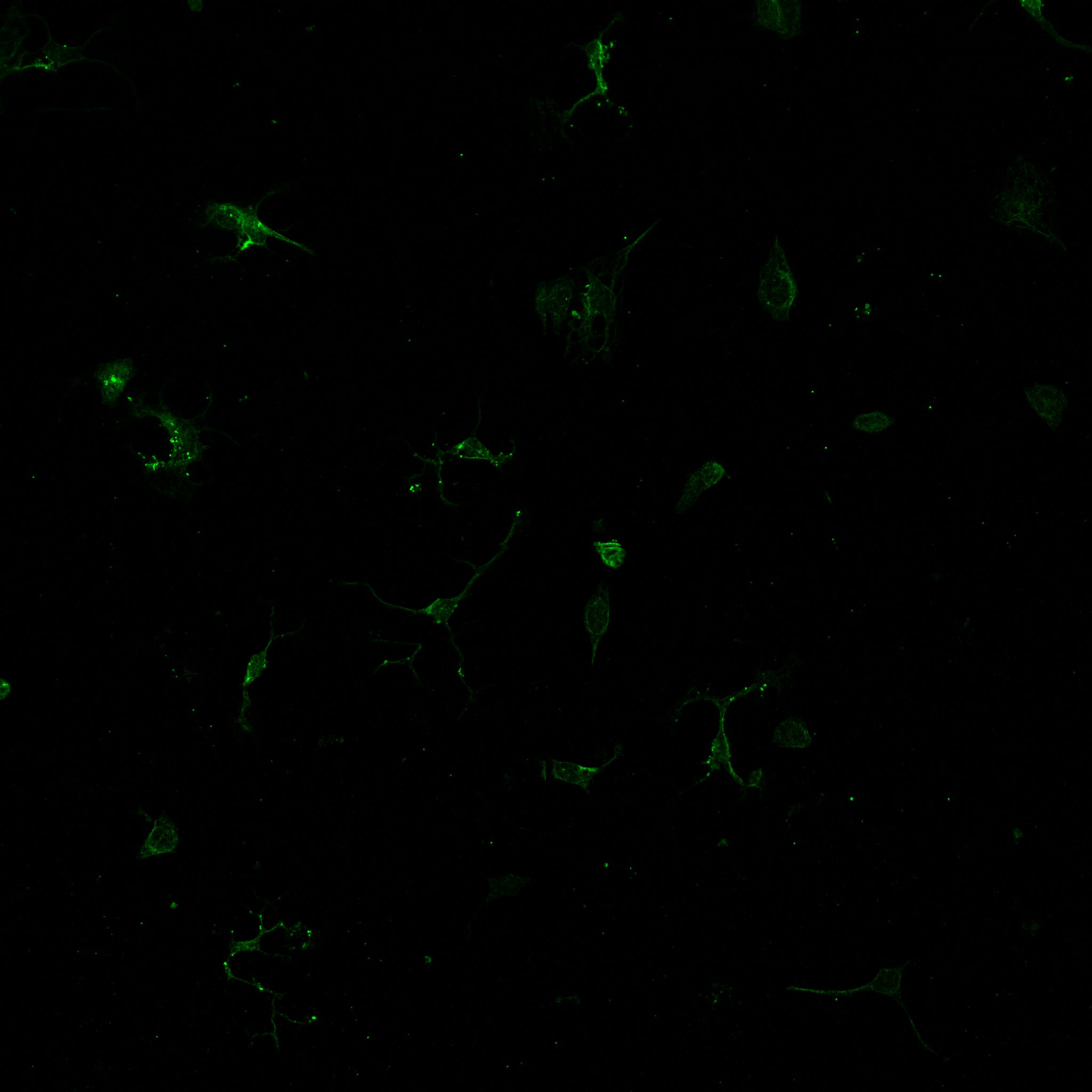

Supplement: Supplementary file 18 — Figure EV2C Source Data [file 44319_2025_673_MOESM18_ESM.zip › EV2C/EIPA+Hb/EIPA+Hb_Stab2 staining.jpg]

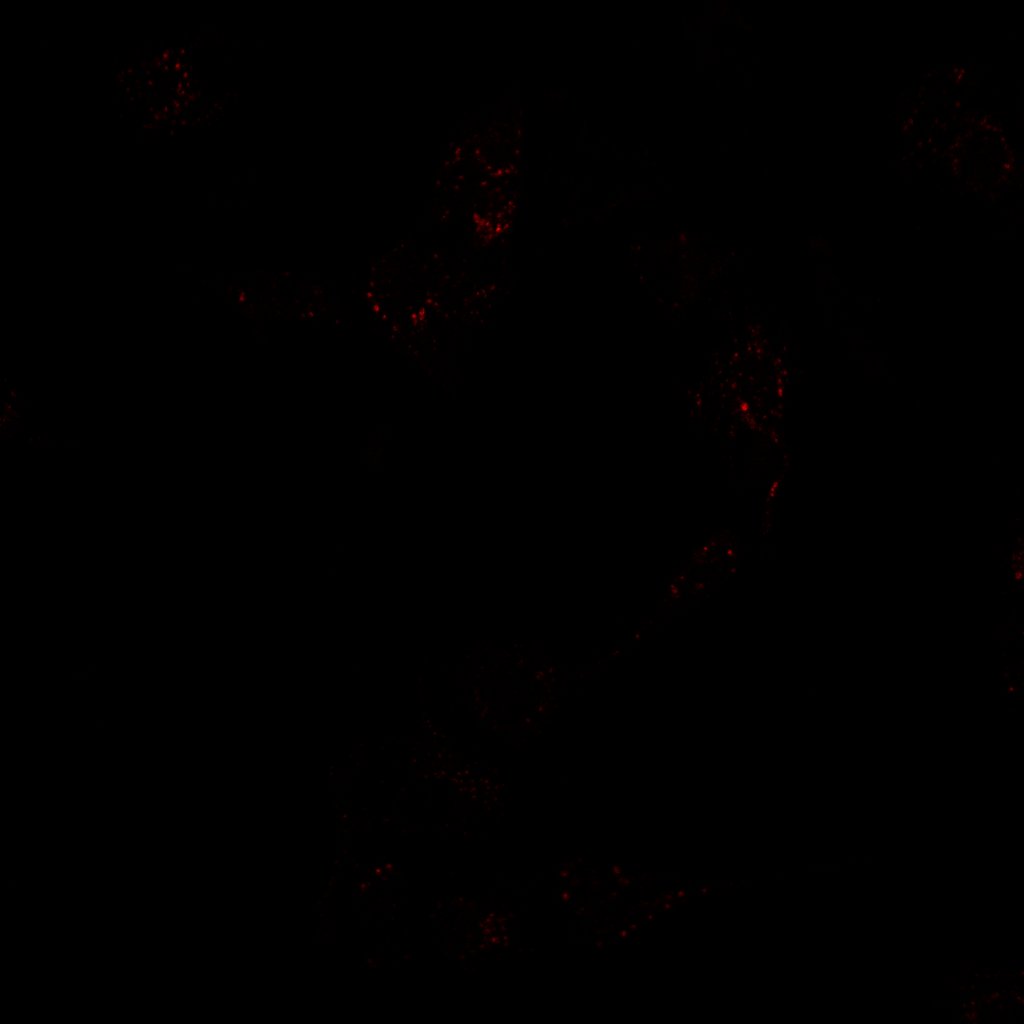

Supplement: Supplementary file 18 — Figure EV2C Source Data [file 44319_2025_673_MOESM18_ESM.zip › EV2C/Hb/Hb_ Hb.jpg]

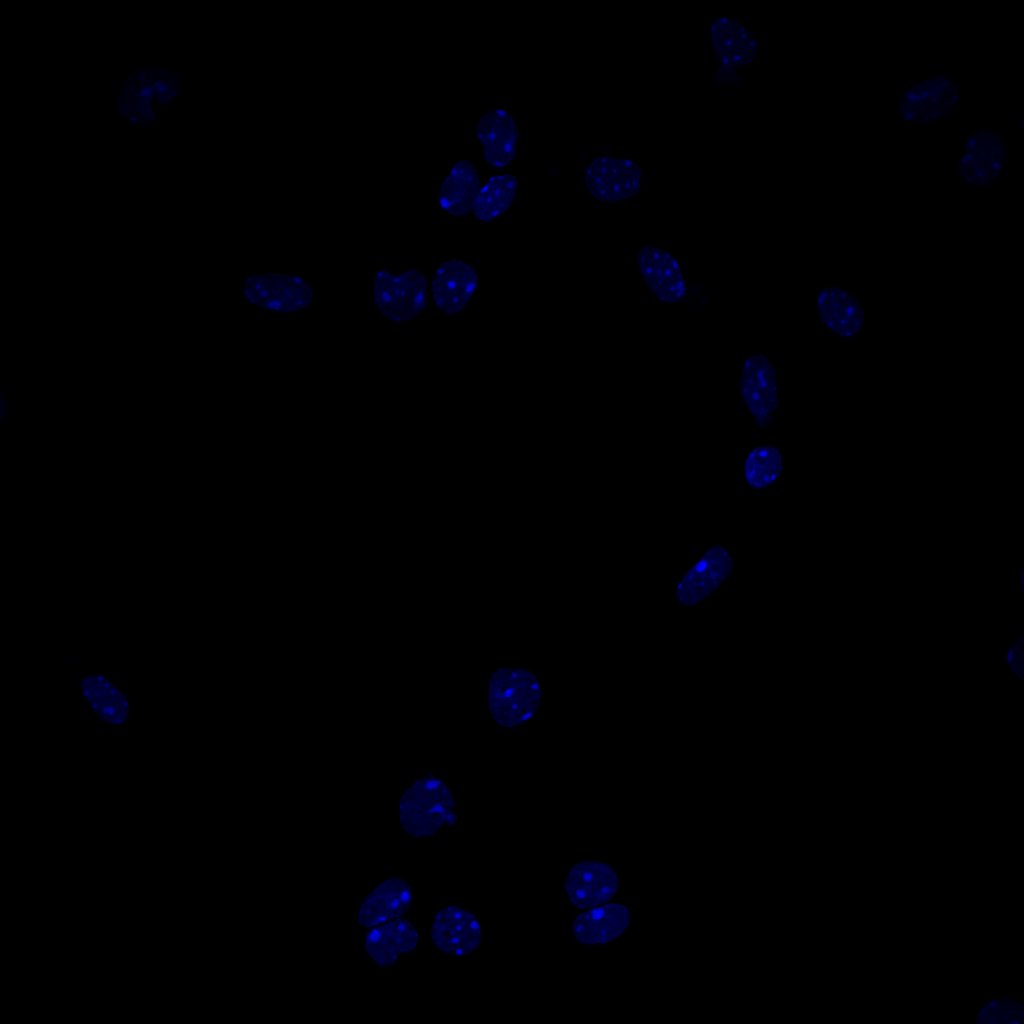

Supplement: Supplementary file 18 — Figure EV2C Source Data [file 44319_2025_673_MOESM18_ESM.zip › EV2C/Hb/Hb_hoechst staining.jpg]

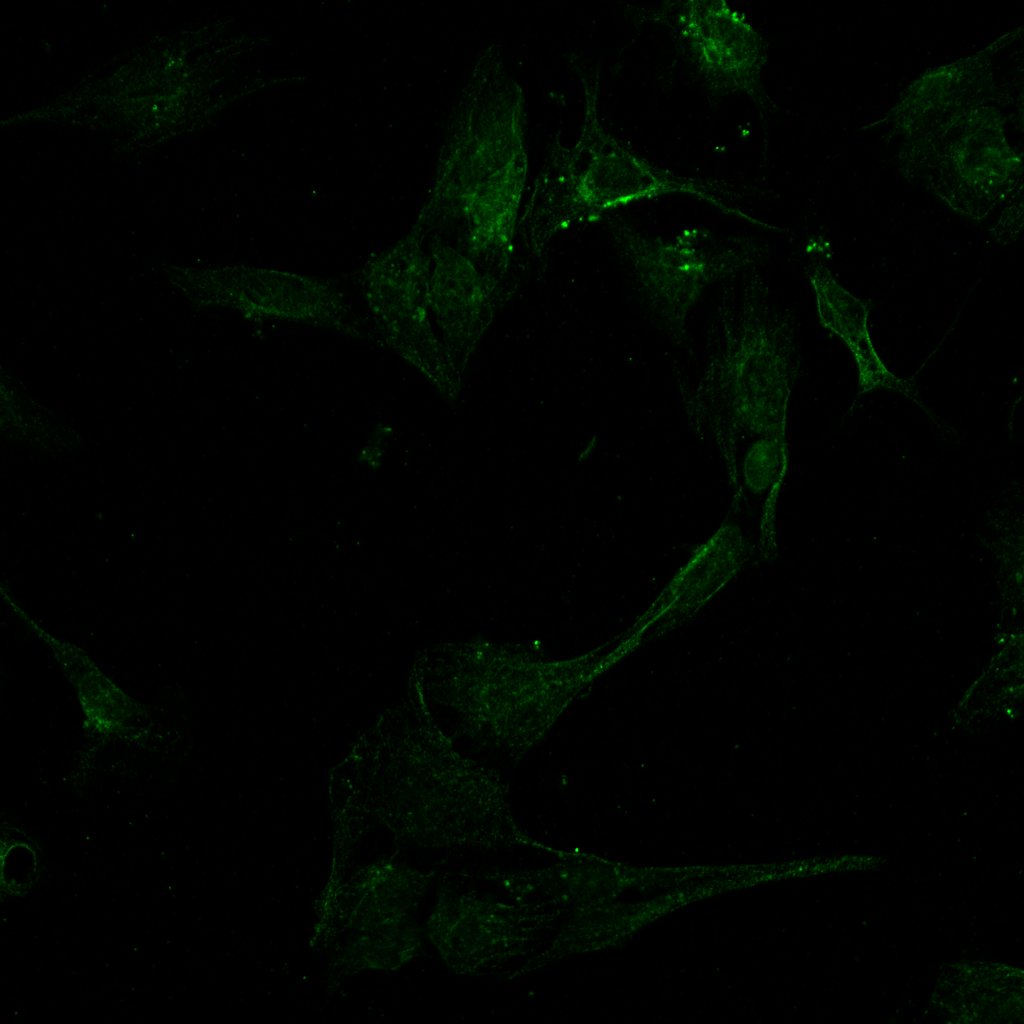

Supplement: Supplementary file 18 — Figure EV2C Source Data [file 44319_2025_673_MOESM18_ESM.zip › EV2C/Hb/Hb_Stab2 staining.jpg]

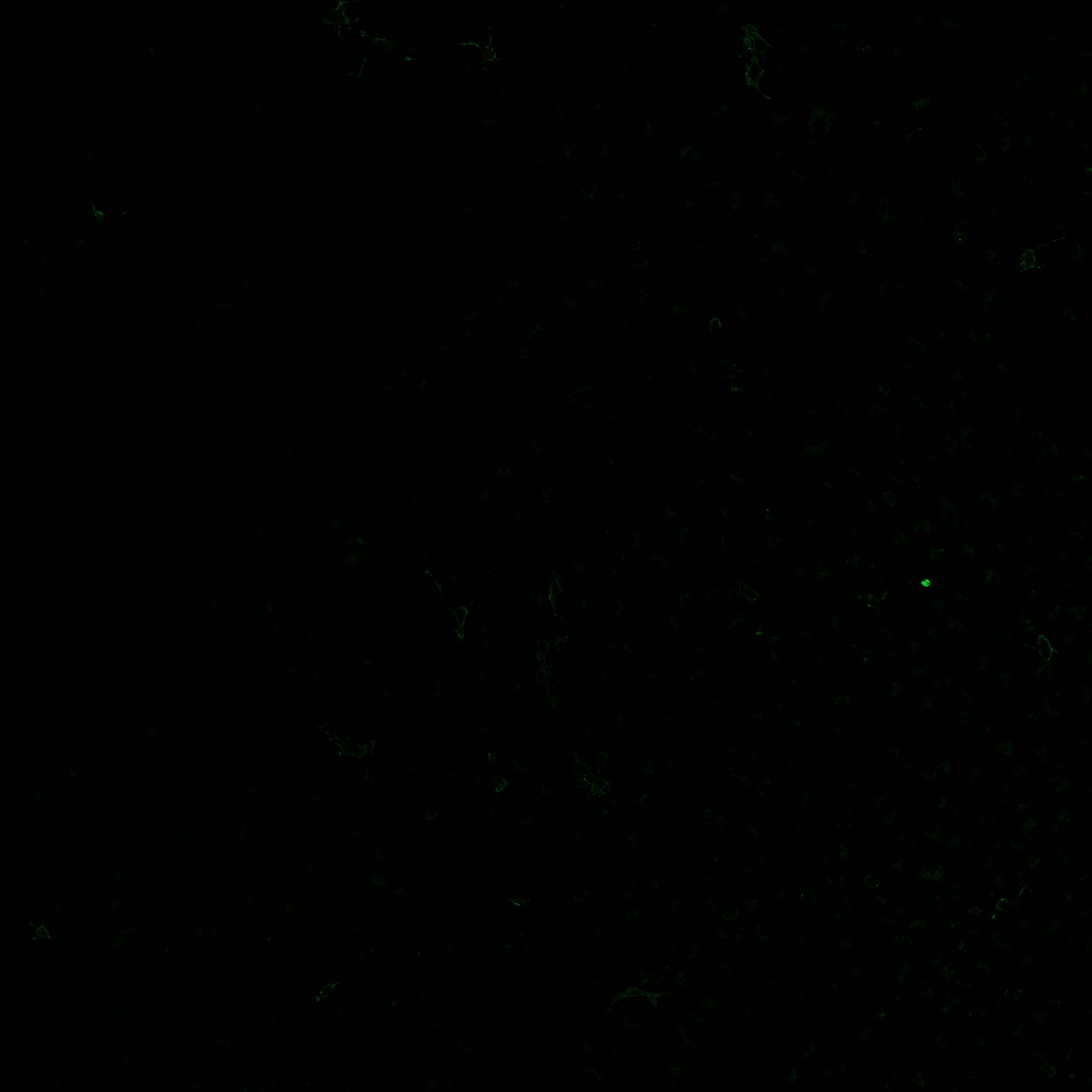

Supplement: Supplementary file 19 — Appendix Figure S3 Source Data [file 44319_2025_673_MOESM19_ESM.zip › Appendix Figure S3/KCs isolation/KCs isolation_CD31 staining.jpg]

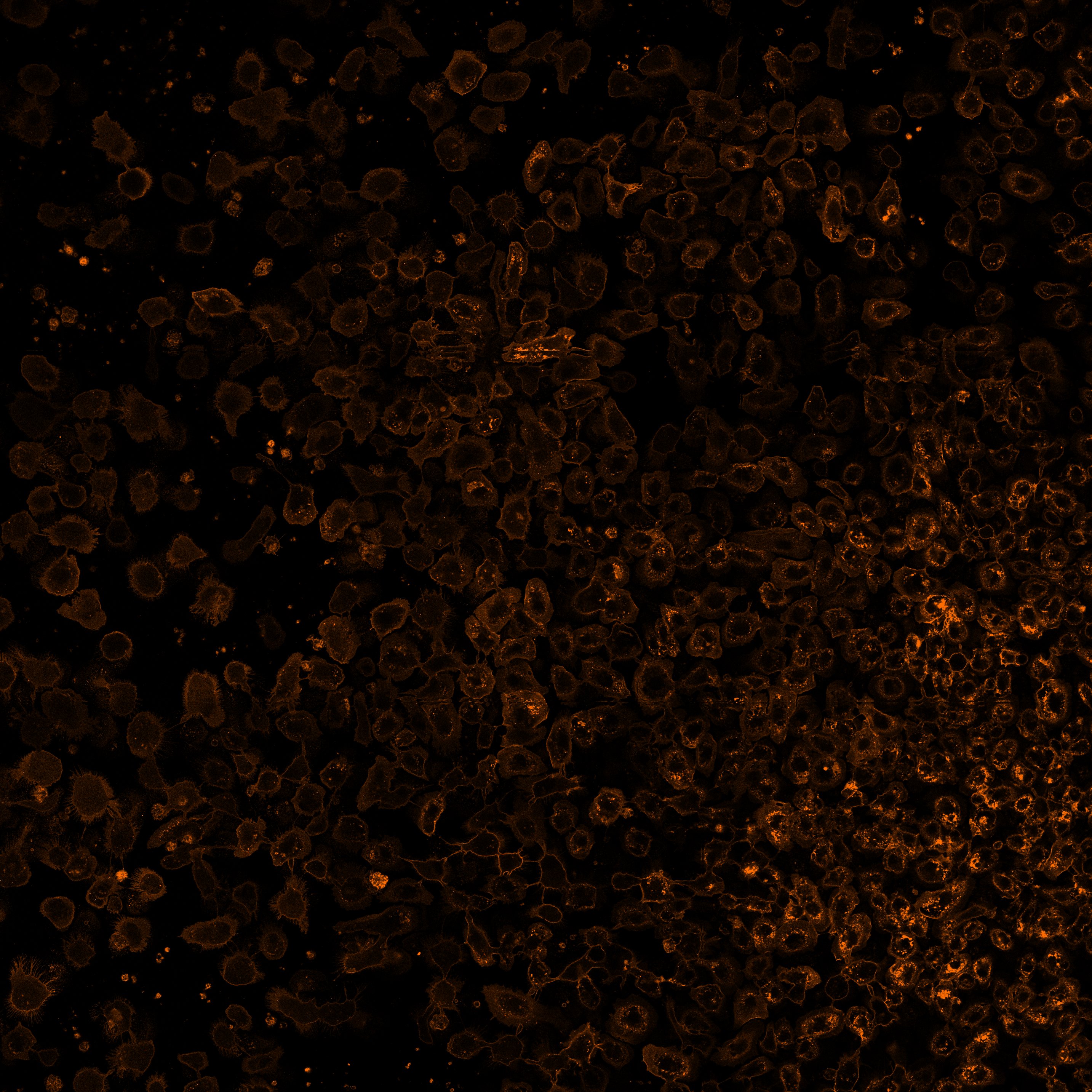

Supplement: Supplementary file 19 — Appendix Figure S3 Source Data [file 44319_2025_673_MOESM19_ESM.zip › Appendix Figure S3/KCs isolation/KCs isolation_F480 staining.jpg]

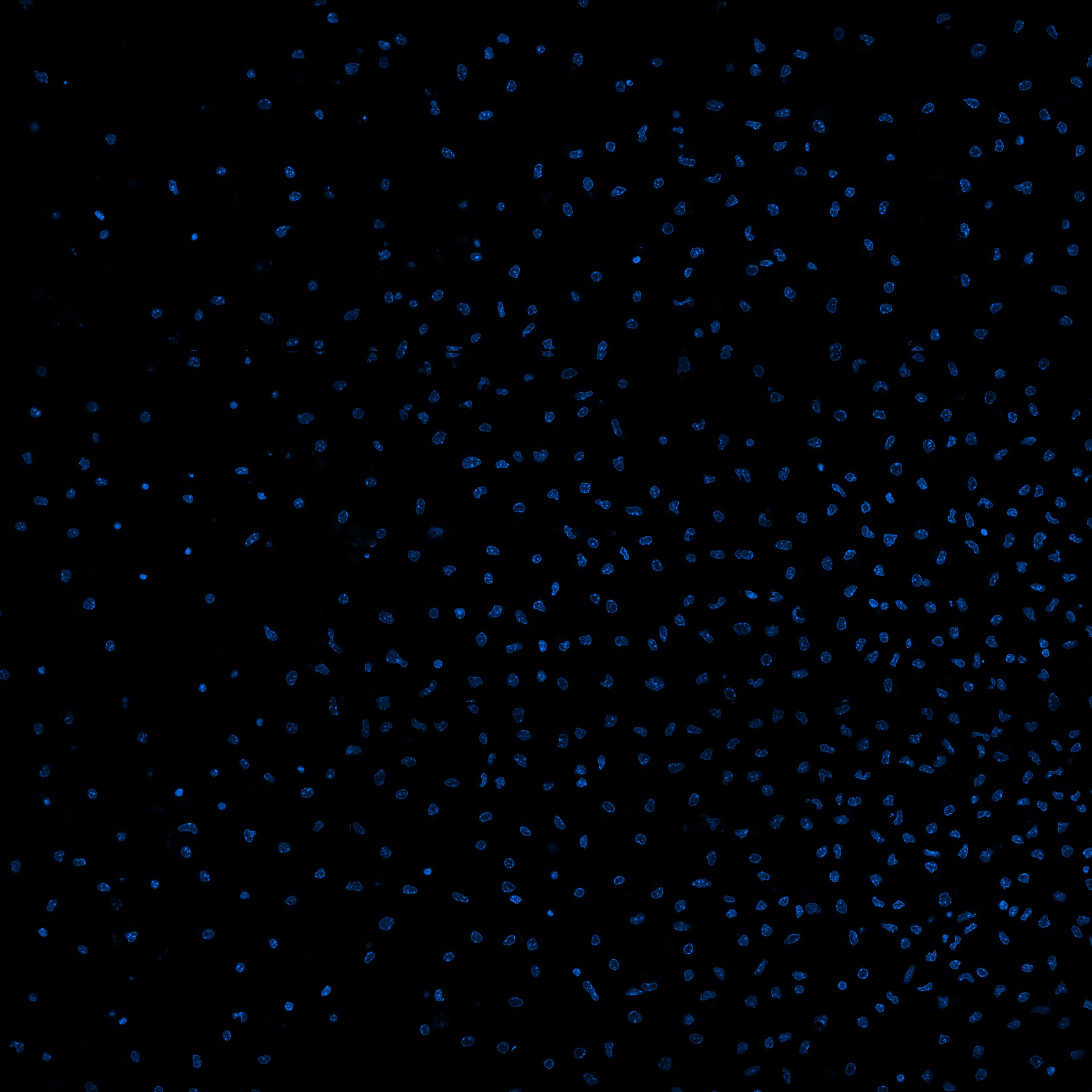

Supplement: Supplementary file 19 — Appendix Figure S3 Source Data [file 44319_2025_673_MOESM19_ESM.zip › Appendix Figure S3/KCs isolation/KCs isolation_hoechst staining.jpg]

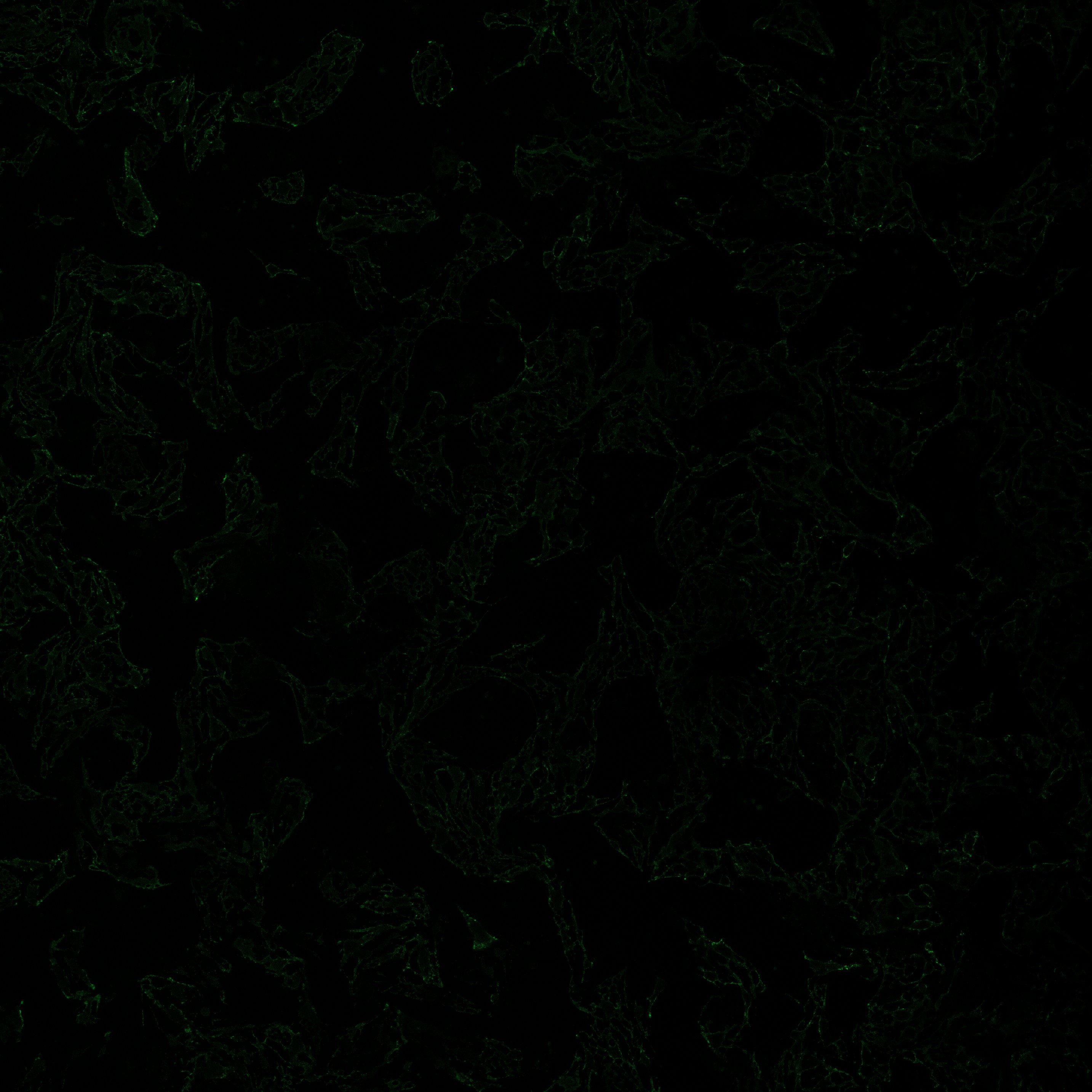

Supplement: Supplementary file 19 — Appendix Figure S3 Source Data [file 44319_2025_673_MOESM19_ESM.zip › Appendix Figure S3/LSECs isolation/LSECs isolation_CD31 staining.jpg]

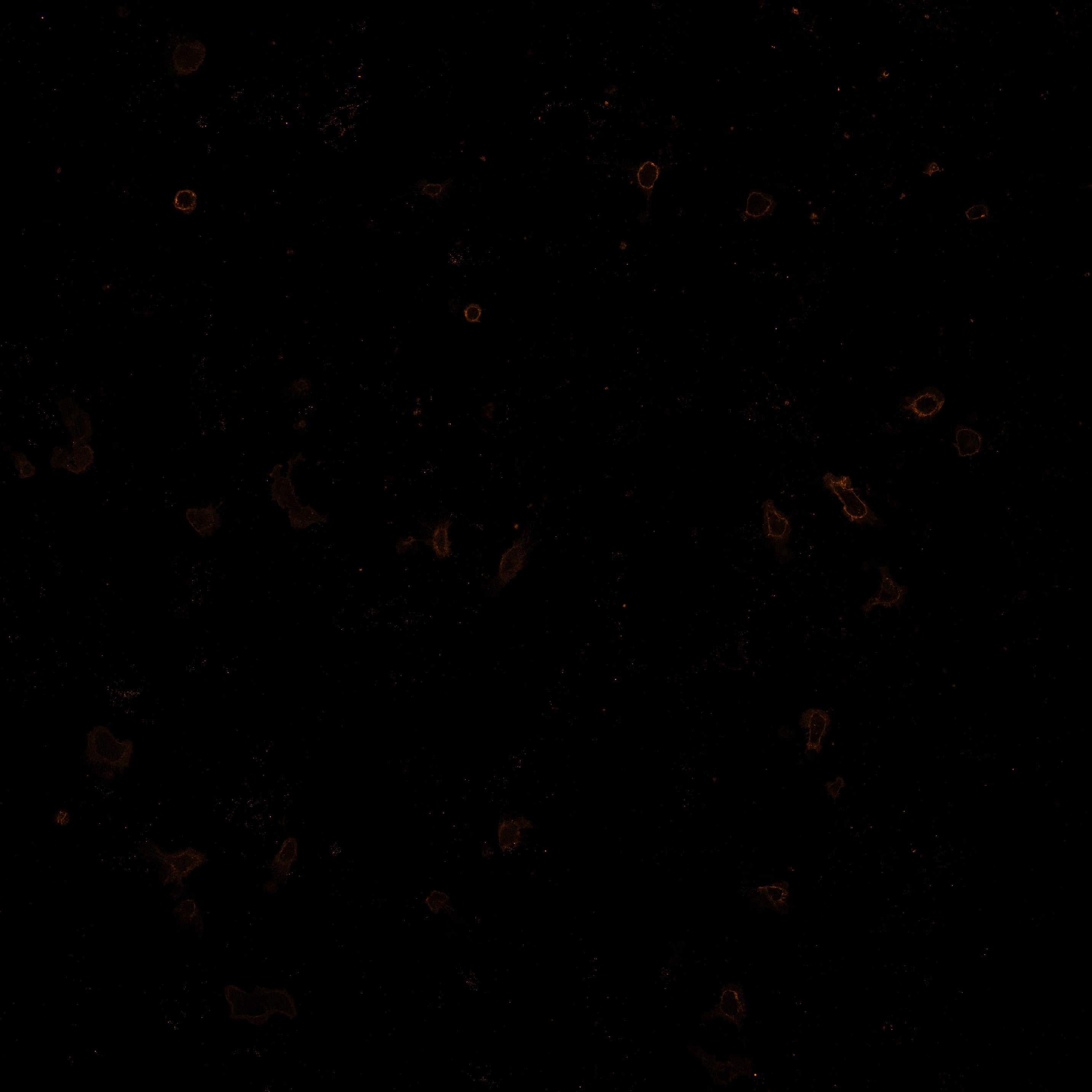

Supplement: Supplementary file 19 — Appendix Figure S3 Source Data [file 44319_2025_673_MOESM19_ESM.zip › Appendix Figure S3/LSECs isolation/LSECs isolation_F480 staining.jpg]

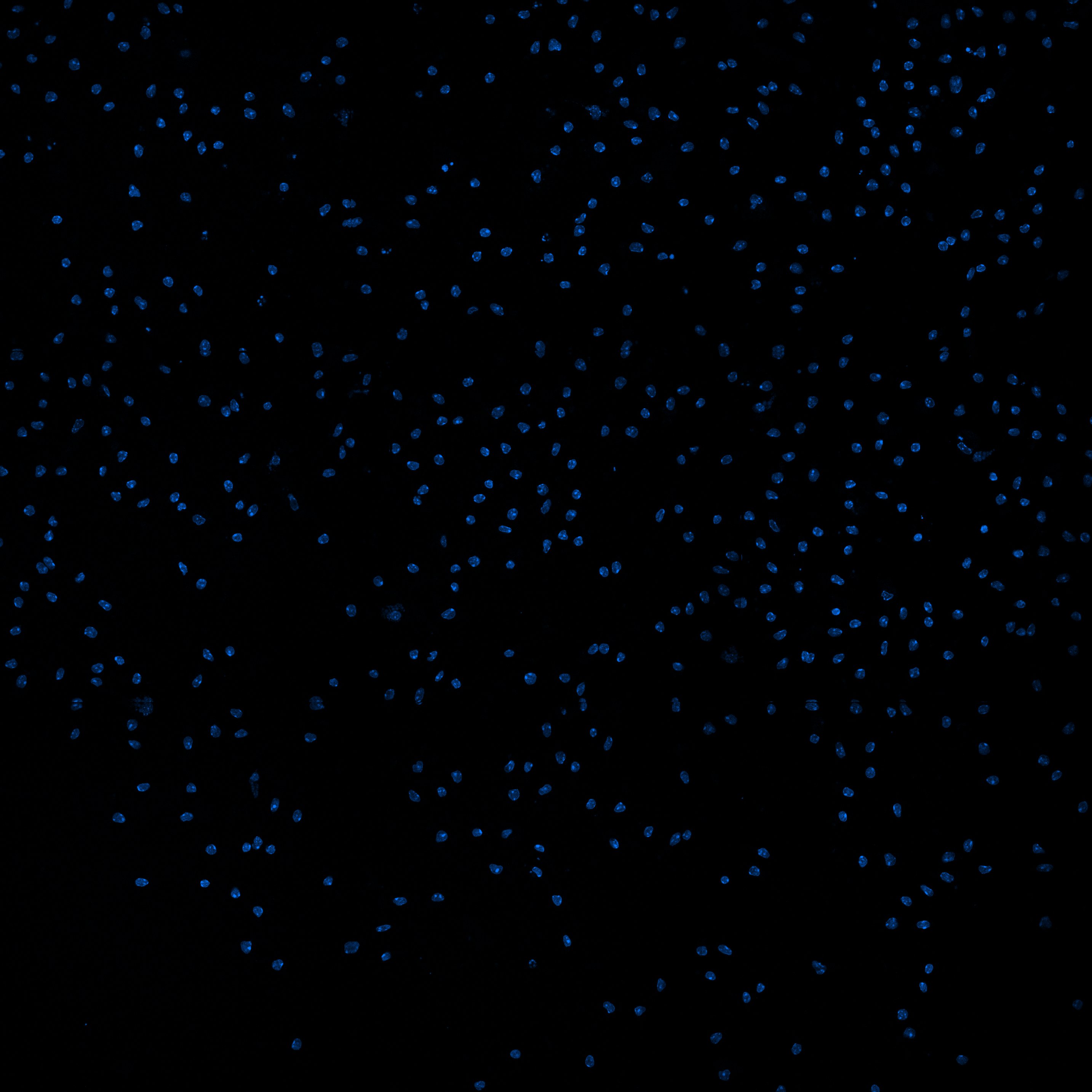

Supplement: Supplementary file 19 — Appendix Figure S3 Source Data [file 44319_2025_673_MOESM19_ESM.zip › Appendix Figure S3/LSECs isolation/LSECs isolation_hoechst staining.jpg]

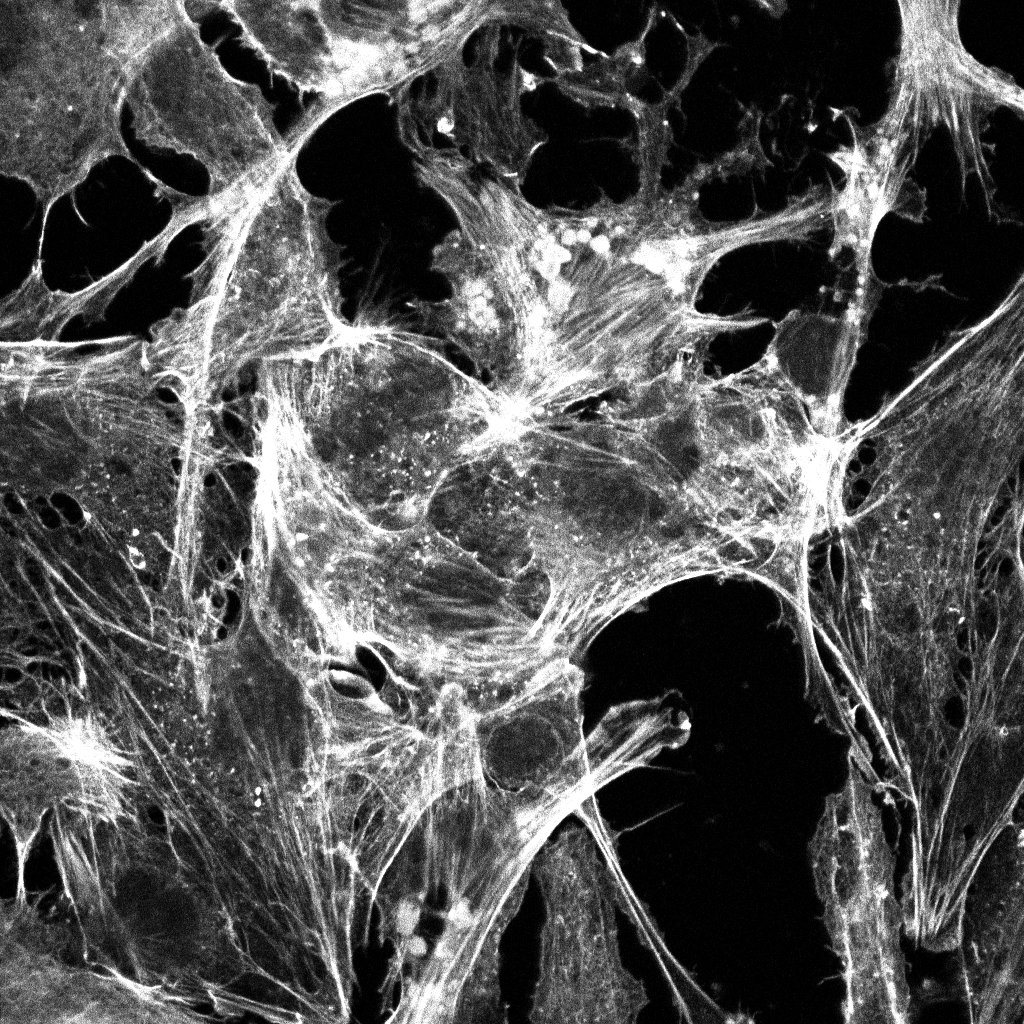

Supplement: Supplementary file 21 — Figure EV2A Source Data [file 44319_2025_673_MOESM21_ESM.zip › EV2A/control_actin_grey.tif]

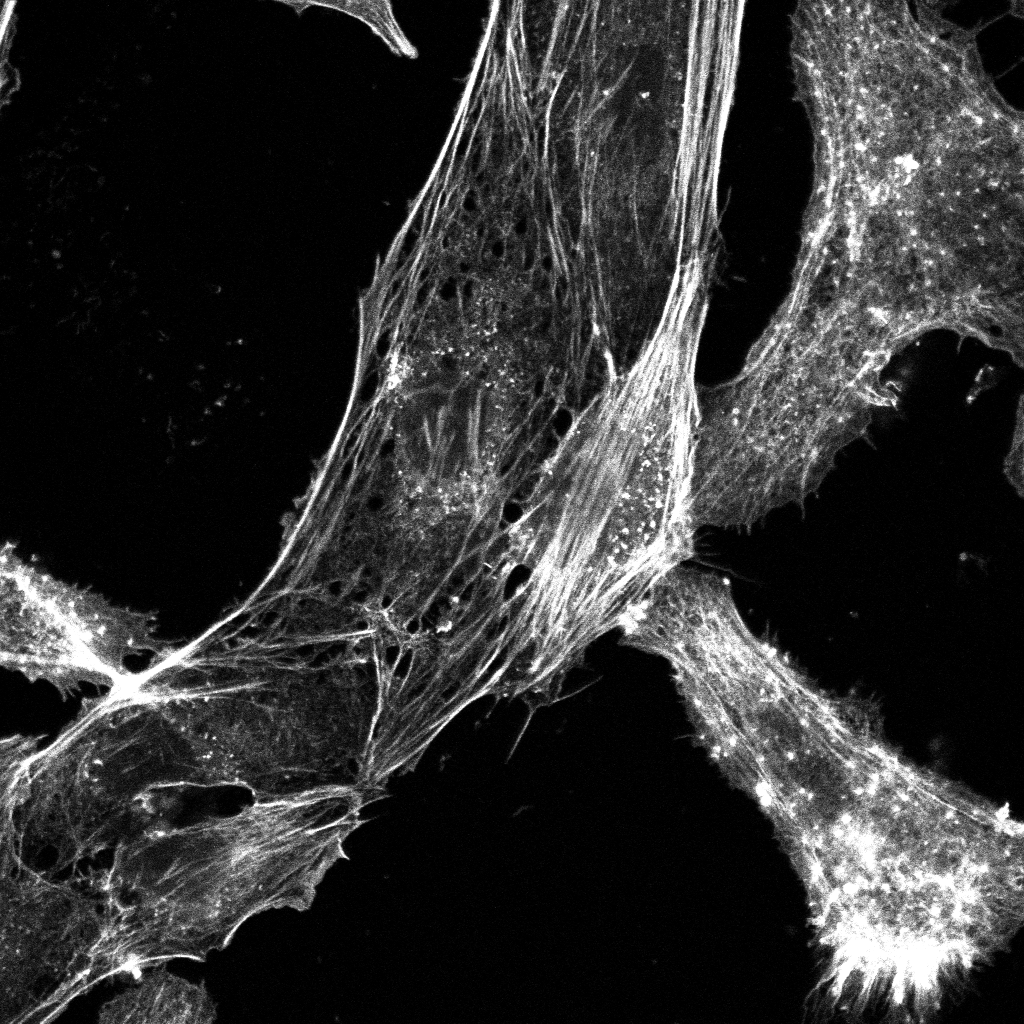

Supplement: Supplementary file 21 — Figure EV2A Source Data [file 44319_2025_673_MOESM21_ESM.zip › EV2A/Hb 30 min_actin_grey.tif]

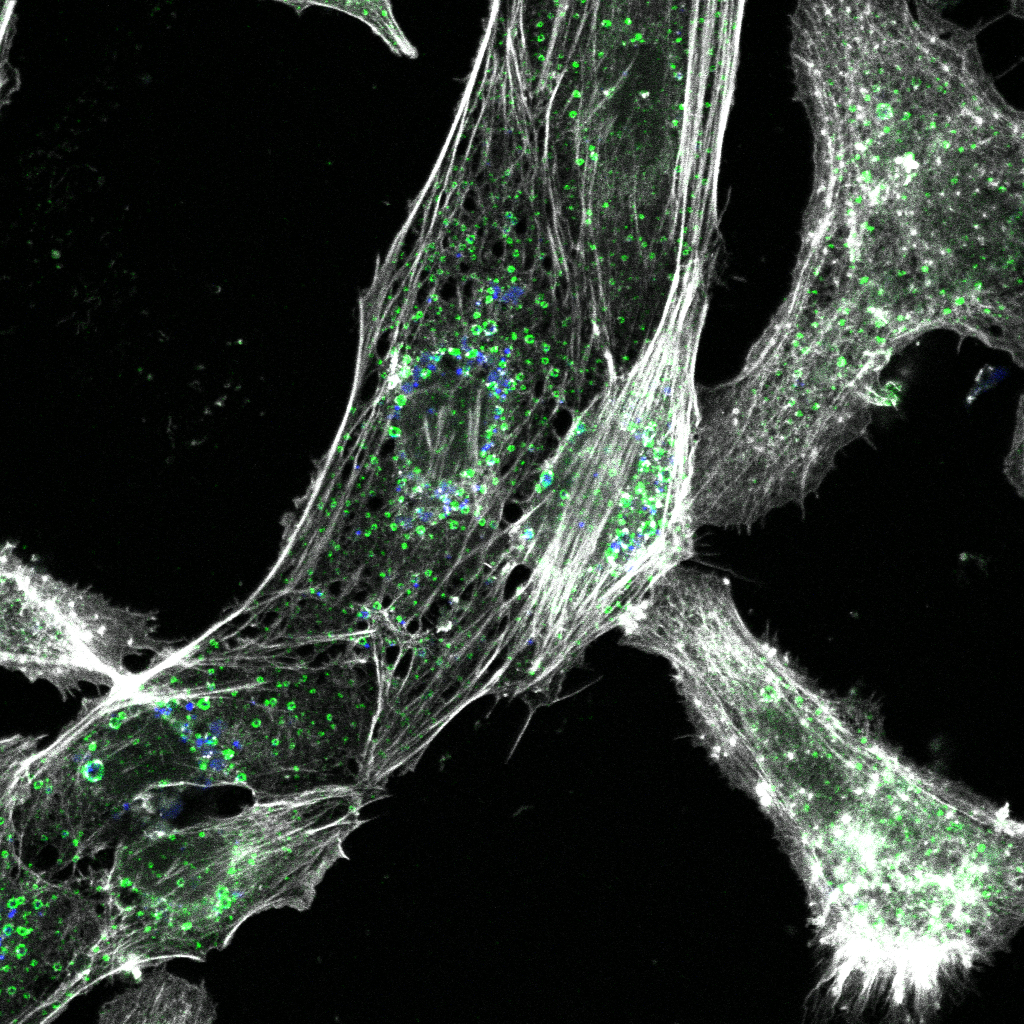

Supplement: Supplementary file 21 — Figure EV2A Source Data [file 44319_2025_673_MOESM21_ESM.zip › EV2A/Hb 30 min_merged.tif]

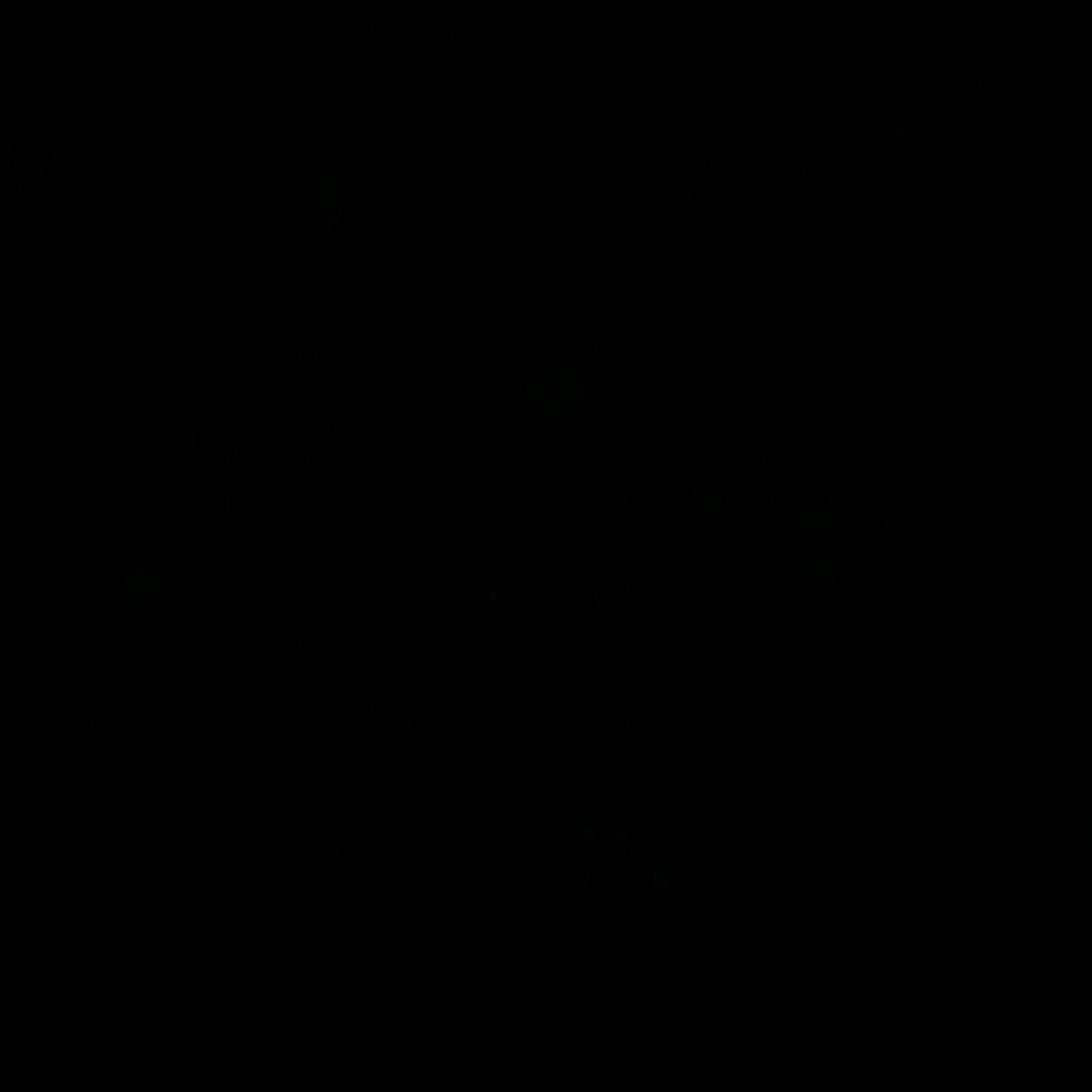

Supplement: Supplementary file 22 — Figure EV2B Source Data [file 44319_2025_673_MOESM22_ESM.zip › EV2B/Control/control_dextran.jpg]

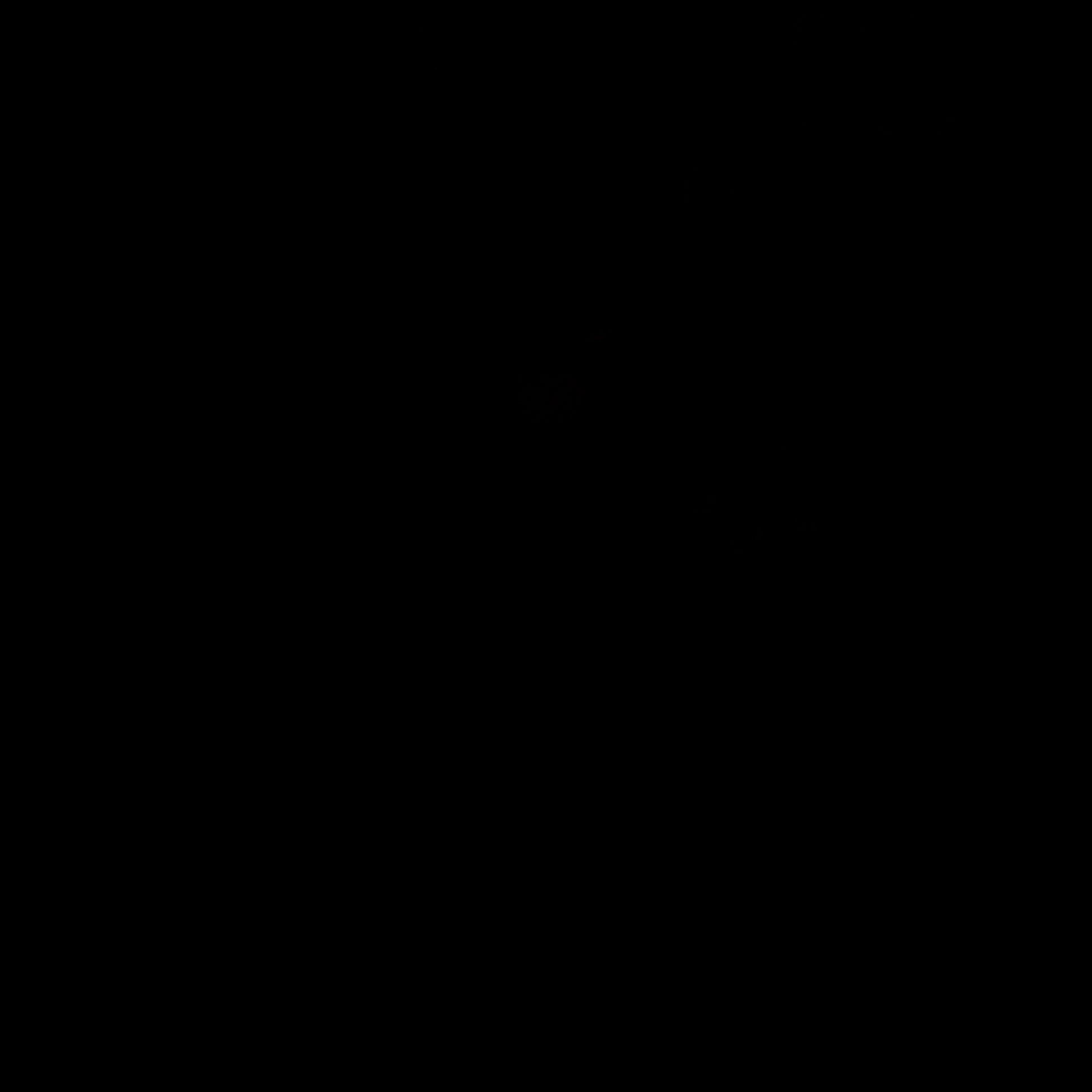

Supplement: Supplementary file 22 — Figure EV2B Source Data [file 44319_2025_673_MOESM22_ESM.zip › EV2B/Control/control_Hb.jpg]

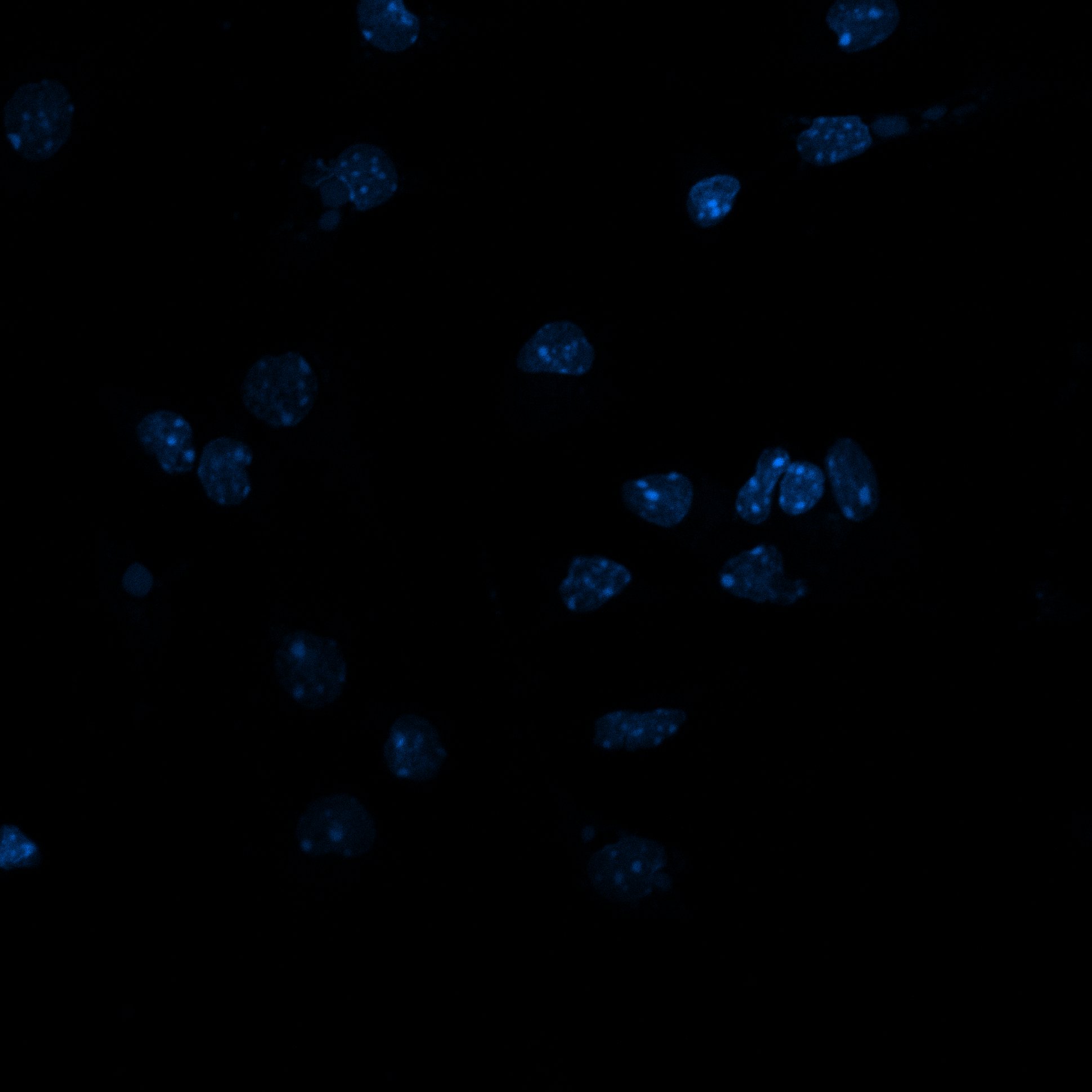

Supplement: Supplementary file 22 — Figure EV2B Source Data [file 44319_2025_673_MOESM22_ESM.zip › EV2B/Control/control_hoechst staining.jpg]

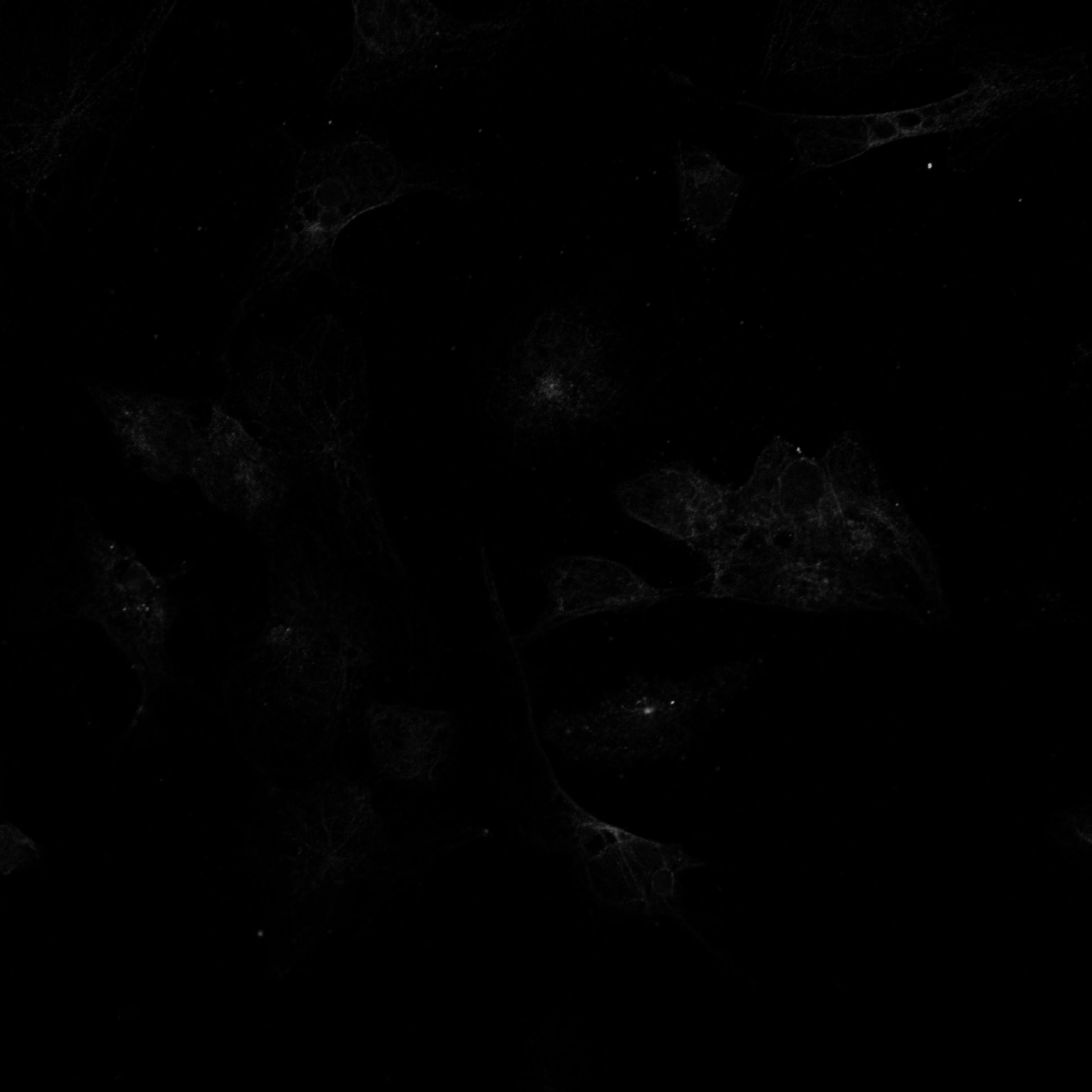

Supplement: Supplementary file 22 — Figure EV2B Source Data [file 44319_2025_673_MOESM22_ESM.zip › EV2B/Control/control_Stab2 staining.jpg]

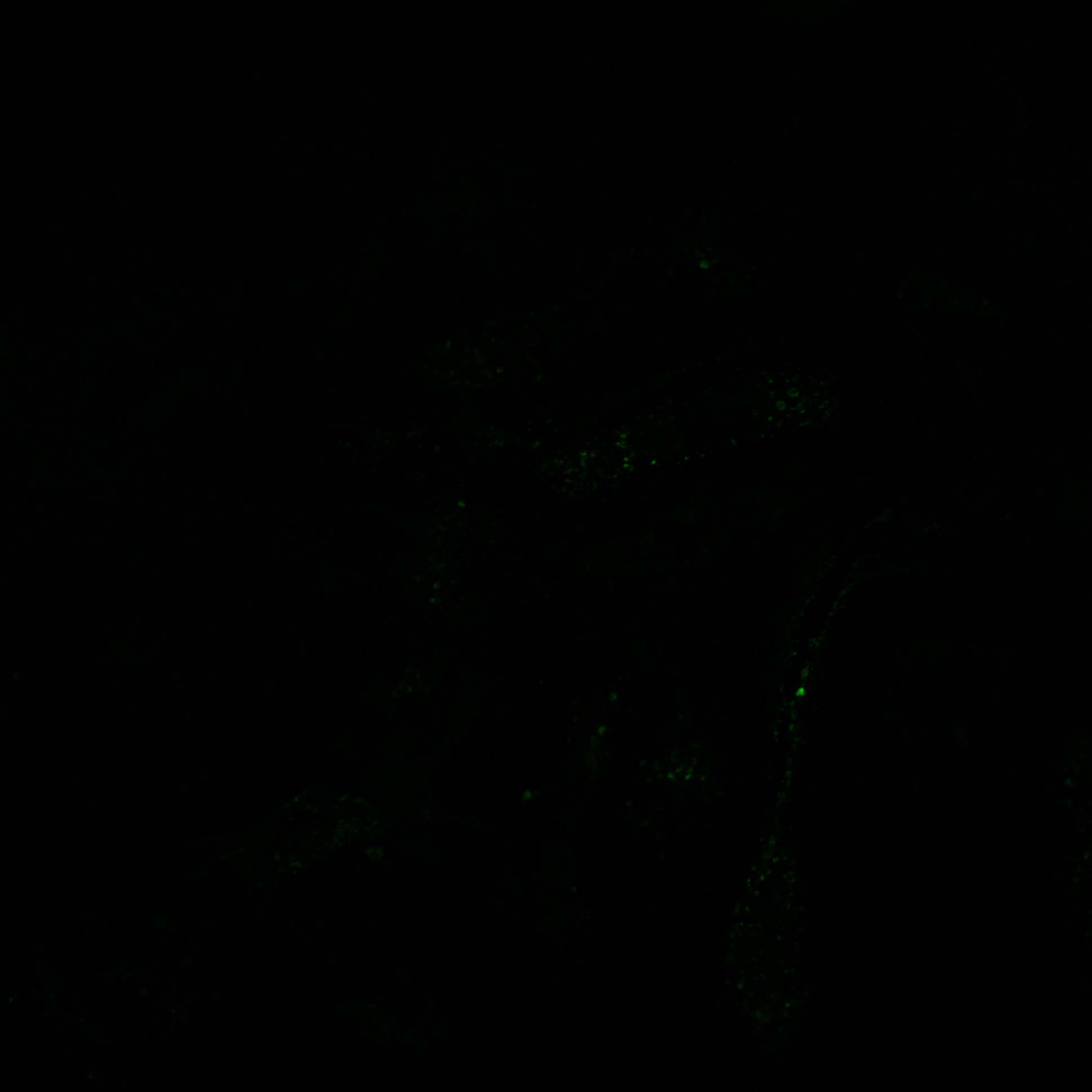

Supplement: Supplementary file 22 — Figure EV2B Source Data [file 44319_2025_673_MOESM22_ESM.zip › EV2B/Dextran + Hb/Dextran+Hb_ Dextran.jpg]

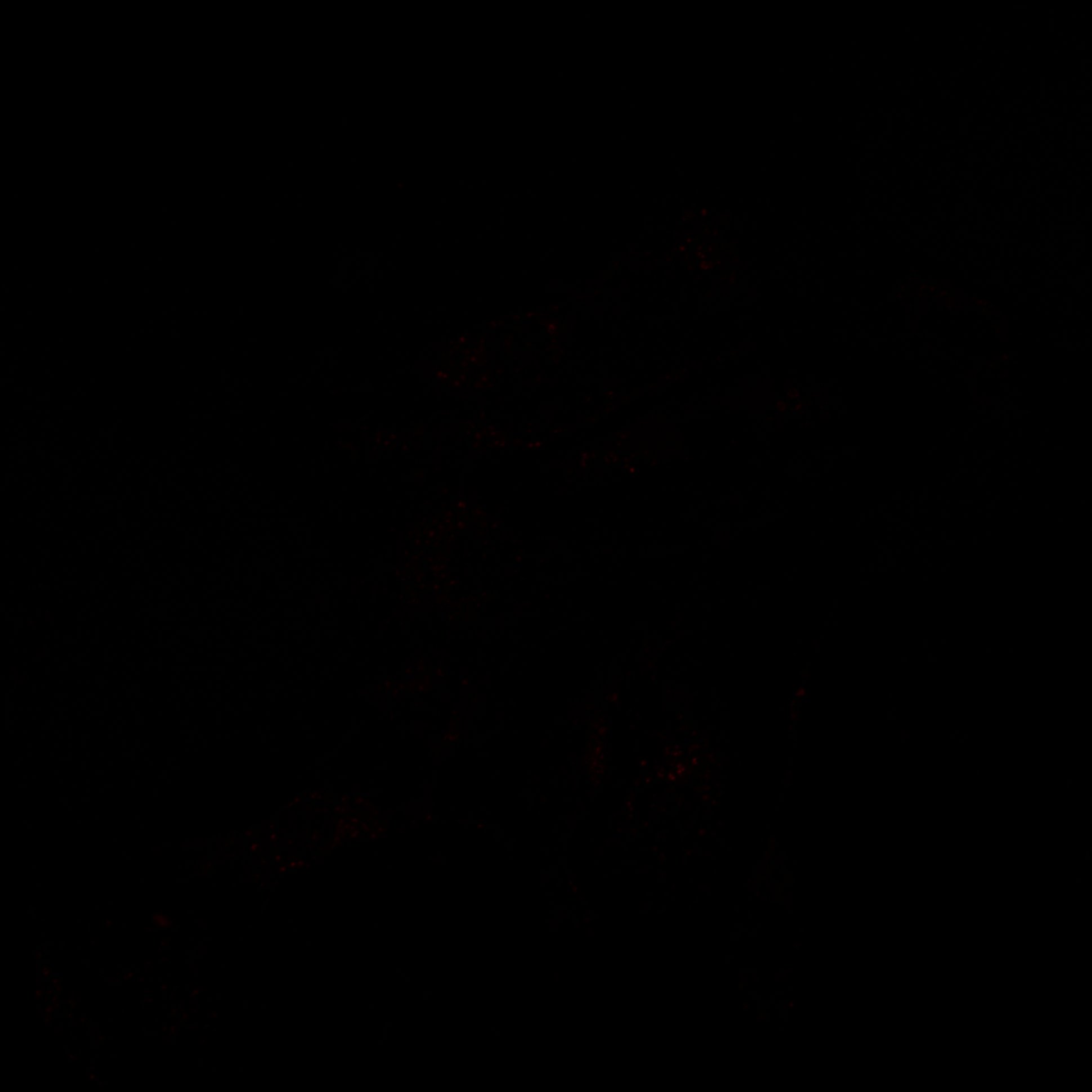

Supplement: Supplementary file 22 — Figure EV2B Source Data [file 44319_2025_673_MOESM22_ESM.zip › EV2B/Dextran + Hb/Dextran+Hb_ Hb.jpg]

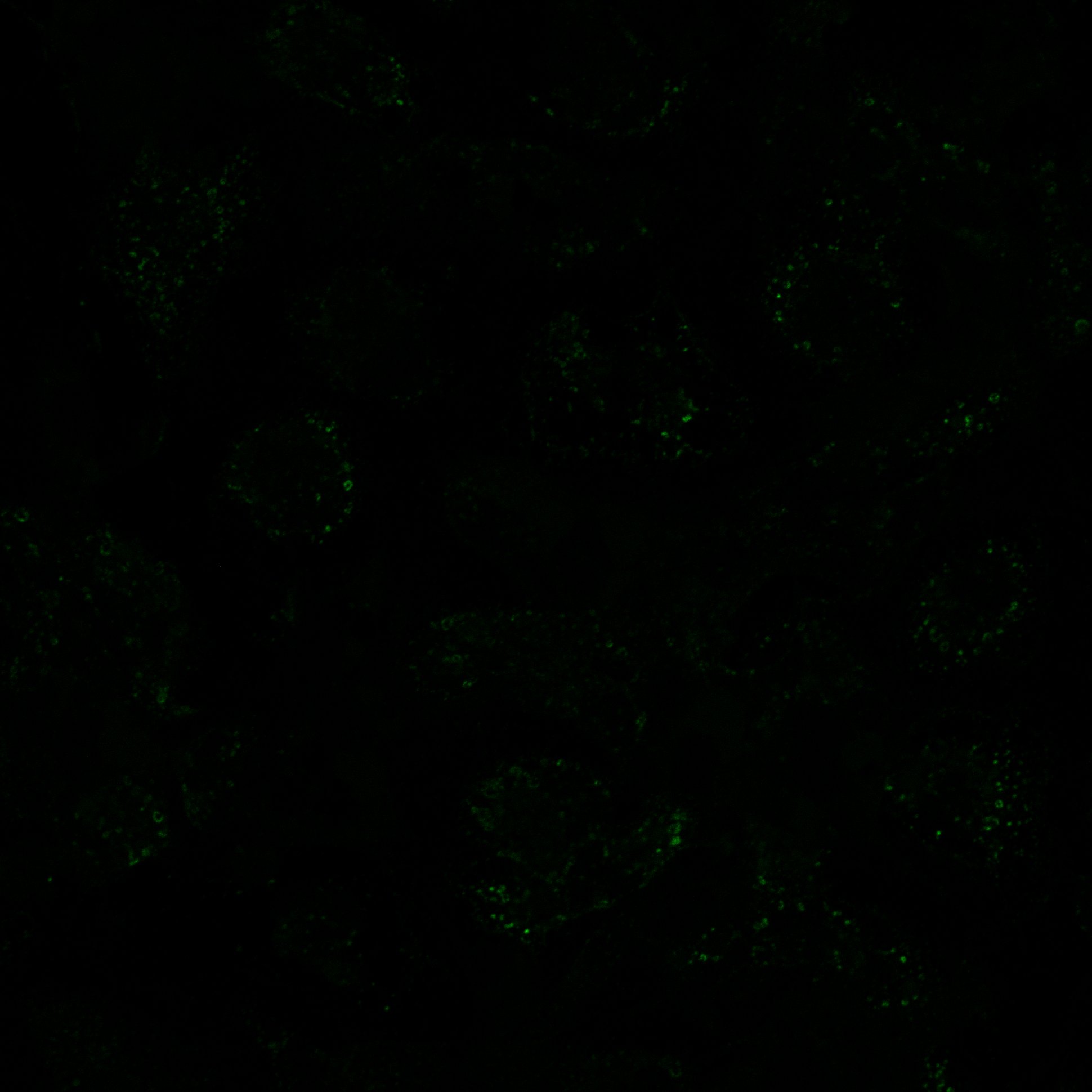

Supplement: Supplementary file 22 — Figure EV2B Source Data [file 44319_2025_673_MOESM22_ESM.zip › EV2B/Dextran/Dextran_Dextran.jpg]

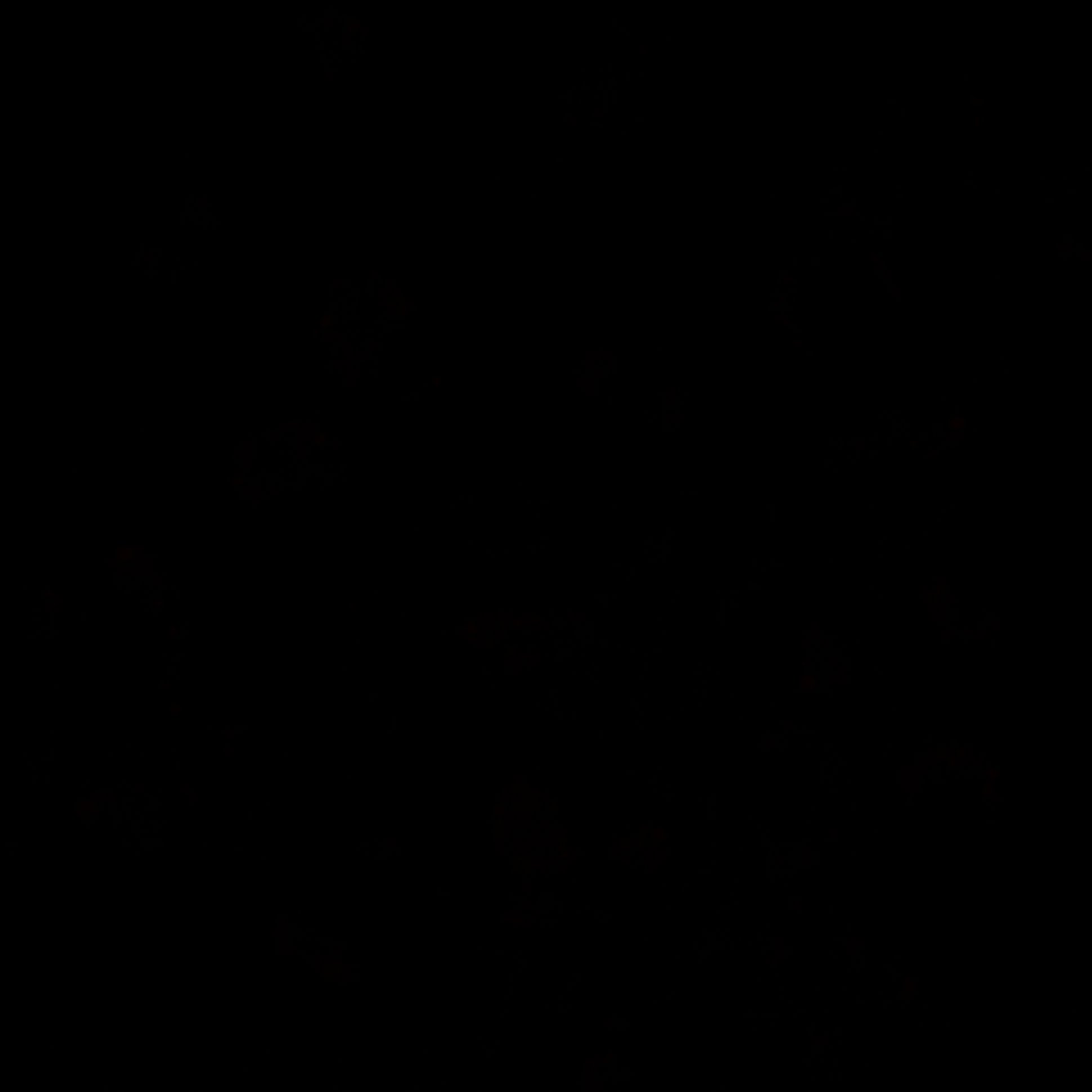

Supplement: Supplementary file 22 — Figure EV2B Source Data [file 44319_2025_673_MOESM22_ESM.zip › EV2B/Dextran/Dextran_Hb.jpg]

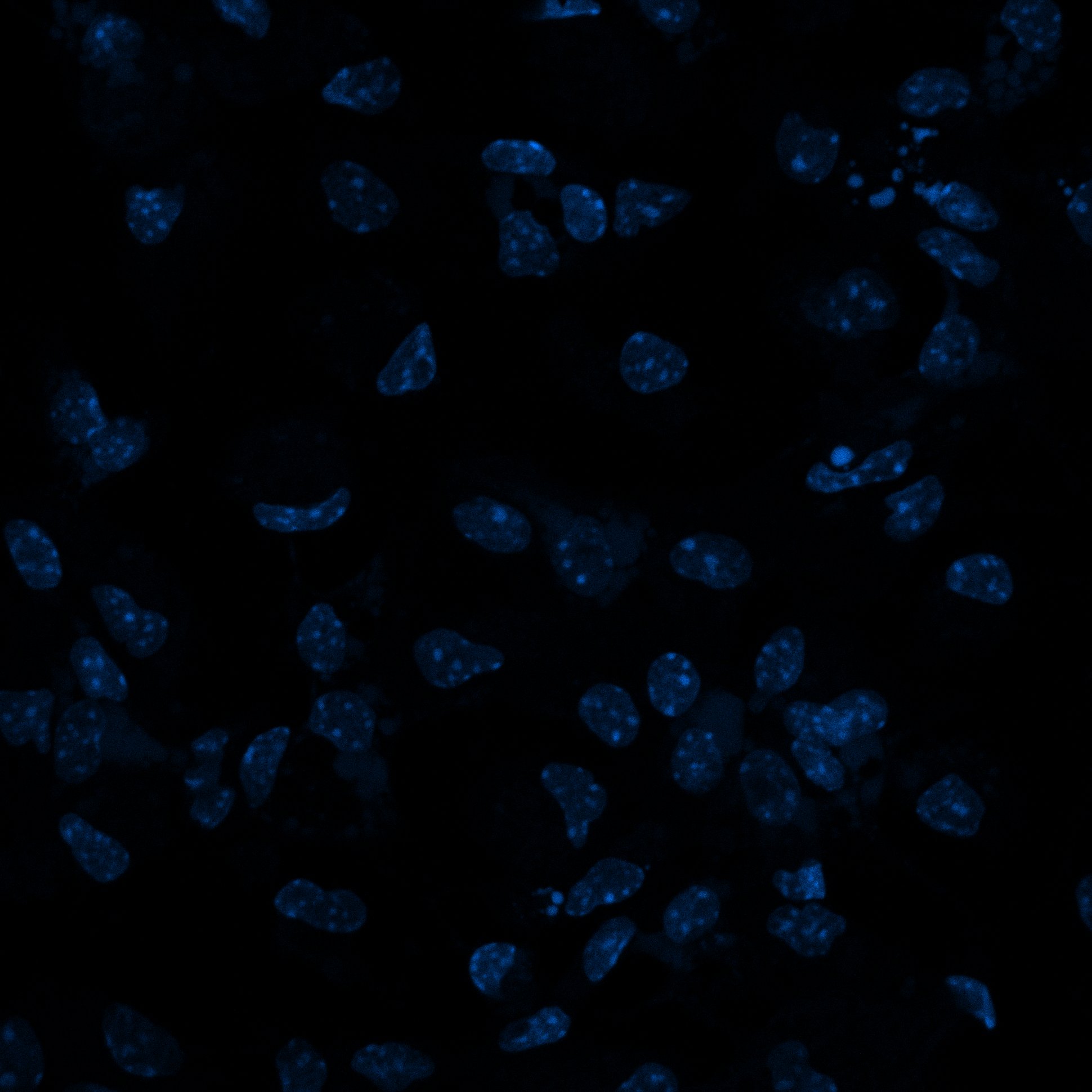

Supplement: Supplementary file 22 — Figure EV2B Source Data [file 44319_2025_673_MOESM22_ESM.zip › EV2B/Dextran/Dextran_hoechst staining.jpg]

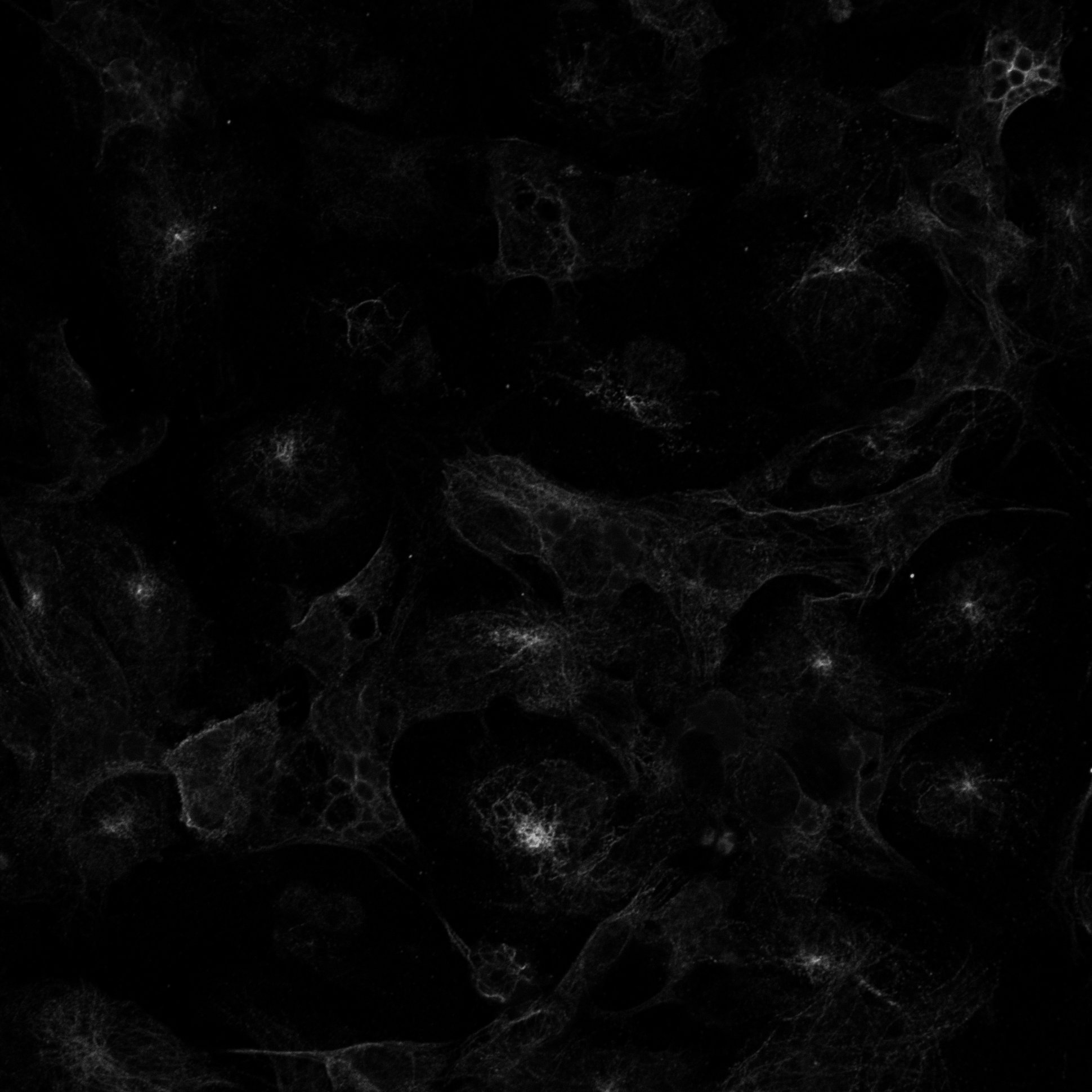

Supplement: Supplementary file 22 — Figure EV2B Source Data [file 44319_2025_673_MOESM22_ESM.zip › EV2B/Dextran/Dextran_Stab2 staining.jpg]

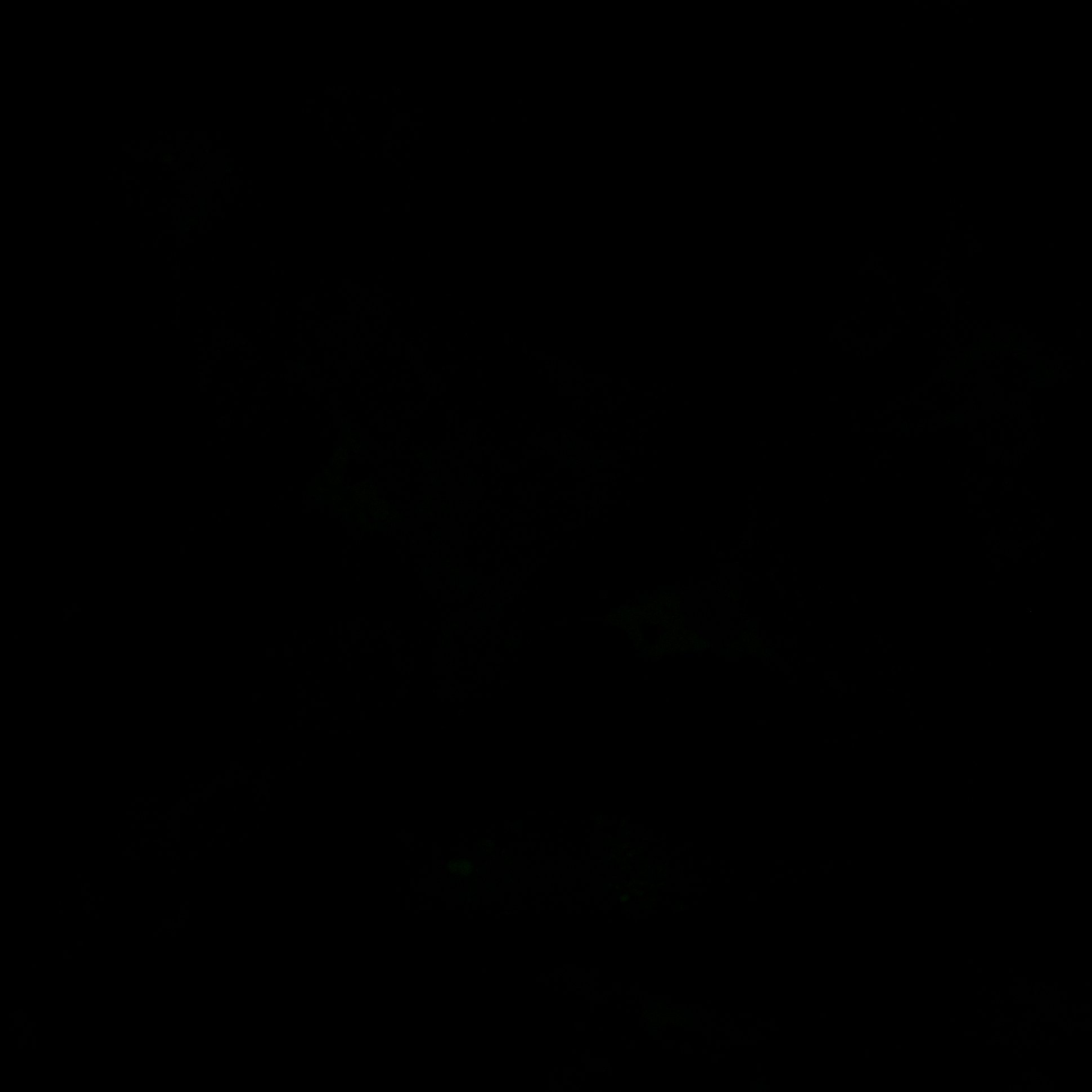

Supplement: Supplementary file 22 — Figure EV2B Source Data [file 44319_2025_673_MOESM22_ESM.zip › EV2B/Hb/Hb_Dextran.jpg]

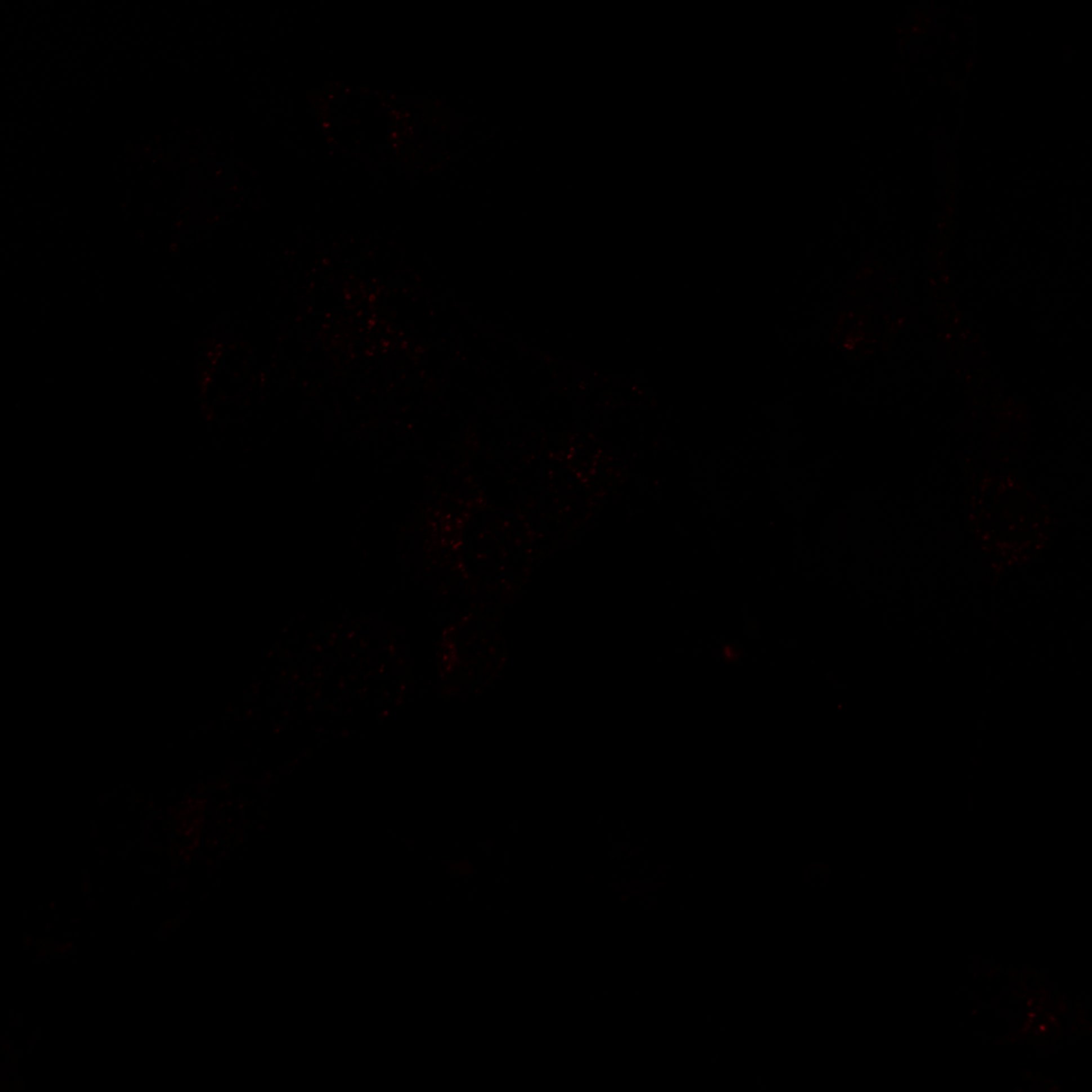

Supplement: Supplementary file 22 — Figure EV2B Source Data [file 44319_2025_673_MOESM22_ESM.zip › EV2B/Hb/Hb_Hb.jpg]

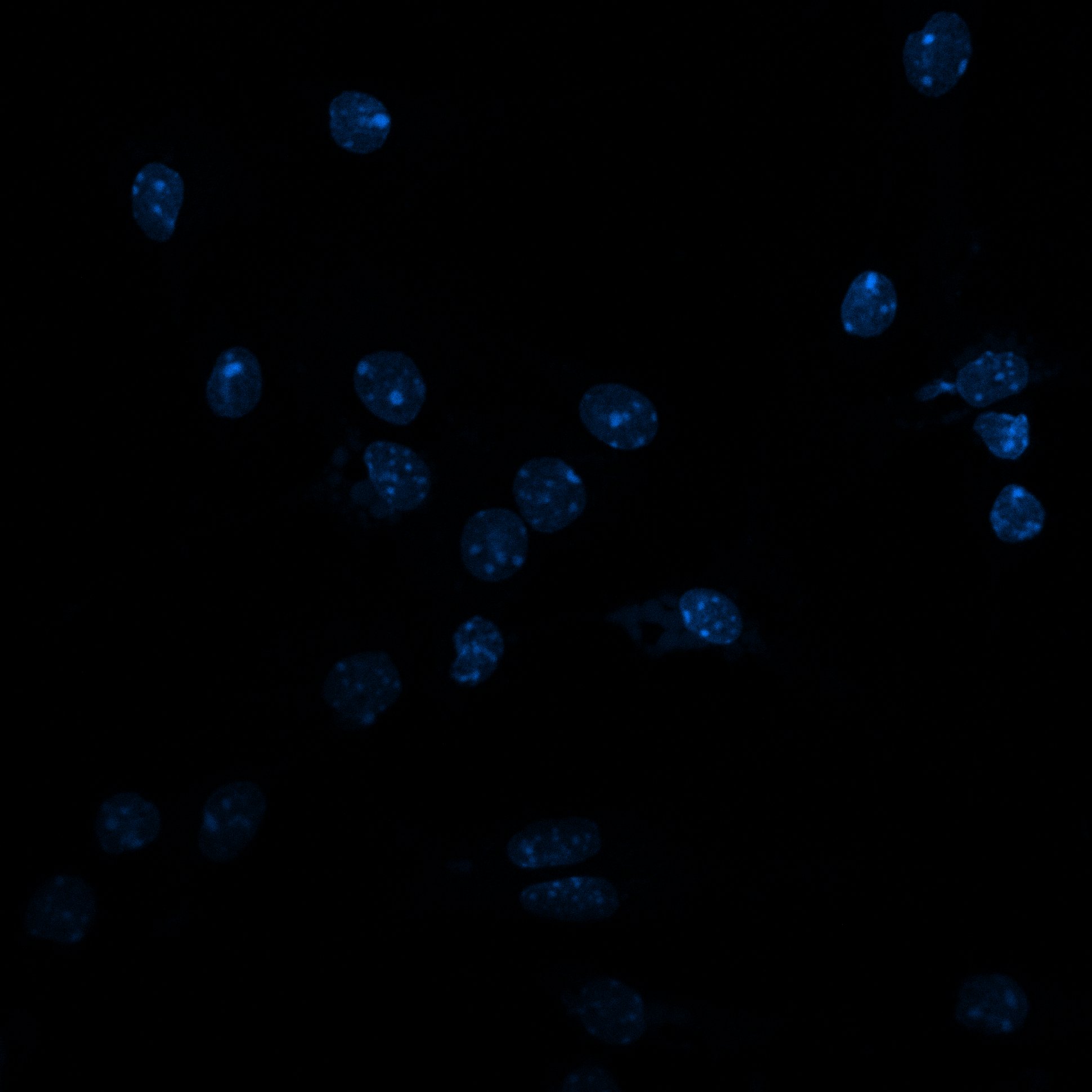

Supplement: Supplementary file 22 — Figure EV2B Source Data [file 44319_2025_673_MOESM22_ESM.zip › EV2B/Hb/Hb_hoechst staining.jpg]

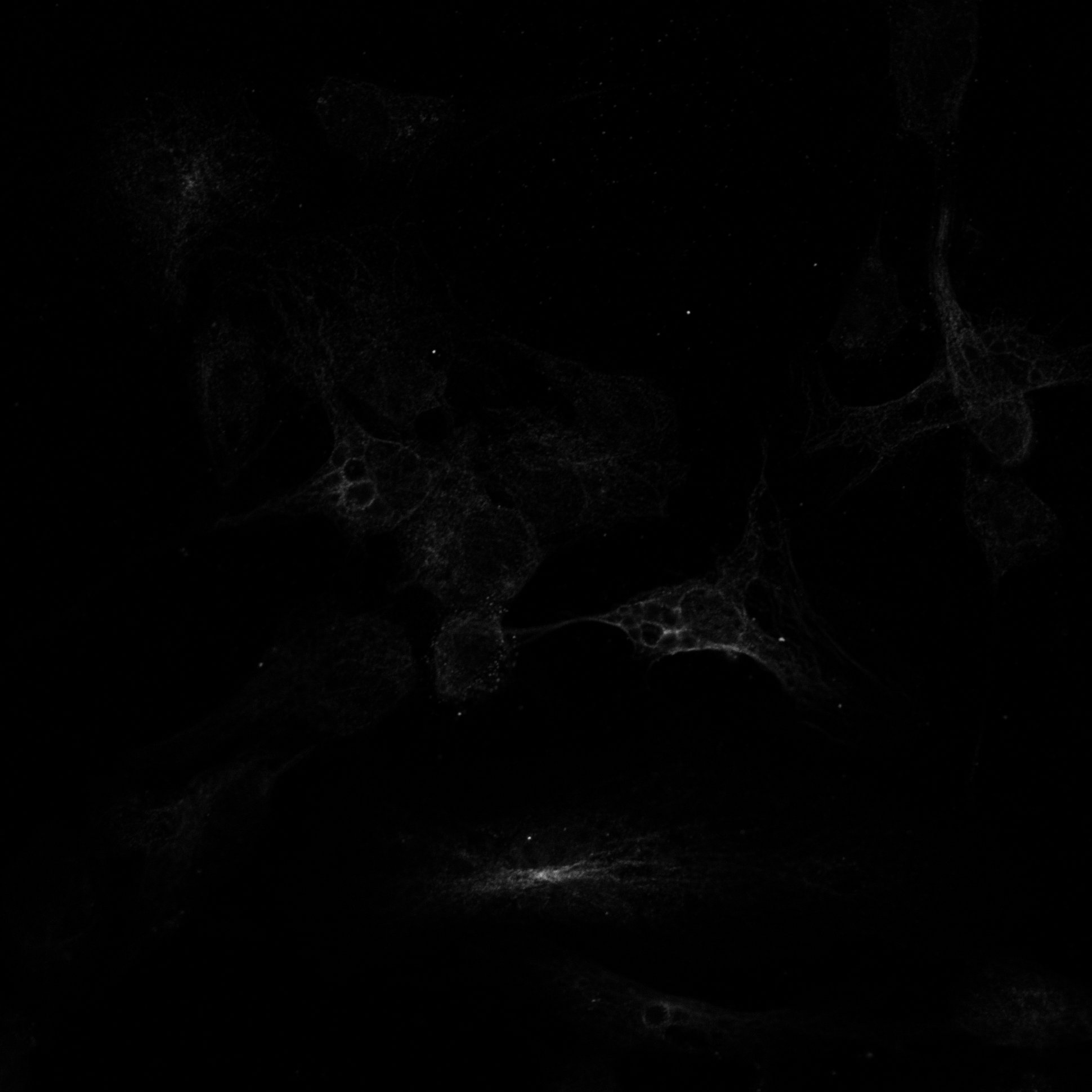

Supplement: Supplementary file 22 — Figure EV2B Source Data [file 44319_2025_673_MOESM22_ESM.zip › EV2B/Hb/Hb_Stab2 staining.jpg]
