# Supplementary material for: Liver sinusoidal endothelial cells constitute a major route for hemoglobin clearance
Source: EMBO Rep. 2026 Jan 6;27(3):598–628. doi: 10.1038/s44319-025-00673-5 (PMC12895045; doi:10.1038/s44319-025-00673-5)
Supplement: Supplementary file 19 — Appendix Figure S3 Source Data [file 44319_2025_673_MOESM19_ESM.zip › Appendix Figure S3/KCs isolation/README.docx]

The images were processed using ImageJ software with linear adjustments of contrast and brightness, equally across the whole image area and in comparison to the blank samples.
