# Supplementary material for: Liver sinusoidal endothelial cells constitute a major route for hemoglobin clearance
Source: EMBO Rep. 2026 Jan 6;27(3):598–628. doi: 10.1038/s44319-025-00673-5 (PMC12895045; doi:10.1038/s44319-025-00673-5)
Supplement: Supplementary file 23 — Expanded View Figures [file 44319_2025_673_MOESM23_ESM.pdf]

Expanded View Figures

Figure EV1. LSECs represent the major cell type that sequesters Hb.

(**A**, **B**) Hemoglobin (Hb) distribution in control and macrophage-depleted mice (clodronate) injected with AlexaFluor 750 labeled Hb (Hb-AF750, 10 μg/mouse), imaged with Bruker in vivo Imaging System. (**A**) The efficiency of macrophage depletion in the liver was examined by the percentage of liver KCs in control and clodronate-injected mice. (**B**) Representative images of organs isolated from Hb-AF750 (10 μg/mouse, 1 h) i.v.-injected mice. (**C**) Frozen liver slices from mice injected with Hb-AF647 (red) were processed and stained for Kupffer cells (KCs) (F4/80, green) or LSECs (CD146, green) and nuclei (blue). Merged-channel images for this figure are presented in Fig. 1C. (**D**) Murine NPCs in vitro cultures were treated with Hb-AF750 (0.5 μg/ml) for 1 h. Normalized Hb-AF750 fluorescence intensity and percentage of Hb-AF750+ LSECs and KCs, measured with flow cytometry. (**E**) Plots illustrating high mRNA expression of human LCES markers CD32B and CD36 in liver ECs, visualized using the Human Liver Cell Atlas (Guilliams et al, 2022). Data are expressed as mean ± SEM, and each data point represents one biological replicate, $n = 10$ (**A**), 4 (**D**). Welch's unpaired *t* test was used to determine statistical significance in (**A**, **D**); exact *P* values are shown on graphs.

© The Author(s)

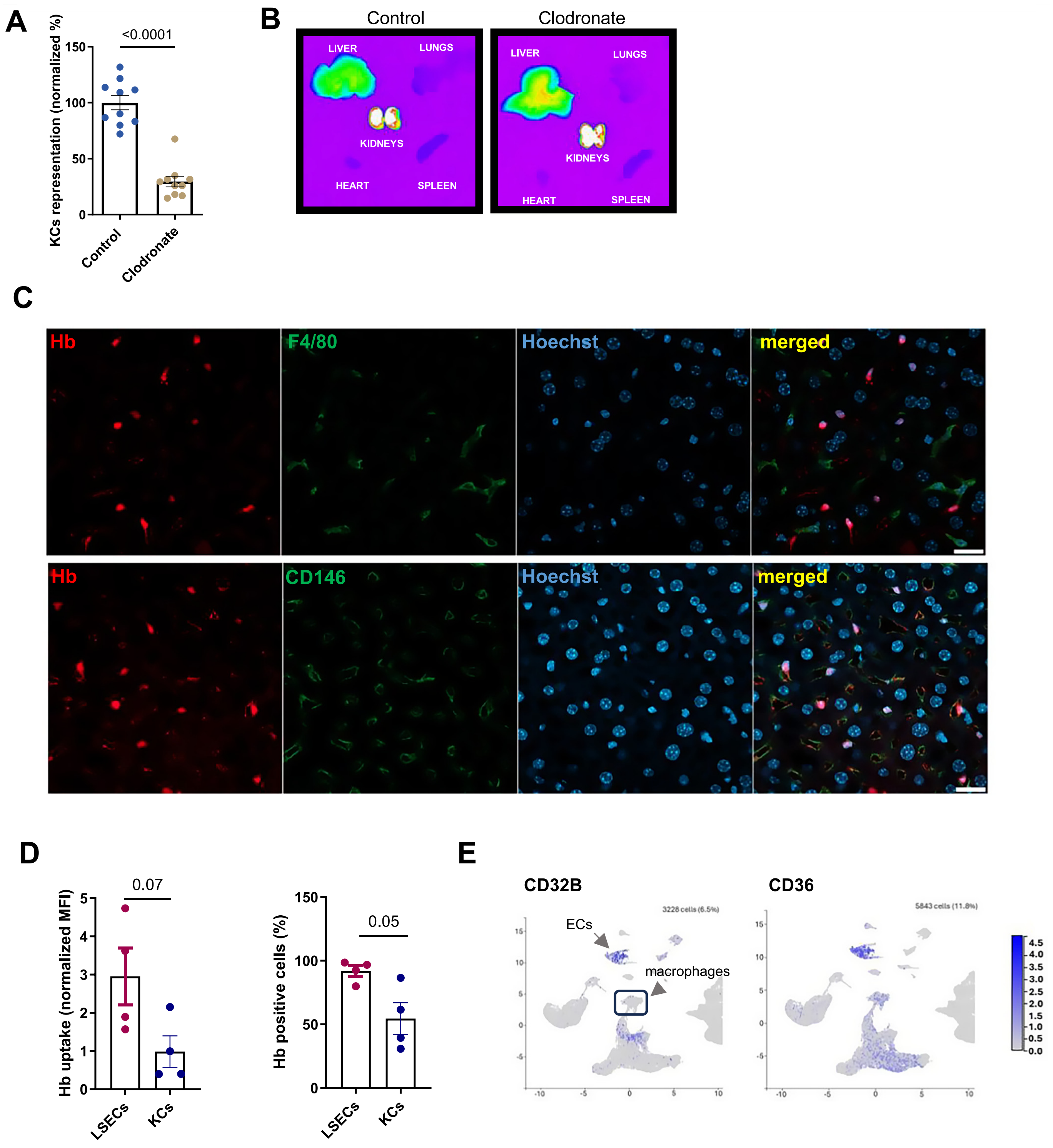
A
KCs representation (normalized %)
<0.0001
150
100
50
0
Control
Clodronate
B
Control
Clodronate
LIVER
LUNGS
KIDNEYS
HEART
SPLEEN
C
Hb
F4/80
Hoechst
merged
Hb
CD146
Hoechst
merged
D
Hb uptake (normalized MFI)
0.07
LSECs
KCs
Hb positive cells (%)
0.05
E
CD32B
CD36
ECs
macrophages

 © The Author(s)

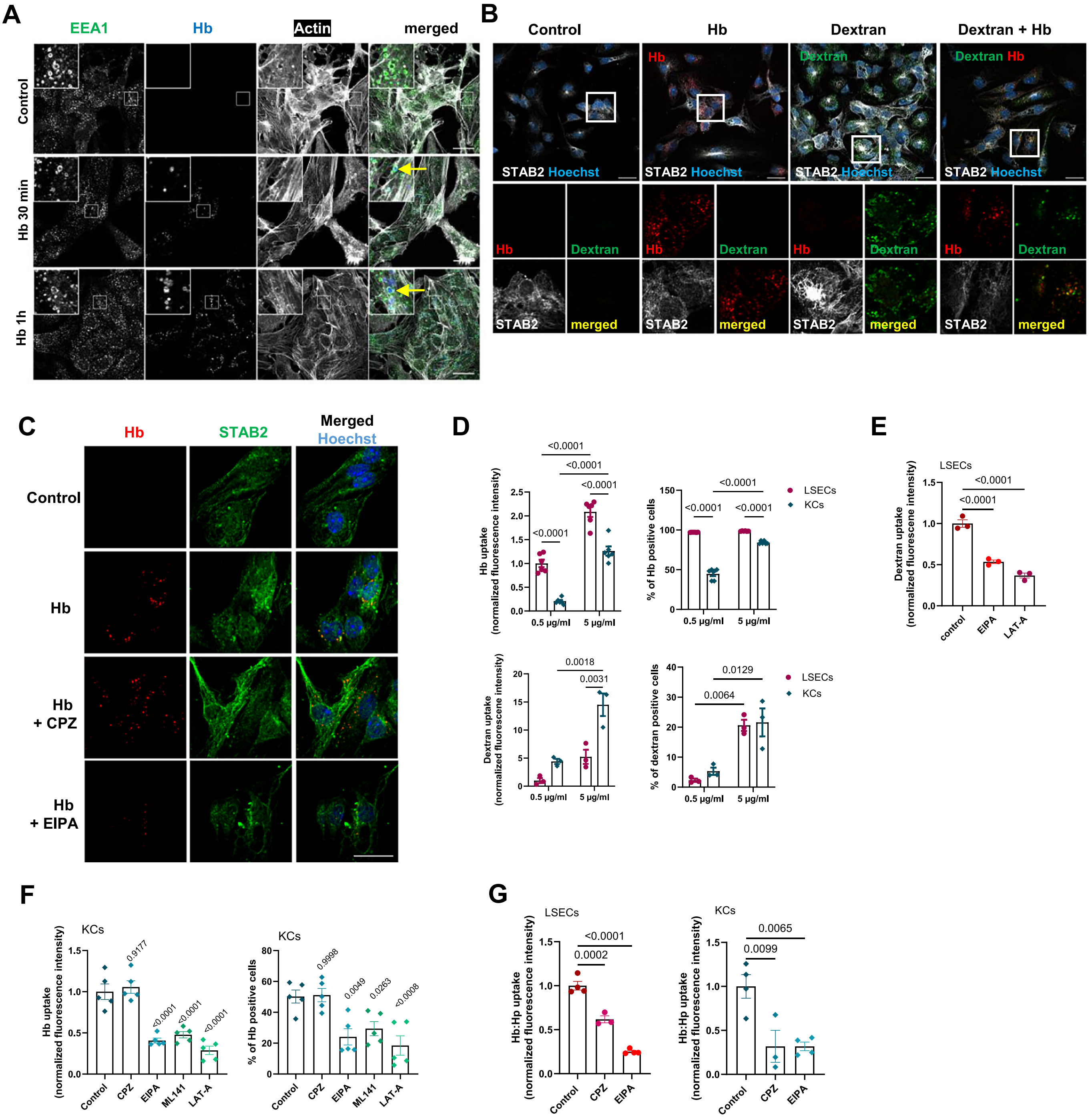
A
EEA1
Hb
Actin
merged
Control
Hb 30 min
Hb 1h
B
Control
Hb
Dextran
Dextran + Hb
STAB2 Hoechst
Dextran Hb
STAB2
merged
C
Hb
STAB2
Merged
Hoechst
Control
Hb
Hb + CPZ
Hb + EIPA
D
Hb uptake (normalized fluorescence intensity)
% of Hb positive cells
Dextran uptake (normalized fluorescence intensity)
% of dextran positive cells
0.5 µg/ml
5 µg/ml
LSECs
KCs
<0.0001
0.0018
0.0031
0.0129
0.0064
E
LSECs
Dextran uptake (normalized fluorescene intensity)
control
EIPA
LAT-A
F
KCs
Hb uptake (normalized fluorescence intensity)
% of Hb positive cells
Control
CPZ
EIPA
ML141
LAT-A
0.9177
0.9998
0.0049
0.0263
0.0008
G
LSECs
KCs
Hb:Hp uptake (normalized fluorescence intensity)
0.0002
0.0065
0.0099

Figure EV2. Primary LSECs and KCs take up Hb via macropinocytosis.

Murine NPCs in vitro cultures were treated with Hb-AF647 or fluorescently-labeled dextran (0.5 or 5 μg/ml) for 10 min, 30 min, or 1 h. (**A**) Hb-AF647 vesicle localization was imaged in NPCs in vitro cultures depleted of macrophages. Arrows indicate Hb-AF647 (blue) presence in the EEA-1+ (green) and Actin+ (white) vesicles. Areas in the highlighted rectangles are shown at higher magnification (left upper corner). Scale bars, 20 μm. Merged-channel images for this figure are presented in Fig. 2C. (**B**) Co-localization of Hb-AF647 (red) with rhodamine-dextran (green) was imaged in STAB2+ LSECs (white). The arrow indicates co-localization of Hb and rhodamine-dextran at the 10 min time-point. The area in the highlighted rectangle is shown at higher magnification in separate channels below. Nuclei were stained with Hoechst (blue). Scale bar, 20 μm. Merged-channel images for this figure are presented in Fig. 2D. (**C**) NPCs were pretreated with the inhibitor of clathrin-mediated endocytosis chlorpromazine (CPZ, 2 μM) or the macropinocytosis blocker EIPA (25 μM), before Hb-AF647 treatment for 10 min. Cells were fixed and stained for STAB2 (green) and nuclei (Hoechst, blue). Scale bars, 20 μm. (**D–F**) Hb-AF750/FITC-dextran fluorescence intensity and percentage of Hb-AF750 + /dextran+ LSECs or KCs, measured with flow cytometry 1 h after (**D**) administration of the increasing doses of the indicated cargo or (**E**, **F**) upon pretreatment with the indicated inhibitors. (**G**) Hb:Hp-AF750 fluorescence intensity in LSECs or KCs upon pretreatment with the indicated inhibitors, measured with flow cytometry 1 h after administration of Hb:Hp-AF750. Numerical data are expressed as mean ± SEM, and each data point represents one biological replicate, $n = 3–6$ (**D**), 3 (**E**), 5 (**F**), 3–4 (**G**). Two-way ANOVA with Tukey's Multiple Comparison test was used to determine statistical significance in (**D**); one-way ANOVA with Tukey's Multiple Comparison test was used to determine statistical significance in (**E–G**); exact *P* values are shown on graphs, values above bars in (**F**) indicate comparison with control cells. Source data are available online for this figure.

 © The Author(s)

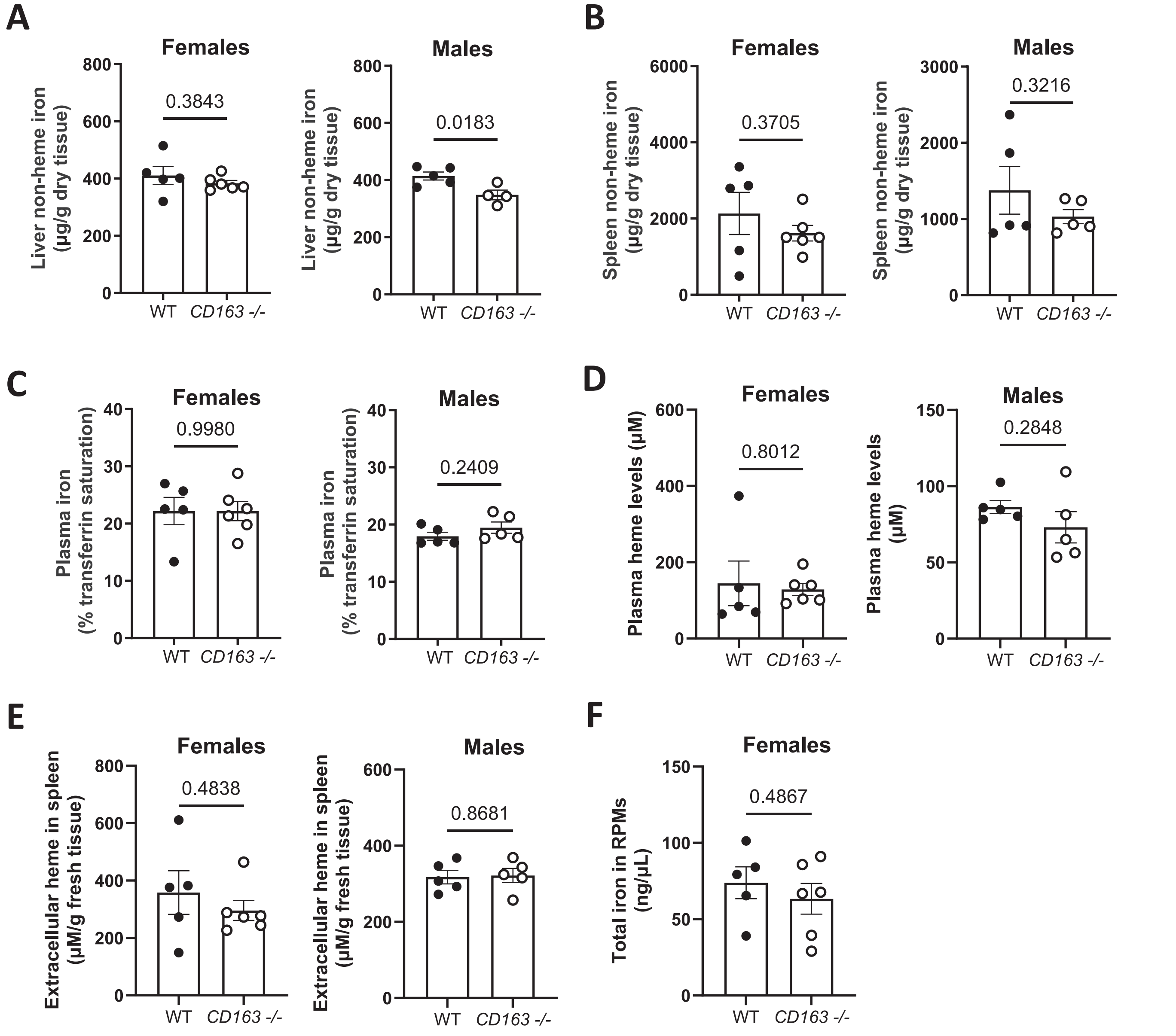

Figure EV3. CD163 KO mice show no major differences in systemic and splenic iron parameters.

(**A–E**) The phenotype of Cd163−/− mice was compared with wild-type (WT) littermates. (**A**, **B**) Non-heme iron content in (**A**) the liver and (**B**) spleen of female and male mice. (**C**) Plasma iron levels were determined by transferrin saturation measurements. (**D**, **E**) Heme levels were measured in the (**D**) plasma and (**E**) extracellular fluid from the spleen using Heme Assay Kit. (**F**) Total iron levels in magnetically-sorted RPMs were measured with Iron assay kit. Data are expressed as mean ± SEM, and each data point represents one biological replicate, $n = 4$–6 (**A**, **C**), 5–6 (**B**, **D**, **F**), 5 (**E**). Welch's unpaired *t* test was used to determine statistical significance; exact *P* values are shown on graphs.

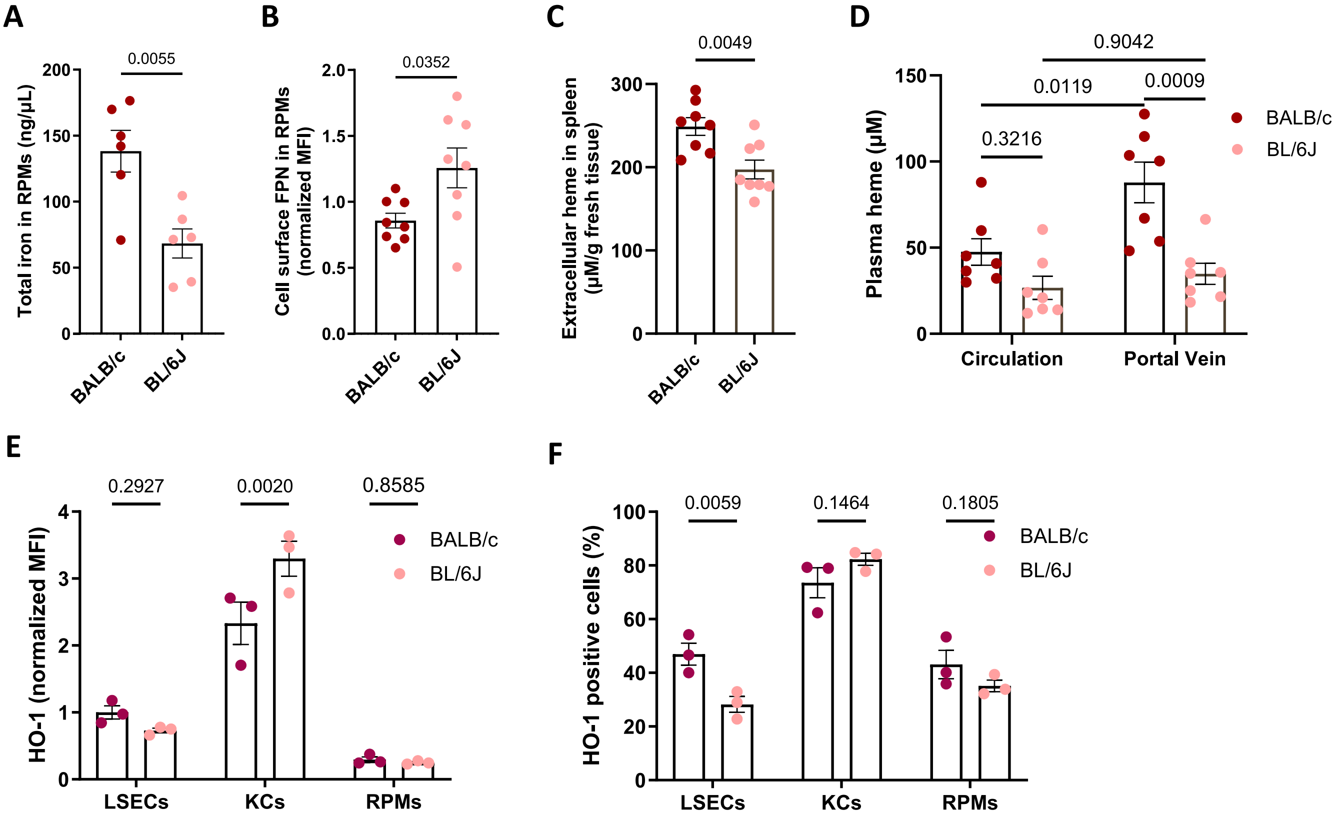

Figure EV4. Comparison of iron-recycling parameters between BALB/c and C57BL/6J mice.

(**A**) The total intracellular iron content in magnetically-sorted RPMs was assessed using the Iron Assay Kit. (**B**) FPN surface levels were measured in RPMs by flow cytometry. (**C**) Extracellular heme content in the spleen and (**D**) heme levels in the portal vein and circulating plasma were measured using Heme Assay Kit. (**E**, **F**) HO-1 levels and percentage of HO-1 positive cells in single cell suspensions from respective organs of BALB/c and C57BL/6J (BL/6J) were determined using flow cytometry. Data are expressed as mean ± SEM, and each data point represents one biological replicate, $n = 6$ (**A**), 8 (**B**, **C**), 7 (**D**), 3 (**E**, **F**). Welch's unpaired *t* test was used to determine statistical significance in (**A–C**), while two-way ANOVA with Tukey's Multiple Comparison tests was used in (**D–F**); exact *P* values are shown on graphs.

 © The Author(s)

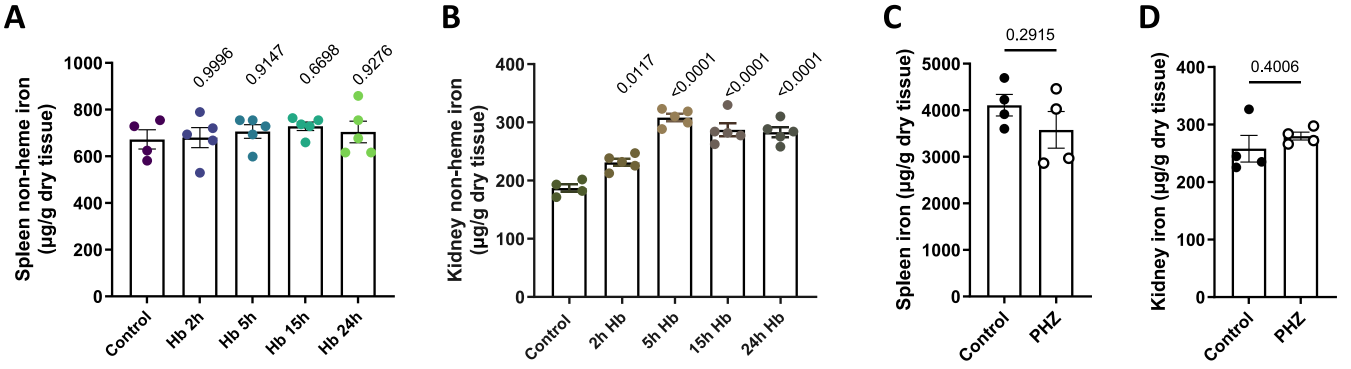

Figure EV5. Alterations of splenic and renal iron levels upon Hb injection and PHZ-induced hemolysis.

(**A**, **B**) Mice were injected with Hb (10 mg/mouse) for the indicated time points. Non-heme iron content in the (**A**) spleens and (**B**) kidneys. (**C**, **D**) Hemolysis was induced by i.p. injection of phenylhydrazine (PHZ, 0.125 mg/g) for 6 h. Non-heme iron content in the (**C**) spleens and (**D**) kidneys. Data are expressed as mean ± SEM, and each data point represents one biological replicate, $n = 4$–5 (**A**, **B**), 4 (**C**, **D**). Welch's unpaired *t* test was used to determine statistical significance in (**C**, **D**). One-way ANOVA with Tukey's Multiple Comparison test was used in (**A**, **B**); exact *P* values are shown on graphs, values above bars in (**A**, **B**) indicate comparison with control mice.
